# A Randomized Trial Evaluating Prosaptide™ for HIV-Associated Sensory Neuropathies: Use of an Electronic Diary to Record Neuropathic Pain

**DOI:** 10.1371/journal.pone.0000551

**Published:** 2007-07-25

**Authors:** Scott R. Evans, David M. Simpson, Douglas W. Kitch, Agnes King, David B. Clifford, Bruce A. Cohen, Justin C. McArthur, for the Neurologic AIDS Research Consortium and the AIDS Clinical Trials Group

**Affiliations:** 1 Harvard School of Public Health, Boston, Massachusetts, United States of America; 2 Mount Sinai School of Medicine, New York, New York, United States of America; 3 Johns Hopkins University, Baltimore, Maryland, United States of America; 4 Washington University, St. Louis, Missouri, United States of America; 5 Northwestern University, Chicago, Illinois, United States of America; Brown University Medical School, United States of America

## Abstract

**Objectives:**

To examine the efficacy and safety of Prosaptide™ (PRO) for the treatment of painful HIV-associated sensory neuropathies (HIV-SN).

**Design:**

A randomized, double-blind, placebo-controlled, multicenter study in participants with sensory neuropathy. Pain modulating therapy was discontinued prior to baseline. Participants were stratified by sural sensory nerve action potential (SNAP) amplitude. Participants were trained to use an electronic diary (ED) to record pain.

**Setting:**

Peripheral neuropathies are common complications of HIV infection. The pathogenesis is unknown and currently treatments are restricted to symptomatic measures. We examined PRO against placebo (PBO) for treatment of painful HIV-SN and performed a post-hoc evaluation of an electronic diary (ED) to record HIV-associated neuropathic pain.

**Participants:**

Eligible participants included adults with neurologist-confirmed painful HIV-SN.

**Interventions:**

2, 4, 8, or 16 mg/d PRO or PBO administered via subcutaneous (SC) injection for six weeks. Neurotoxic antiretroviral drug usage was held constant.

**Outcome Measures:**

Changes from baseline in the weekly average of evaluable daily random prompts measuring pain using the Gracely pain scale and adverse events.

**Results:**

237 participants were randomized. The study was stopped after a planned futility analysis. There were no between-group differences in the frequency of adverse events or laboratory toxicities. The 6-week mean (sd) Gracely pain scale changes were −0.12 (0.23), −0.24 (0.35), −0.15 (0.32), −0.18 (0.34), and −0.18 (0.32) for the 2, 4, 8, 16 mg, and PBO arms respectively. A similar variability of pain changes recorded using the ED were noted compared to previous trials that used paper collection methods.

**Conclusions:**

6-week treatment with PRO was safe but not effective at reducing HIV-associated neuropathic pain. Use of an ED to record neuropathic pain is novel in HIV-SN, resulted in reasonable compliance in recording pain data, but did not decrease the variability of pain scores compared to historical paper collection methods.

**Trial Registration:**

Current Controlled Trials NCT00286377

## Introduction

Sensory neuropathy is the most frequent neurological complication of HIV infection or its treatment with antiretroviral agents. Despite recent declines in the incidence rates of HIV-associated dementia and CNS opportunistic infections [1], [2] sensory neuropathies (HIV-SN) have increased in prevalence to become the most common neurological disorders associated with AIDS [3]. Two frequent types of peripheral sensory neuropathy are seen in HIV-infected patients, distal HIV-associated sensory polyneuropathy (DSP) and antiretroviral toxic neuropathy (ATN), which together, affect up to 30% of participants with advanced HIV disease [4], [5]. ATN shares most of the clinical features of DSP but is associated with specific dideoxynucleoside analogue usage and may improve with discontinuation of the drug.

The most common symptom of HIV-SN is spontaneous or evoked pain or dysesthetic sensations in the feet. The pathology of DSP involves a length-dependent degeneration of peripheral nerve fibers affecting both small and large nerve fibers, but the pathogenesis is unknown [5]–[7].

The pathogenesis of ATN is thought to reflect the selective ability of the dideoxynucleoside analogues to inhibit gamma DNA polymerase, reduce mitochondrial DNA content, and lead to mitochondrial dysfunction [8]. Elevated serum lactate levels have been associated with ATN [9], and mitochondrial DNA levels in subcutaneous fat obtained by punch skin biopsies are also reduced after exposure to d4T and ddI [10].

Although patients with HIV-associated neuropathic pain represent a large and growing participant population, they remain underserved, with no FDA-approved therapies and relatively few pharmaceutical clinical trials. Treatment is limited to symptomatic measures, with limited efficacy in pain reduction [11]. In addition, despite the reduced use of these agents in developed countries because of their toxic effects on the peripheral nervous system, they remain a critically important component of generic fixed-dose regimens in resource-limited countries. In fact, world-wide, the commonest antiretroviral treatment is D4T/3TC/nevaripine. Thus information about the toxicity of these agents remains highly relevant.

Saposins are a group of small glycoproteins that activate lysosomal hydrolysis of a variety of sphingolipids and are mutated in saposin-deficient human storage diseases. Prosaposin, the protein precursor of saposin A, B, C, and D, was identified as a neurotrophic factor. Prosaptide™ (PRO) is a 14-mer peptide, synthesized from the neuroactive region of saposin C1–5 [12]. PRO was found to be active in several *in vivo* neuropathic pain models [13]–[15]. PRO has also demonstrated a beneficial effect in animal models of neuropathy, including both type 1 (streptozotocin) and type 2 (galactose feeding) diabetes, and paclitaxel-induced toxic neuropathy [12], [16]–[17].

In two phase I clinical trials, subcutaneous injections at doses up to 300 µg/kg of PRO were found to be safe in healthy controls. Pharmacokinetic analysis revealed that PRO was rapidly absorbed, had a short half-life, and showed no accumulation after repeated dosing.

A randomized, PBO-controlled phase II trial (FDA IND number 66074) was conducted to study PRO for the relief of neuropathic pain associated with diabetes mellitus (type 1 or 2). Three dose levels (1, 4, or 16 mg) of PRO or PBO were self-administered daily in a double blind fashion for 28 days by subcutaneous injection (unpublished). A statistically significant reduction in pain was noted in the 4 mg PRO arm relative to PBO. Data suggested however that the treatment effect might be attenuated in severe neuropathies, in which sufficient axonal degeneration has occurred to render sural SNAP amplitude undetectable. A similar number of treatment emergent adverse events were seen in all dose arms including PBO. No participants develop anti-PRO antibodies. Pharmacokinetic analyses showed increased blood levels of PRO with increasing dose, with no evidence of drug accumulation after 28 days of dosing.

Given that PRO was effective in the treatment of neuropathic pain caused by experimental preclinical animal models of diabetes or chemotherapy-induced neuropathies, and was also found to be safe and well-tolerated in early phase clinical trials for diabetic neuropathy, we conducted a placebo-controlled evaluation of PRO for the treatment of HIV-associated neuropathic pain. PRO is not currently in development for any neuropathies, or other human diseases, and there is no FDA NDA application. This trial was not part of a registration effort for the compound.

## Methods

The protocol for this trial and supporting CONSORT checklist are available as supporting information; see Checklist S1 and Protocol S1.

### Participants

This study enrolled adult (>18 years old) male or female participants with neurologist-confirmed painful HIV-SN (DSP or ATN). Training in the accurate recognition of HIV-SN was conducted before the study and the definitional criteria used were those developed by a consensus conference for the American Academy of Neurology in 1991 [18]. The duration of the neuropathy was unknown. We believe that most of the participants had chronic neuropathic symptoms for at least 6 months. All trial participants were on a stable antiretroviral regimen prior to entry and while on the trial.

Entry requirements included: (1) a pain level of Gracely ≥0.74 units averaged over the 2-week screening period, (2) documented HIV-1 infection, (3) stable use or non-use of dideoxynucleoside reverse transcriptase inhibitors for ≥4 months, (4) agreement to limit the use of pain-modifying agents during the study as specified by the protocol, (5) agreement not to participate in a conception process, (6) a Karnofsky performance score of ≥60, (7) written informed consent, and (8) successful completion of ≥75% of ED endpoints in the screening period.

Participants with the following neurologic conditions were excluded: (1) any condition other than HIV infection or antiretroviral therapy that could confound the diagnosis of HIV neuropathy, (2) received insulin or oral hypoglycemic products for treatment of diabetes mellitus ≤30 days (dietary control for diabetes was allowed), (3) a history of documented vitamin B12 deficiency with less than three months of B12 supplementation prior to screening, (4) hereditary neuropathy, (5) compression-related neuropathies, (6) use of any drug other than the dideoxynucleoside analogues that might have significantly contributed to the neuropathy, (7) a history of any alcohol-related medical complications, (8) neurotoxic chemotherapeutic agents ≤90 days before study entry, (9) neuroregenerative agents ≤90 days before study entry, or (10) presence of myelopathy.

Participants with the following conditions were also excluded: (1) an active AIDS-defining opportunistic infection (OI) or OI-defining condition ≤30 days before study entry, (2) active major disease, both HIV-related and non-HIV-related including, but not limited to, cardiac disease, pulmonary, or hepatorenal, (3) pregnant or breast-feeding, (4) current active malignancy, (5) allergy/sensitivity to PRO, acetaminophen, or its formulations, (6) received any investigational agent(s) that is not FDA-approved or has participated in any interventional research study ≤30 days before study entry, or (7) actively using recreational intravenous drugs, crack cocaine, or intranasal/smoked heroin or methamphetamine.

Participants with the following laboratory abnormalities were also excluded: (1) absolute neutrophil count (ANC) <750/mm^3^ (<0.75×109/L), (2) hemoglobin <8.0 g/dL for males or <7.5 g/dL for females, (3) platelet count <75,000/mm^3^ (<75×109/L), (4) creatinine >1.5× upper limit of normal (ULN), (5) AST (SGOT), ALT (SGPT), and alkaline phosphatase >5× ULN, (6) total bilirubin >1.5× ULN (participants receiving indinavir, atazanavir, or other drugs with the same known effect on bilirubin levels were eligible if total bilirubin was <5× ULN), (7) HgbA1C >6.5, or (8) serum B12 ≤200 pg/mL. Note that these exclusionary criteria likely excluded most participants with active hepatitis C coinfection.

### Interventions

The intervention in this study was six-week treatment with PRO or matching placebo for the treatment of HIV-associated neuropathic pain. The rationale for the 6 weeks duration was that this was the maximal length of patient exposure allowable by FDA based on available toxicity data. Participants were randomly assigned to 2, 4, 8, or 16 mg/d PRO or PBO administered via SC injection.

### Objectives

The objective of this study was to examine the efficacy and safety of PRO for the treatment of painful HIV-SN compared to PBO after six weeks of treatment. A post-hoc objective was to evaluate the use of an ED to record HIV-associated neuropathic pain.

### Outcomes

The primary endpoint in this trial was the 6-week change from baseline in the weekly average of evaluable daily random prompts measuring pain using the Gracely pain scale. Secondary endpoints included “treatment success”, defined as ≥0.35 units of pain improvement from baseline on the Gracely scale, and change in HIV viral load. Safety endpoints included treatment emergent serious adverse events (SAEs), AEs, and toxicities.

### Sample Size

The study was originally designed to randomize 390 participants, equally allocated between groups. The study was sized such that the 95% confidence interval for the difference between any dose arm and PBO with respect to changes in the 13-point Gracely pain scale was no wider than 0.24 assuming a standard deviation of Gracely pain scale changes of 0.35 (i.e., an estimate derived from earlier studies) [19]–[20]. An interim analysis was planned after 200 participants completed the 6-week double-blind treatment period to evaluate safety, futility, sample size assumptions made in design of the trial, and the effect of sural SNAP amplitude on pain changes.

### Design

NARC 009/Savient C0603/ACTG A5180 was a prospective, randomized, double-blind, PBO-controlled, multicenter study. The study was conducted at member sites of the AIDS Clinical Trials Group and Neurologic AIDS Research Consortium with neurological expertise, and at other community-based trial sites. The study was approved by all study site Institutional Review Boards.

Participants with at least a moderate pain rating (Gracely pain scale >0.74) and who had completed written informed consent were stratified according to sural SNAP amplitude at baseline (negative: 0–4 :V vs. positive: >4 :V) as a surrogate of baseline nerve fiber damage and then randomized to 2, 4, 8, or 16 mg/d PRO or PBO administered via SC injection. A blinded, centralized laboratory provided quality control by monitoring all wave forms in sural SNAP amplitudes at each site. The stratification according to sural SNAP amplitude was made because the earlier clinical trial in diabetic neuropathy had suggested a greater therapeutic effect on participants with detectable sural SNAP amplitude than those with absent sural SNAP amplitude. The cut-off of 4 microV was made because this is considered to be lower limit of normal in most electrophysiology laboratories. Adjuvant pain medications including anticonvulsants, antidepressants, topical analgesics and short-acting narcotics were washed out prior to randomization and not permitted during the study period. Study drug (provided by Savient Pharmaceuticals, Inc.) was given for six weeks. Neurotoxic antiretroviral drugs were continued at baseline dosage throughout the study period.

At each site, up to 20% of enrolled participants were permitted to continue chronic daily doses of long acting opioid analgesics. The justification for this was that we felt it would be impractical to safely taper narcotics during a short pre-randomization wash-out period, and we did not want to completely exclude narcotic-using participants who met entry criteria in all other regards. All participants received a supply of acetaminophen 500 mg caplets as rescue medication. Participants who had intolerable pain despite rescue medication were permitted to discontinue from the study at any time.

The study consisted of a two-week washout period, randomization, a 6-week double-blind treatment period, and a two-week double-blind cross-over. During the wash-out period, any pain-modifying agents were tapered and discontinued prior to randomization. A standardized neurological exam, neuropathy assessment, and CD4 and HIV-1 RNA evaluations were performed at baseline and at the end of the double-blind treatment period. Pain (Gracely pain scale and the Visual Analogue Scale (VAS)) was measured several times daily.

### Randomization-Sequence Generation

The randomization schedule was created using permuted blocks using a block size of 5 (corresponding to the five treatments). Randomization was stratified by SNAP status (2 levels: negative: 0–4 :V vs. positive: >4 :V) and was assigned in a ratio of 1∶1∶1∶1∶1.

### Randomization-Allocation Concealment

Each kit (1 kit per participant) was assigned a kit number. The kit number could be translated through the randomization sequence which was stored under lock and key by Savient Quality Assurance. Randomization was concealed to the clinical sites.

### Randomization-Implementation

Generation of the allocation sequence was made by Savient Pharmaceuticals, Inc. for packaging to site pharmacists. Savient Quality Assurance Department locked the codes in a secured area. Site pharmacists assigned patient numbers in a sequential order and provided appropriate study drug as participants were enrolled into the study.

### Blinding

Study participants, study personnel administering interventions and assessing outcomes, and investigators were blinded to treatment assignment. Unblinding occurred only after database closure. No blinding questionnaire was used.

### Electronic Diary

Participants were issued a Palm Pilot ED and trained in its use. The ED was used to obtain pain data in real time. Data captured on the ED included the Gracely pain scale, study medication dosing, and rescue medication use. In addition, a morning assessment of sleep quality and an evening daily pain score were captured.

Random prompts were presented to the participants approximately four to six times per day. The participants were asked to rate their current level of pain using the modified Gracely pain scale. Functions of the ED included: (1) a “suspend” function allowing suspension of random prompting for up to two hours, in anticipation of being in a situation where they should not be prompted, and (2) a sleep feature enabling participants to “turn off” the ED during sleep. Prior to each study visit, the participant's study compliance in responding to prompts was reported to the site and used as the basis of providing feedback to study participants.

Data were uploaded from each ED on a nightly basis by telephone to a central database. ED programming and data management was performed by Invivodata, Inc (Pittsburgh, PA).

### Statistical Methods

Descriptive statistics were used to describe the study sample and confidence intervals were used to estimate population parameters. Graphical methods were used to display changes over time. Predicted intervals were utilized at the interim futility analysis. The futility analyses were planned to examine if there was early evidence that significant results were unlikely, thus providing a cost-efficiency check. The primary analysis utilized a modified intent-to-treat (ITT) approach. The ITT population was defined as all randomized participants that received at least one dose of study medication. A last-observation-carried-forward (LOCF) imputation was utilized for missing data, however several sensitivity analyses (e.g., analyses of observed non-missing data) were performed to ensure the robustness of the results. The signed-rank test was used to assess the significance of within-arm changes with respect to continuous variables whereas the Wilcoxon rank sum test was used to assess between arm differences. Wei-Johnson tests [21] were used to compare treatment groups across timepoints. All reported p-values are 2-sided without adjustment for multiple testing. Statistical significance was assessed using significance level of 0.05.

## Results

### Participant Flow, Recruitment, and Numbers Analyzed

**Figure 1:**
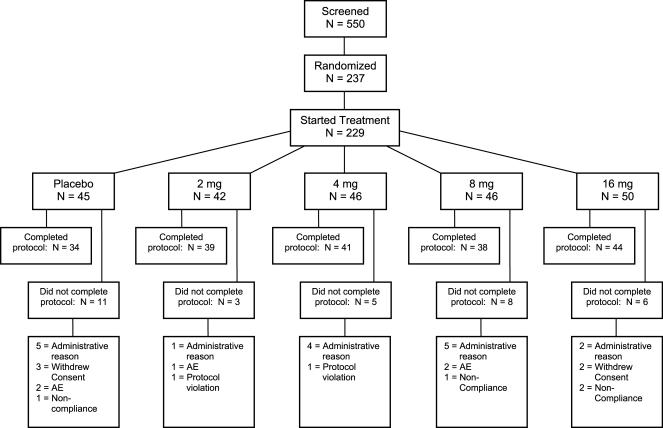
CONSORT Diagram.

196 of 237 randomized participants from 31 sites completed the double-blind period (Figure 1). The first participant was enrolled in August, 2003. 17 participants did not complete the study for administrative reasons (e.g., early termination of the study in March, 2005). We report results based on 229 participants that received study drug (modified ITT population).

### Baseline Data

No clinically relevant between-group differences in demographics or baseline characteristics were observed (Table 1).

**Table 1:** Demographics and Baseline Characteristics by Treatment.

		Treatment				
	Total	Placebo	2 mg	4 mg	8 mg	16 mg
N	229	45	42	46	46	50
Age (years)						
Median	47	46	49	46.5	47.5	47
Q1, Q3	43,53	43, 51	44, 53	45, 54	42, 54	40,53
Race N(%)						
American Indian/Alskn Native	1 (0%)	0 (0%)	1 (2%)	0 (0%)	0 (0%)	0 (0%)
White	117 (51%)	24 (53%)	23 (55%)	23 (50%)	22 (48%)	25 (50%)
Black or African American	81 (35%)	15 (33%)	14 (33%)	15 (33%)	20 (43%)	17 (34%)
Hispanic or Latino	27 (12%)	6 (13%)	4 (10%)	7 (15%)	3 (7%)	7 (14%)
Native Hawaiian/Pacific Islndr	1 (0%)	0 (0%)	0 (0%)	1 (2%)	0 (0%)	0 (0%)
Other	2 (1%)	0 (0%)	0 (0%)	0 (0%)	1 (2%)	1 (2%)
Sex N(%)						
Male	210 (92%)	41 (91%)	41 (98%)	41 (89%)	40 (87%)	47 (94%)
Female	19 (8%)	4 (9%)	1 (2%)	5 (11%)	6 (13%)	3 (6%)
Sural SNAP Amplitude N(%)						
>4:V	86 (38%)	17 (38%)	12 (29%)	19 (41%)	17 (37%)	21 (42%)
≤ 4:V	143 (62%)	28 (62%)	30 (71%)	27 (59%)	29 (63%)	29 (58%)
CD4 (cells/:L)						
Median	367	312.5	393.5	447	308	392
Q1, Q3	238, 586	192, 421	260.5, 691	274, 641	214, 565	246, 540
Log_10_ HIV-1 RNA (copies/mL)						
Median	3.06	2.99	3.32	2.90	3.05	3.09
Q1, Q3	2.61, 3.71	2.61, 3.49	2.90, 3.98	2.60, 3.34	2.60, 4.27	2.65, 4.03
Gracely Pain Scale (log, 13 point)						
Median	1.22	1.24	1.17	1.16	1.17	1.35
Q1, Q3	1.07, 1.42	1.09, 1.43	0.99, 1.34	1.05, 1.36	1.06, 1.36	1.09, 1.57
VAS Pain Scale						
Median	69	69	68.5	65	64.5	73.5
Q1, Q3	59, 78	58.5, 78	57, 76	59, 75	57, 74	62, 84
Continuing on Opioids N(%)						
No	210 (92%)	42 (93%)	36 (86%)	41 (89%)	44 (96%)	47 (94%)
Yes	19 (8%)	3 (7%)	6 (14%)	5 (11%)	2 (4%)	3 (6%)
Karnofsky Score N(%)						
60	10 (4%)	4 (9%)	0 (0%)	2 (4%)	2 (4%)	2 (4%)
70	28 (12%)	4 (9%)	6 (14%)	3 (7%)	6 (13%)	9 (18%)
80	116 (51%)	21 (47%)	25 (60%)	21 (46%)	25 (54%)	24 (48%)
90	56 (24%)	11 (24%)	9 (21%)	16 (35%)	11 (24%)	9 (18%)
100	19 (8%)	5 (11%)	2 (5%)	4 (9%)	2 (4%)	6 (12%)
ddC, d4T, or ddI Use at Entry N(%)						
No	177 (77%)	37 (82%)	33 (79%)	34 (74%)	36 (78%)	37 (74%)
Yes	52 (23%)	8 (18%)	9 (21%)	12 (26%)	10 (22%)	13 (26%)
Weeks on Antiretroviral Therapy at Entry						
Median	65	58	88	79	57	59
Q1, Q3	29, 136	29, 93	34, 148	29, 147	26, 116	34, 141

### Interim Analysis Summary

A planned interim analysis was performed to evaluate safety, futility, sample size assumptions made in design of the trial, and the effect of sural SNAP amplitude on pain changes. After a review of the interim results, the Data Safety Monitoring Board (DSMB) of the Neurologic AIDS Research Consortium (NARC) recommended termination of the study based on a futility analysis which indicated that even if the trial were to continue to its planned completion with full accrual, there would be a very low probability of attaining statistical significance with regard to the analgesia efficacy endpoint for any of the dosing arms compared to PBO. No safety issues were identified. Sample size assumptions made in the design phase of the study were determined to be valid. In particular, the assumed variability of the change in Gracely pain scale was determined to be reasonable. Sural SNAP amplitude (the stratification variable) appeared to have no effect on the change in Gracely pain score (primary endpoint).

### Outcomes and Estimation

**Figure 2:**
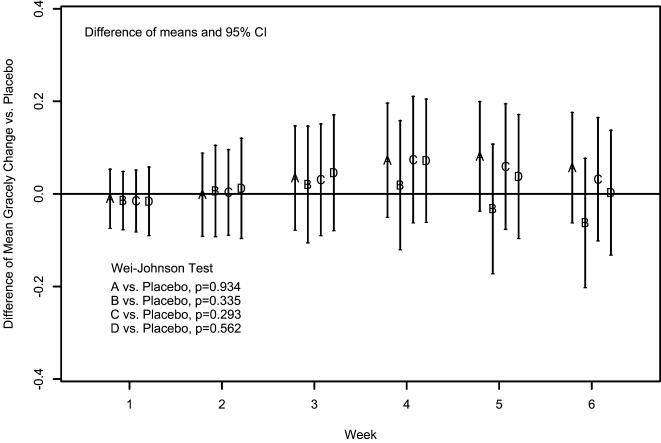
Difference of Mean Gracely Pain Score Changes (Using LOCF) between each Dosing Group and Placebo across Weeks.

Pain decreased in all arms. The 6-week mean (sd) Gracely pain scale changes (using LOCF) were −0.12 (0.23), −0.24 (0.35), −0.15 (0.32), −0.18 (0.34), and −0.18 (0.32) for the 2, 4, 8, 16 mg, and PBO arms respectively. No statistically significant differences between any PRO arm and PBO were noted (Figure 2). LOCF imputation was required on 33/229 (14.4%) observations. Sensitivity analyses using other methods for missing data yielded similar results, as did treatment comparisons of pain changes assessed using the VAS. There was insufficient evidence to conclude that the “treatment success” rates for the 2 mg (19%), 4 mg (28 %), 8 mg (22 %), and 16 mg (28%), were different from PBO (22%). The median number of times that rescue medication was used per day during the study was comparable: placebo (0.26), 2 mg (0.44), 4 mg (0.45), 8 mg (0.48), and 16 mg (0.38).

### Adverse Events and Safety Outcomes

Five SAEs were reported after the 1^st^ dose of study drug: two in the 4 mg arm (i. cellulitis, unlikely related (judged by site investigator), moderate severity, resolved; ii. shigella enteritis, likely unrelated), one in the 8 mg arm (altered mental status, likely unrelated, moderate severity, resolved), one in the 16 mg arm (pancreatitis, likely unrelated, severe, resolved), and one in the PBO arm (Kaposi's sarcoma, likely unrelated, mild severity, resolved). No deaths were reported. There was no difference in the frequency of adverse events or laboratory toxicities between PRO and PBO. Four grade 3 (severe) AE's were reported: two in the placebo arm (i. foot pain, possibly related; ii. thrombocytopenia, possibly related), one in the 2 mg arm (fatigue, unrelated), and one in the 16 mg arm (thrombocytopenia, unlikely related). Five grade 4 laboratory toxicities were reported: two in the placebo arm (1 reduced platelet count; 2. elevated calcium level), one in the 2 mg arm (elevated SGOT level), and two in the 16 mg arm (both with reduced absolute neutrophil count).

Statistically significant decreases in CD4 were noted for the 8 mg arm only; however this was not significantly different (statistically) from PBO (Table 2). No statistically significant decreases in plasma HIV-1 RNA levels were noted for the active compared to PBO.

**Table 2:** Changes in CD4 and Log_10_ HIV-1 RNA by Treatment.

		Treatment				
	Total	Placebo	2 mg	4 mg	8 mg	16 mg
CD4 (cells/:L)						
N	186	32	37	38	36	43
Median	−1.5	−3.5	−1	10.5	−36.5	6
Q1, Q3	−65, 39	−23, 31	−69, 41	−56, 40	−101, 10.5	−56, 65
p^1^	0.25	0.70	0.40	0.99	0.03	0.76
p^2^			0.68	0.70	0.07	0.55
Log_10_ HIV-1 RNA (copies/mL)						
N	166	30	36	35	32	33
Median	0.00	−0.02	−0.06	0.00	−0.06	0.00
Q1, Q3	−0.27, 0.14	−0.25, 0.19	−0.34, 0.09	−0.10, 0.35	−0.47, 0.04	−0.18, 0.05
P^1^	0.08	0.67	0.07	0.14	0.04	0.22
P^2^			0.46	0.18	0.25	0.89

1 Signed-rank test for within-arm changes

2 Wilcoxon rank sum test vs. placebo

### Ancillary Analyses

Summarization of ED use is based on 11,797 participant-days (Table 3). 98.05% of all participant-days had a morning report recorded while within participants, the average response rate was 97.08%. 89.84% of all participant-days had an evening report while within participants the average response rate was 88.38%. Forty-four participant-days did not have any random pain prompts, thus over 11,753 participant-days there were 49,561 random prompts (an average of 4.2 random prompts per day). The response rate for random prompts was 90.91%. Within participant-days the average response rate was 91.33% while within participants the average response rate was 90.37%. Use of study drugs was reported on 94.59% of all participant-days while within participants the average medication compliance rate was 92.76%. 28.51% of all participant-days utilized the suspend feature on the diary at least once while within participants the average percent of days which used the suspend feature at least once was 28.11%.

**Table 3:** Evaluation of Electronic Diary.

	Unit of Analysis		
	Prompt N = 49561	Participant Days	Participant N = 229
**Morning Report**		98.05% (N = 11797)	97.08%
**Evening Report**		89.84% (N = 11797)	88.38%
**Random Prompt**	90.91%	91.33% (N = 11753)	90.37%
**Medication Compliance**		94.59% (N = 11797)	92.76%
**Suspend Feature**		28.51% (N = 11797)	28.11%

The response rate for random prompts was similar for: males and females (91.03% and 89.44% respectively); white, black, and other races (92.27%, 88.99%, and 90.55% respectively); and for younger (≤ 45 years old) and older (>45 years old) participants (90.31% and 91.29% respectively).

The standard deviation (SD) of pain changes using the ED collection method was 0.32.

## Discussion

### Interpretation

PRO is a novel agent with preclinical evidence suggesting both analgesic and neuroregenerative potential in painful neuropathy models. This study was designed to assess only the short term analgesic potential of PRO, principally because toxicity data was not available to allow for use over a longer period. This 6-week treatment with PRO was safe and well tolerated but was not effective at reducing HIV-associated neuropathic pain relative to PBO at doses of 2, 4, 8, and 16 mg/day.

### Generalizability

The duration of the trial limits any conclusions for the regenerative potential of PRO, but do not support an acute analgesic effect in HIV-associated neuropathic pain. This trial was carefully designed to isolate analgesic effects by removing other pain-modifying therapies prior to study initiation and restricting the use of opioids. Our study sample consisted largely of males (92%) as has been observed on other clinical trials of HIV-SN [19]–[20], [22]. This is not surprising since HIV-SN tends to affect those with more advanced HIV disease [23], and proportionately more men than women have advanced HIV disease in the domestic HIV population.

### Overall Evidence

PRO was efficacious in an early trial for diabetic neuropathy. This clinical trial was the first to examine the effects of PRO for HIV-associated neuropathy. Results indicated that 6-week treatment with PRO was safe but not effective at reducing HIV-associated pain relative to placebo. Possible reasons for the negative nature of the study include that the mechanism of pain generation in HIV-SN may be very different from that in diabetic neuropathy. Also, there was a clear dose effect in the diabetic trial. It is possible that the doses in the HIV-SN trial were not high enough to observe an effect. Recently, studies [24] have suggested that protease inhibitors may be linked to the development of HIV-SN. The implications of this observation for clinical practice remain unclear and information is lacking for newer protease inhibitors. Given that this was a randomized trial, we do not expect that protease inhibitor use affects our conclusions. The regenerative potential of PRO is still unknown and it is likely that it could not be ascertained without a substantially longer trial.

Clinical evaluation of sensory experience, and especially neuropathic pain is challenging, in part because neuropathic pain intensity and quality varies considerably within a 24 hour epoch. Pain treatment trials often rely on paper and pencil tools that are recorded without observation or verification during treatment, and which typically attempt to ‘average’ pain during an epoch. The development of relatively inexpensive personal EDs make available convenient and affordable technology for prompting observations and recording responses “in the moment” throughout a study interval, as well as convenient regular accessing of responses. With the ability to collect and verify pain data using frequent observations (e.g., several random prompts per day), it is possible that a more complete and precise measure of a participants' pain profile may be recorded, resulting in a greater ability to verify clinical responses. Previous studies have suggested that EDs can permit much more accurate recording of pain than paper diaries (e.g., in a back pain study comparison of ED to paper diaries suggested a compliance rate above 91% for the ED compared to 11% with paper diaries [25]). EDs could enhance the ability to identify temporal patterns of response to interventions and to electronically confirm the timing of recorded responses. Our trial represents the first use of an ED in HIV-SN, and we initially hypothesized that the ED might reduce variability of changes in pain measurements over time and might therefore result in more accurate reporting of changes in pain. However, in our trial, the ED did not appear to decrease the variability of Gracely pain changes (SD = 0.32) compared to trials that we have conducted using written diaries (SD = 0.33 in ACTG 291 [19] and ACTG 242 [20]. We note that the variability of the outcome measure is in part a function of intra-participant variation and also the lack of effectiveness of study medication. Absent a clear clinical response to the intervention in this trial, we are limited to observations about the variability of response, which likely also has a strong biological basis in the setting of chronic pain. One caveat is that we did not directly compare ED reports to paper diaries in the same trial. While we could not confirm a decrease in the variability in pain assessment using this technology, our experience did at least support the acceptability of the ED in an HIV-SN patient population. The “sleep” and “suspend” features of the ED and the ability to train on its use may improve feasibility for use in clinical trials. While some participants found the ED device intrusive and unpleasant, in general they complied very well with it over this relatively short trial. The study sample included participants with diverse educational and social backgrounds, including those with a history of drug abuse. Similar acceptable compliance with regard to response to random prompts was noted across demographic subgroups. Thus, we believe that at least for short periods this technology could be applied in diverse groups of participants with pain, although the technology would add to the costs of a trial.

## Supporting Information

Checklist S1 CONSORT Checklist
(0.05 MB DOC)

Protocol S1 Trial Protocol
(0.84 MB DOC)
