# Supplementary material for: A Randomized Trial Evaluating Prosaptide™ for HIV-Associated Sensory Neuropathies: Use of an Electronic Diary to Record Neuropathic Pain
Source: PLoS One. 2007 Jul 25;2(7):e551. doi: 10.1371/journal.pone.0000551 (PMC1919427; doi:10.1371/journal.pone.0000551)
Supplement: Protocol S1 — Trial Protocol (0.84 MB DOC) [file pone.0000551.s002.doc]

**A collaborative study sponsored by the Neurologic AIDS Research Consortium, the department of Development and Medical Affairs of Savient Pharmaceuticals, Inc., and the Adult AIDS Clinical Trials Group**

prosaptide in neuropathic pain associated with HIV-1

NARC Protocol No. 009/Savient Protocol No. C0603/

AACTG Protocol No. A5180

# Study title: A randomized, double-blind, placebo-controlled, multicenter, dose ranging study to evaluate the efficacy and safety of prosaptide over 6 weeks of treatment for the relief of neuropathic pain associated with HIV-1

**Amendment 2**

(includes summary of the amendment and the revised protocol)

| Authors: | Zeb Horowitz, MD and Justin McArthur, MBBS, MPH |
| --- | --- |
| Document type: | Clinical study protocol |
| Development phase: | 2 |
| Document status: | Final |
| Original protocol release date: | 15 October 2002 |
| Amendment 1 release date: | 21 March 2003 |
| Amendment 2 and revised protocol release date: | June 21 2004 |
| Number of pages: | 120 |

## Signature page for Savient Pharmaceuticals, Inc.

Compound name: Prosaptide^TM^ (commonly referred as prosaptide)

**Amendment 2 for NARC Protocol No. 009/Savient Protocol No. C0603/AACTG Protocol No. A5180**

#### Corporate sponsor representative:

#### Zeb Horowitz, MD

| Sr. Vice President, Chief Medical Officer | Signature |  | Date |
| --- | --- | --- | --- |

## List of Neurologic AIDS Research (NARC) personnel

| **NARC personnel** | **Affiliation** |
| --- | --- |
| Justin McArthur, MBBS, MPH  Protocol chair | Johns Hopkins University  Department of Neurology 6-109 Meyer  600 North Wolfe Street  Baltimore, MD 21287-7609  phone: (410) 955-3730  fax: (410) 955-0672 email: [jm@jhmi.edu](mailto:jmcarth@jhmi.edu) |
| David M. Simpson, MD  Protocol vice chair | Mount Sinai School of Medicine One Gustave L. Levy Place Box 1052 New York, NY 10029-6504 phone:(212) 241-8748  fax: (212) 987-3301  email: [david.simpson@mssm.edu](mailto:david.simpson@mssm.edu) |
| Carlos Luciano, MD  Protocol vice chair | University of Puerto Rico School of Medicine  UPR-Clinical Research Center  First Floor University Hospital  GPO Box 365067  San Juan, PR 00936  phone:(787) 759-0306 x237  fax: (787) 759-0305  email: cluciano@rcm.upr.edu |
| David B. Clifford, MD  Principal investigator, NARC | Washington University in St. Louis  Department of Neurology  Campus Box 8111  660 South Euclid Avenue  St. Louis, MO 63110  phone: (314) 362-9731  fax: (314) 454-1378  email: cliffordd@neuro.wustl.edu |
| Scott Evans, PhD  Senior protocol statistician | SDAC/Harvard School of Public Health  FXB-513  651 Huntington Ave.  Boston, MA 02115  phone: (617) 432-2998  fax: (617) 432-3163  email: [evans@sdac.harvard.edu](mailto:wei@sdac.harvard.edu) |

**List of Neurologic AIDS Research (NARC) personnel** (cont’d)

| **NARC personnel** | **Affiliation** |
| --- | --- |
| Douglas Kitch, MS  Statistician | Statistician/COMP RAC  SDAC/Harvard School of Public Health  FXB Bldg. Room 504  651 Huntington Avenue  Boston, MA 02115  phone: (617) 432-3281  fax: (617) 432-3163  email: dkitch@sdac.harvard.edu |

**List of external facilities and personnel**

| **DAIDS personnel** | **Affiliation** |
| --- | --- |
| Mary E. Smith/Betsy Smith, MD | DAIDS Clinical Representative  DAIDS, NIAID, NIH  Room 5105  6700B Rockledge Drive  Bethesday, MD 20892  phone: (301) 402-3226  fax: ( )  email: MESmith@niaid.nih.gov |

| **NINDS Data Safety and Monitoring Board personnel** | **Affiliation** |
| --- | --- |
| Burk Jubelt, MD  Chairperson  Professor and Chairman | SUNY Health Science Center  University Hospital, Room 6414  Department of Neurology  750 East Adams Street  Syracuse, NY 13210  phone: (315) 464-4627  fax: (315) 464-6402  email: [jubeltb@vax.cs.hscsyr.edu](mailto:jubeltb@vax.cs.hscsyr.edu) |
| Bruce Barton, PhD Vice President  Consultant | Maryland Medical Research Institute  600 Wynhurst Avenue  Baltimore, MD 21210  phone: (410) 532-0222  fax: (410) 323-8622  email: [bbarton@mmri.org](mailto:bbarton@mmri.org) |

**List of external facilities and personnel** (cont’d)

| **NINDS Data Safety and Monitoring Board personnel** | **Affiliation** |
| --- | --- |
| Leroy Sharer, MDProfessor of Pathology Consultant | Department of Pathology and Laboratory Medicine  New Jersey Medical School  185 S. Orange Avenue  Newark, NJ 07103  phone: (973) 972-4770; 972-972-7167  fax: (973) 972-5933  email: [Sharer@umdnj.edu](mailto:Sharer@umdnj.edu) |
| Michael Nunn, PhD Program Director  NINDS Staff | Neural Environment  NINDS, NIH  6001 Executive Boulevard  Neuroscience Center, Room 2118  Bethesda, MD 20892-9521  phone: (301) 496-1431  fax: (301) 480-2424  email: mn52@nih.gov |
| Howard E. Gendelman, MD  Professor and Director  Consultant | Center for Neurovirology &  Neurodegenerative Disorders  University of Nebraska Medical Center  985215 Nebraska Medical Center  Omaha, NE 68198-5215  phone: 402-559-8920  fax: 402-559-8922  email: [hegendel@unmc.edu](mailto:hegendel@unmc.edu) |
|  |  |

| **Sponsor’s representative for study conduct** |
| --- |
| PPD Development, LP (“PPD”)  3151 17^th^ Street  Wilmington, North Carolina 28412  phone: (650) 616-5114 |

**List of external facilities and personnel** (cont’d)

| **Central laboratory facility** | **Study drug packaging and shipment** |
| --- | --- |
| ICON Laboratories, Inc.  123 Smith Street  Farmingdale, NY 11735  phone: (631) 777-8833 | Fisher Clinical Services, Inc.  Iron Run Corporate Center  6575 Snowdrift Road  Allentown, PA 18106  phone: (610) 391-0800 |

| **Nerve Conduction Core Laboratory** | **Electronic diary management** |
| --- | --- |
| Dr. Muhammed Al-Lozi  Washington University School of Medicine  Neurology, Box 8111  660 South Euclid  St. Louis, MO 63110  phone: (314) 362-6981  fax: (314) 362-3489  email: allozim@neuro.wustl.edu | invivodata  2100 Wharton Street  Suite 505  Pittsburgh, PA 15203  phone: (412) 390-3000 |

## Signature page for investigators

Compound name: Prosaptide

**Revised protocol and Amendment 2 for the NARC Protocol No. 009** / **Savient Protocol No. C0603/ AACTG Protocol No. A5180**

I agree to conduct this clinical study in accordance with the design and specific provisions of this protocol and its amendment. I agree to report all information or data in accordance with the protocol and, in particular, I agree to report all serious adverse experiences as defined in Section 3.7 of this amended protocol. I also agree to handle all clinical supplies provided by the SPONSOR (Savient) and collect and handle all clinical specimens in accordance with the protocol.

##### __________________________

| Principal/primary investigator  (type or print name) | signature |  | date |
| --- | --- | --- | --- |

Table of contents

Signature page for Savient Pharmaceuticals, Inc. [2](#__RefHeading___Toc74986582)

List of Neurologic AIDS Research (NARC) personnel [3](#__RefHeading___Toc74986583)

Signature page for investigators [7](#__RefHeading___Toc74986584)

List of figures [7](#__RefHeading___Toc74986585)

List of tables [7](#__RefHeading___Toc74986586)

List of abbreviations [7](#__RefHeading___Toc74986587)

Amendment 2 [7](#__RefHeading___Toc74986588)

1-AM2. Amendment 2 overview [7](#__RefHeading___Toc74986589)

1.1-AM2. Rationale [7](#__RefHeading___Toc74986590)

1.2-AM2. Protocol global changes [7](#__RefHeading___Toc74986591)

1.3-AM2. Protocol specific changes [7](#__RefHeading___Toc74986592)

List of external facilities and personnel [7](#__RefHeading___Toc74986593)

Signature page for investigators [7](#__RefHeading___Toc74986594)

List of abbreviations [7](#__RefHeading___Toc74986595)

Protocol synopsis with flow chart [7](#__RefHeading___Toc74986596)

1.3.1-AM2. Introduction/Section 1.2 [7](#__RefHeading___Toc74986597)

1.3.2-AM2. Study Protocol/Section 1.3.4 [7](#__RefHeading___Toc74986598)

1.3.3-AM2. Dose selection/Section 1.3.5 [7](#__RefHeading___Toc74986599)

1.3.4-AM2 Study objectives/Section 2 [7](#__RefHeading___Toc74986600)

1.3.5-AM2. Investigational Plan/Section 3 [7](#__RefHeading___Toc74986601)

1.3.6-AM2. Overall study design/Section 3.1 [7](#__RefHeading___Toc74986602)

1.3.7-AM2. Stratification/Section 3.1.2 [7](#__RefHeading___Toc74986603)

1.3.8-AM2. Inclusion and exclusion criteria/Section 3.2.2. [7](#__RefHeading___Toc74986604)

1.3.9-AM2. Investigational therapy/Section 3.3.1 [7](#__RefHeading___Toc74986605)

1.3.10-AM2 Treatment assignment/Section 3.3.2 [7](#__RefHeading___Toc74986606)

1.3.11-AM2. Concomitant therapy/Section 3.3.4 [7](#__RefHeading___Toc74986607)

1.3.12-AM2. Visit procedures/Section 3.4.2 [7](#__RefHeading___Toc74986608)

1.3.13-AM2. Subject withdrawal from study or discontinuation/Section 3.4.3 [7](#__RefHeading___Toc74986609)

1.3.15 – AM2. Rescue medication assessment/ Section 3.5.5. [7](#__RefHeading___Toc74986610)

1.3.16 -AM2. Laboratory evaluations/Section 3.6.1 [7](#__RefHeading___Toc74986611)

1.3.17 -AM2. Neurological examination/Section 3.6.4 [7](#__RefHeading___Toc74986612)

1.3.18 -AM2. Adverse event definitions/Section 3.7.1 [7](#__RefHeading___Toc74986613)

1.3.19 -AM2. Adverse event reporting/Section 3.7.3 [7](#__RefHeading___Toc74986614)

1.3.20 -AM2. Statistical methods/Section 6.1. [7](#__RefHeading___Toc74986615)

1.3.21 -AM2. Concomitant therapy/Section 6.1.4 [7](#__RefHeading___Toc74986616)

1.3.22 –AM2 Safety evaluation/Section 6.1.6 [7](#__RefHeading___Toc74986617)

1.3.23 –AM2 Periodic safety report/ Section 6.2. [7](#__RefHeading___Toc74986618)

1.3.24-AM2 Sample size and power considerations/Section 6.3 [7](#__RefHeading___Toc74986619)

1.3.25-AM2 Criteria for clinically notable laboratory abnormalities /Section 7.1. [7](#__RefHeading___Toc74986620)

1.3.26 -AM2. Packaging of clinical supplies/Appendix2/Section 2.1.1-AP2 [7](#__RefHeading___Toc74986621)

1.3.27 -AM2. Labeling/Appendix 2/Section 2.1.2-AP2 [7](#__RefHeading___Toc74986622)

1.3.28 –AM2. Changes to the protocol/Appendix 2/Section 2.3.1-AP2 [7](#__RefHeading___Toc74986623)

The revised protocol [7](#__RefHeading___Toc74986624)

Protocol synopsis with flow chart [7](#__RefHeading___Toc74986625)

Reporting of serious adverse events to sponsor’s representative [7](#__RefHeading___Toc74986626)

Contact information of sponsor’s representative for reporting SAEs [7](#__RefHeading___Toc74986627)

Contact information for study conduct: [7](#__RefHeading___Toc74986628)

Corporate Sponsor’s contact information [7](#__RefHeading___Toc74986629)

Introduction [7](#__RefHeading___Toc74986630)

1.1. Prosaptide [7](#__RefHeading___Toc74986631)

1.2. HIV-associated sensory neuropathies [7](#__RefHeading___Toc74986632)

1.3. Justification for present study [7](#__RefHeading___Toc74986633)

1.3.1. Available therapy [7](#__RefHeading___Toc74986634)

1.3.2. Rationale for protocol [7](#__RefHeading___Toc74986635)

1.3.3. Hypotheses [7](#__RefHeading___Toc74986636)

1.3.4. Study protocol [7](#__RefHeading___Toc74986637)

1.3.5. Dose selection [7](#__RefHeading___Toc74986638)

2. Study objectives [7](#__RefHeading___Toc74986639)

2.1. Primary [7](#__RefHeading___Toc74986640)

2.2. Secondary [7](#__RefHeading___Toc74986641)

2.3. Safety [7](#__RefHeading___Toc74986642)

2.4. Endpoint measures [7](#__RefHeading___Toc74986643)

3. Investigational plan [7](#__RefHeading___Toc74986644)

3.1. Overall study design [7](#__RefHeading___Toc74986645)

3.1.2. Stratification [7](#__RefHeading___Toc74986646)

3.1.3. Interim analysis [7](#__RefHeading___Toc74986647)

3.2. Study population [7](#__RefHeading___Toc74986648)

3.2.1. Subject population [7](#__RefHeading___Toc74986649)

3.2.2. Inclusion and exclusion criteria [7](#__RefHeading___Toc74986650)

3.3. Treatments [7](#__RefHeading___Toc74986651)

3.3.1. Investigational therapy, reference therapy and rescue medication [7](#__RefHeading___Toc74986652)

3.3.2. Treatment assignment [7](#__RefHeading___Toc74986653)

3.3.3. Blinding of treatment assignment [7](#__RefHeading___Toc74986654)

3.3.4. Concomitant therapy [7](#__RefHeading___Toc74986655)

3.3.5. Treatment compliance [7](#__RefHeading___Toc74986656)

3.4. Visit schedule and assessments [7](#__RefHeading___Toc74986657)

3.4.1. Visit schedule [7](#__RefHeading___Toc74986658)

3.4.2. Study procedures [7](#__RefHeading___Toc74986659)

3.4.3. Subject withdrawal from study or discontinuation [7](#__RefHeading___Toc74986660)

3.5. Efficacy assessments [7](#__RefHeading___Toc74986661)

3.5.1. Electronic diary (ED) [7](#__RefHeading___Toc74986662)

3.5.2. Gracely pain scale [7](#__RefHeading___Toc74986663)

3.5.3. McGill pain questionnaire [7](#__RefHeading___Toc74986664)

3.5.4. Global physician impression [7](#__RefHeading___Toc74986665)

3.5.5. Rescue medication assessment [7](#__RefHeading___Toc74986666)

3.6. Safety assessments [7](#__RefHeading___Toc74986667)

3.6.1. Laboratory evaluations [7](#__RefHeading___Toc74986668)

3.6.2. Vital signs [7](#__RefHeading___Toc74986669)

3.6.3. Physical examination [7](#__RefHeading___Toc74986670)

3.6.4. Neurological examination [7](#__RefHeading___Toc74986671)

3.6.5. Nerve conduction studies [7](#__RefHeading___Toc74986672)

3.6.6. Karnofsky score [7](#__RefHeading___Toc74986673)

3.7. Adverse event definitions, evaluation criteria and reporting [7](#__RefHeading___Toc74986674)

3.7.1. Adverse event definitions [7](#__RefHeading___Toc74986675)

3.7.2. Adverse event evaluation criteria [7](#__RefHeading___Toc74986676)

3.7.3. Adverse event reporting [7](#__RefHeading___Toc74986677)

3.8. Drug levels and pharmacokinetic assessments [7](#__RefHeading___Toc74986678)

4. Protocol amendments and other changes in study conduct [7](#__RefHeading___Toc74986679)

4.1. Protocol amendments [7](#__RefHeading___Toc74986680)

4.2. Other changes in study conduct [7](#__RefHeading___Toc74986681)

5. Data management [7](#__RefHeading___Toc74986682)

5.1. Data collection [7](#__RefHeading___Toc74986683)

5.2. Database management and quality control [7](#__RefHeading___Toc74986684)

6. Statistical methods [7](#__RefHeading___Toc74986685)

6.1. Statistical methods [7](#__RefHeading___Toc74986686)

6.1.1. Populations [7](#__RefHeading___Toc74986687)

6.1.2. Background and demographic characteristics [7](#__RefHeading___Toc74986688)

6.1.3. Study medication [7](#__RefHeading___Toc74986689)

6.1.4. Concomitant therapy [7](#__RefHeading___Toc74986690)

6.1.5. Efficacy evaluation [7](#__RefHeading___Toc74986691)

6.1.6. Safety evaluation [7](#__RefHeading___Toc74986692)

6.1.7. Statistical methods for pharmacokinetic analysis [7](#__RefHeading___Toc74986693)

6.1.8. Interim analysis [7](#__RefHeading___Toc74986694)

6.2. Safety monitoring and periodic safety reporting [7](#__RefHeading___Toc74986695)

6.3. Sample size and power considerations [7](#__RefHeading___Toc74986696)

7. Notable laboratory value criteria, special methods and scales [7](#__RefHeading___Toc74986697)

7.1. Criteria for clinically notable laboratory abnormalities [7](#__RefHeading___Toc74986698)

7.2. Special methods and scales [7](#__RefHeading___Toc74986699)

8. Reference list [7](#__RefHeading___Toc74986700)

Appendices to the protocol [7](#__RefHeading___Toc74986701)

Appendix 1: Ethics and good clinical practice [7](#__RefHeading___Toc74986702)

1.1-AP1. Institutional review board/Independent ethics committee [7](#__RefHeading___Toc74986703)

1.2-AP1. Informed consent (IC) [7](#__RefHeading___Toc74986704)

1.3-AP1. Compliance with law, audit, and debarment [7](#__RefHeading___Toc74986705)

1.4-AP1. Compliance with financial disclosure requirements [7](#__RefHeading___Toc74986706)

Appendix 2: Procedures and instructions [7](#__RefHeading___Toc74986707)

2.1-AP2. Labeling, packaging, storage, and return of clinical supplies [7](#__RefHeading___Toc74986708)

2.1.1-AP2. Packaging of clinical supplies [7](#__RefHeading___Toc74986709)

2.1.2-AP2. Labeling [7](#__RefHeading___Toc74986710)

2.1.3-AP2. Storage and return of clinical supplies [7](#__RefHeading___Toc74986711)

2.2-AP2. Biological specimens [7](#__RefHeading___Toc74986712)

2.2.1-AP2. Laboratory safety [7](#__RefHeading___Toc74986713)

2.2.2-AP2. Prosaptide antibody levels [7](#__RefHeading___Toc74986714)

2.2.3-AP2. Pharmacokinetic analysis of plasma samples [7](#__RefHeading___Toc74986715)

2.3-AP2. Administrative procedures [7](#__RefHeading___Toc74986716)

2.3.1-AP2. Changes to the protocol [7](#__RefHeading___Toc74986717)

2.3.2-AP2. Monitoring procedures [7](#__RefHeading___Toc74986718)

2.3.3-AP2. Data collection [7](#__RefHeading___Toc74986719)

2.3.4-AP2. Document retention [7](#__RefHeading___Toc74986720)

2.3.5-AP2. Disclosure and confidentiality [7](#__RefHeading___Toc74986721)

2.3.6-AP2. Publication of results [7](#__RefHeading___Toc74986722)

2.3.7-AP2. Discontinuation of study [7](#__RefHeading___Toc74986723)

2.3.8-AP2. Changes in study personnel [7](#__RefHeading___Toc74986724)

Appendix 3: Study drug administration instructions [7](#__RefHeading___Toc74986725)

3.1-AP3. Study drug [7](#__RefHeading___Toc74986726)

3.2-AP3. Drug supplies [7](#__RefHeading___Toc74986727)

3.3-AP3. Study drug storage and return [7](#__RefHeading___Toc74986728)

Appendix 4: Electronic diaries [7](#__RefHeading___Toc74986729)

4.1-AP4. ED software [7](#__RefHeading___Toc74986730)

4.2-AP4. ED software/hardware validation [7](#__RefHeading___Toc74986731)

4.3-AP4. ED technical problems [7](#__RefHeading___Toc74986732)

4.4-AP4. ED procedures for site staff and study subjects [7](#__RefHeading___Toc74986733)

4.4.1-AP4. Site training and qualification [7](#__RefHeading___Toc74986734)

4.4.2-AP4. Subject ED training [7](#__RefHeading___Toc74986735)

4.5-AP4. Subject study compliance review and feedback [7](#__RefHeading___Toc74986736)

Appendix 5: McGill short form pain questionnaire [7](#__RefHeading___Toc74986737)

5.1-AP5. Overall severity of pain types [7](#__RefHeading___Toc74986738)

5.2-AP5. Overall pain intensity – visual analog scale [7](#__RefHeading___Toc74986739)

5.3-AP5. Overall pain intensity [7](#__RefHeading___Toc74986740)

Appendix 6: Laboratory safety tests [7](#__RefHeading___Toc74986741)

6.1-AP6. Laboratory tests [7](#__RefHeading___Toc74986742)

6.2-AP6. Repeat laboratory testing [7](#__RefHeading___Toc74986743)

Appendix 7: Nerve conduction evaluations [7](#__RefHeading___Toc74986744)

7.1-AP7. Core laboratory [7](#__RefHeading___Toc74986745)

7.2-AP7. Nerve conduction study certification [7](#__RefHeading___Toc74986746)

7.3-AP7. Sural nerve conduction study protocol [7](#__RefHeading___Toc74986747)

7.4-AP7. Unilateral peroneal motor nerve conduction study protocol [7](#__RefHeading___Toc74986748)

7.5-AP7. General comments [7](#__RefHeading___Toc74986749)

7.6-AP7. Data flow process [7](#__RefHeading___Toc74986750)

## **List of figures**

Figure 3-1: Schematic illustration of study [7](#__RefHeading___Toc68083132)

## **List of tables**

Table 3-1: Study flow chart (page 1 of 2) [7](#__RefHeading___Toc68083136)

Table 3-1 (cont’d): Study flow chart (page 2 of 2) [7](#__RefHeading___Toc68083137)

Table 3-2: Flow chart for unscheduled and discontinuation visits [7](#__RefHeading___Toc68083138)

Table 3-3: Subject pain intensity measures (13-point Gracely pain intensity scale) [7](#__RefHeading___Toc68083139)

Table 3-4: Global physician impression [7](#__RefHeading___Toc68083140)

Table 3-5: Karnofsky performance score [7](#__RefHeading___Toc68083141)

Table 3-6: Blood sample collection schedule for pharmacokinetic analysis [7](#__RefHeading___Toc68083142)

## List of abbreviations

| **Abbreviation** | **Definition** |
| --- | --- |
| AACTG | Adult AIDS Clinical Trials Group |
| AE | adverse event |
| AIDS | acquired immunodeficiency syndrome |
| ALT | alanine aminotransferase |
| AM2 | Amendment 2 |
| ANC | absolute neutrophil count |
| ANOVA | analysis of variance |
| ANCOVA | analysis of covariance |
| AP | appendix |
| AST | asparate aminotransferase |
| ATN | antiretroviral toxic neuropathy |
| BTGC | Bio-Technology General Corp.(former name of Savient Pharmaceuticals, Inc.) |
| BUN | blood urea nitrogen |
| CBAR | Center of Biostatistics AIDS Research |
| Cl | clearance |
| CMAP | compound muscle action potential |
| CNS | central nervous system |
| CRA | clinical research associate |
| CRF | case report form |
| CRO | clinical research organization |
| dL | deciliter |
| DAIDS | Division of Acquired Immunodeficiency Syndrome |
| DNA | deoxyribonucleic acid |
| DSMB | Data and Safety Monitoring Board |
| DSP | distal sensory polyneuropathy |
| ED | electronic diary |
| EKG | electrocardiogram |
| FDA | Food and Drug Administration |
| GCP | good clinical practice |
| GGT | gamma-glutamyl transaminase |
| HAART | highly active antiretroviral therapy |
| Hgb | hemoglobin |
| HCG | human chorionic gonadotropin |
| HIV | Human immunodeficiency virus |
| HIV-1 | Human immunodeficiency virus type 1 |
| HIV-SN | HIV-associated sensory neuropathy |
| HPLC | high performance liquid chromatography |
| IATA | International Air Transport Association |

List of abbreviations (cont’d)

| **Abbreviation** | **Definition** |
| --- | --- |
| IC | informed consent |
| IEC | independent ethics committee |
| IRB | institutional review board |
| LCD | liquid crystal display |
| IUD | intrauterine device |
| mg | milligram |
| μg | microgram |
| mL | milliliter |
| mm | millimeter |
| μV | microvolt |
| MTD | maximum tolerated dose |
| NARC | Neurologic AIDS Research Consortium |
| NONMEM | NONlinear Mixed Effects Models |
| NCR | non-carbon required |
| NCS | nerve conduction study |
| NIAID | National Institute of Allergy and Infectious Diseases |
| NIH | National Institutes of Health |
| NINDS | National Institute of Neurological Disorders and Stroke |
| NGF | nerve growth factor |
| No. | number |
| OI | opportunistic infection |
| pg | picogram |
| ppi | present pain intensity |
| RhNGF | recombinant human nerve growth factor |
| RNA | ribonucleic acid |
| SAE | serious adverse event |
| Savient | Savient Pharmaceuticals, Inc. |
| SDAC | Statistical Data Analysis Center |
| SF-MPQ | Short form – McGill Pain Questionnaire |
| SGOT | serum glutamic-oxaloacetic transaminase |
| SGPT | serum glutamic-pyruvic transaminase |
| SNAP | sural nerve action potential |
| SOP | standard operating procedure |
| SQ | subcutaneous |
| ULN | upper limit of normal |
| V | volume of distribution |
| VAS | visual analog scale |
| WBC | white blood cell count |

| Amendment 2 |
| --- |

# 1-AM2. Amendment 2 overview

The original version of the protocol was finalized and released for implementation on 15 October 2002. Thereafter, Amendment 1 was finalized and released on 21 March 2003, and the study was launched under Amendment 1. The investigational plan was subsequently revised in order to accommodate an adjustment in the number of investigative centers, to incorporate modifications to the overall protocol design, inclusion/exclusion criteria, and to provide clarification regarding concomitant medication issues. Specifically, this amendment (Amendment 2) will add approximately 15 study sites; stop enrollment in the 2 mg study arm; allow the enrollment of subjects with mild chronic, stable thrombocytopenia (provided there have been no bleeding complications) or with elevated bilirubin due to exposure to atazanavir or other antiretroviral drug therapy; allow some flexibility in the entry criterion for electronic diary compliance; and decrease the length of time subjects need to be on a stable dose of long-acting narcotics prior to study entry. In addition, certain protocol sections are revised to improve clarity.

## 1.1-AM2. Rationale

The protocol is amended to increase the number of investigative sites and to restrict further enrollment in one study arm (2 mg) with the intention of discontinuing it in order to meet the enrollment period for subject recruitment. The 2 mg dose group was chosen because it is believed to be the least likely to show efficacy. It is expected that a sufficient number of subjects will have been treated at 2 mg by the time the amendment is fully implemented to characterize the dose response; thus the scientific integrity of the study will not be compromised. Further, elimination of the lowest dose poses no concern for subject safety.

Access to the primary laboratory records documenting HIV-1 infection at baseline has been more difficult than anticipated. Accordingly, a provision is added to allow confirmatory HIV testing.

Due to the fact that atazanavir has the same effect on bilirubin as indinavir, the exception for the exclusion criteria for elevated bilirubin is expanded to include atazanavir or other drugs in this class.

Several subjects have failed screening due to what is a mild, chronic stable thrombocytopenia. There is no indication from toxicologic or clinical data to suggest that prosaptide has a detrimental effect on platelets, therefore, this criterion is being relaxed to enable broader subject eligibility.

The entry criterion for compliance with electronic dairy completion during the screening phase is being relaxed to allow for extenuating circumstances.

In addition, several administrative changes are incorporated including the corporate sponsor’s name change from Bio-Technology General Corp. (“BTGC”) to Savient Pharmaceuticals, Inc. (“Savient”). Further, the sponsor’s representative for study conduct, PPD Development, LP (“PPD”), is included in this amendment of the protocol. Since the protocol will also be conducted under the aegis of the Adult AIDS Clinical Trials Group (AACTG), information reflecting that designation is also provided in this amendment.

The affected protocol sections are given below by presenting the previous (pre-amendment) version from Amendment 1, followed by the changes as required by Amendment 2.

Following the summary of changes to Amendment 2, the revised protocol is presented in its entirety.

## 1.2-AM2. Protocol global changes

#### Sponsor’s name change

The corporate sponsor’s name is changed from Bio-Technology General Corp. (“BTGC”) to Savient Pharmaceuticals, Inc. (“Savient”). All affected sections of the protocol will adopt the new name with this amendment.

#### Sponsor’s representative for study conduct

PPD, the sponsor’s representative, will be responsible for study conduct, including serious adverse event reporting. All affected protocol sections will refer to the “sponsor’s representative” with this amendment, and when appropriate, the corporate study sponsor (“Savient”) will be referred as “the sponsor.”

#### Sponsor’s contact information

The corporate sponsor’s contact information is updated to reflect an additional physician contact.

#### Treatment

Subjects will continue to be enrolled into the 2 mg per day prosaptide dose arm until all opened drug blocks at sites, consisting of the 5 original dose groups, have been used. Subsequent drug shipments will be sent to sites with randomized drug kits for only the 4, 8, or 16 mg per day prosaptide or placebo study arms. Therefore, references to the 2 mg treatment arm will remain in the protocol.

## 1.3-AM2. Protocol specific changes

All changed text is underlined.

### List of external facilities and personnel

Includes information of sponsor’s representative for study conduct, updated NINDS Safety and Monitoring Board personnel and contact information for the AACTG medical monitor.

### Signature page for investigators

Delete all dedicated spaces for secondary/sub-investigator signatories.

### List of abbreviations

Include “Savient” for Savient Pharmaceuticals, Inc., “AACTG” for Adult AIDS Clinical Trials Group, “DAIDS” for Division of Acquired Immunodeficiency Syndrome, and “NIAID” for National Institute of Allergy and Infectious Diseases.

### Protocol synopsis with flow chart

#### Objectives of Amendment 1 is changed:

The following paragraph has been added to the Objectives:

The study was originally designed as a randomized, double-blind, placebo-controlled study to evaluate 2, 4, 8 and 16 mg per day of prosaptide. An uncertain number of subjects will have been randomized to receive the 2 mg dose before Amendment 2, dropping the 2 mg arm, can be fully implemented. Therefore, the 2 mg dose group will be included in the primary efficacy analysis only if the number enrolled is sufficient to allow an inferential statistical comparison based on a power calculation. Otherwise, it will be used to characterize safety and dose response.

#### Investigational plan of Amendment 1 reads:

Approximately 1200 subjects will be screened in order to enroll 390 subjects (26 per study site) at approximately 15 U.S. centers. Upon consultation with the study sponsor, additional subjects may be recruited at each clinical center. Approximately 12-15 months will be allotted for recruitment from the first to the last subject’s 1^st^ screening visit (Visit 1). The study will be divided into a screening period, baseline period, a 6-week active treatment period, and a two week crossover period. Thirteen weeks will be allotted for each subject from Visit 1 until the completion of the study (Visit 8).

#### Changed to read:

Approximately 1000 subjects will be screened in order to enroll 355 subjects at approximately 30 U.S. centers in order to obtain approximately 70 evaluable subjects for the placebo, 4, 8 and 16 mg prosaptide dose arms. Approximately 18 months will be allotted for recruitment from the first to the last subject’s 1^st^ screening visit (Visit 1). The study will be divided into a screening period, baseline period, a six week active treatment period, and a two week crossover period. Thirteen weeks will be allotted for each subject from Visit 1 until the completion of the study (Visit 8).

#### Treatment of Amendment 1 reads:

Active treatment: Subjects will be randomized to prosaptide 2, 4, 8, or 16 mg or look-alike placebo in a double blind manner.

#### Changed to read:

Active treatment: Subjects will be randomized to prosaptide 2, 4, 8, or 16 mg or look-alike placebo in a double blind manner until all opened drug blocks with the 5 dose groups have been used. Subsequently, subjects will be randomized to one of only three prosaptide doses (4, 8 or 16 mg) or placebo in a double-blind manner.

#### Regimen of Amendment 1 reads:

The treatment plan is as follows:

At Visit 3, subjects will be randomized to one of four prosaptide doses (2, 4, 8 or 16 mg), or look-alike placebo in a double-blind manner.

At Visit 4, subjects will receive one of the 4 dosing levels of drug or placebo (double-blind treatment phase).

At Visit 7, subjects will be “crossed over” with subjects who received active drug receiving placebo and subjects who received placebo receiving 4 mg of prosaptide (double-blind crossover phase).

#### Changed to read:

At Visit 3, subjects will be randomized to one of the prosaptide doses or look-alike placebo in a double-blind manner.

At Visit 4, subjects will receive one of the dosing levels of drug or placebo (double-blind treatment phase).

### At Visit 7, subjects will be “crossed over” with subjects who received active drug receiving placebo and subjects who received placebo receiving 4 mg of prosaptide (double-blind crossover phase). 1.3.1-AM2. Introduction/Section 1.2

#### Amendment 1 reads:

**HIV-associated peripheral neuropathy**

Peripheral neuropathy is the most frequent neurological complication of HIV infection and its treatment with antiretrovirals. Despite recent declines in the incidence rates of HIV-associated dementia and CNS opportunistic infections^12, 13^, sensory neuropathies (HIV-SN) have increased in prevalence to become the most common neurological disorders associated with AIDS. There are two major types of HIV-associated peripheral sensory neuropathy, distal symmetric peripheral neuropathy (DSPN) and antiretroviral toxic neuropathy (ATN), which affect up to 30% of subjects with advanced HIV disease^14, 15^. The risk of development of DSPN is clearly related to CD4 count and plasma HIV RNA levels^16^. The incidence of development of symptomatic DSPN was 52% at 2 years in a cohort of individuals with advanced HIV/AIDS with CD4 count less than 200 (x10^6^ cells/liter)^17^. Antiretroviral toxic neuropathy is associated with dideoxynucleoside analogue usage and shares most of the clinical features of DSPN. The incidence of ATN is related both to the dose and duration of dideoxynucleoside use, with incidence rates ranging from 8% to 26%, depending on whether single or double dideoxynucleoside regimens are used^18^.

The most common symptom of HIV-SN is pain in the feet. The pathology of DSPN involves a length-dependent degeneration of peripheral nerve fibers affecting both small and large nerve fibers. The pathogenesis is unknown, although multifocal macrophage-mediated inflammation with local intra-neural release of proinflammatory cytokines seems the most likely explanation^15^. The pathogenesis of ATN is thought to reflect the selective ability of the dideoxynucleoside analogues to inhibit gamma DNA polymerase and reduce mitochondrial DNA content. Elevated serum lactate levels have been associated with ATN, and mitochondrial DNA levels in subcutaneous fat obtained by punch skin biopsies are also reduced^19^.

#### Changed to read:

**HIV-associated sensory neuropathies**

Sensory neuropathies are the most frequent neurological complication of HIV infection and its treatment with antiretrovirals. Despite recent declines in the incidence rates of HIV-associated dementia and CNS opportunistic infections^12, 13^, sensory neuropathies (HIV-SN) have increased in prevalence to become the most common neurological disorders associated with AIDS. There are two major types of HIV-associated peripheral sensory neuropathy, distal sensory polyneuropathy (DSP) and antiretroviral toxic neuropathy (ATN), which affect up to 30% of subjects with advanced HIV disease^14, 15^. The risk of development of DSP is clearly related to CD4 count and plasma HIV RNA levels^16^. The incidence of development of symptomatic DSP was 52% at 2 years in a cohort of individuals with advanced HIV/AIDS with CD4 count less than 200 (x10^6^ cells/liter)^17^. Antiretroviral toxic neuropathy is associated with dideoxynucleoside analogue usage and shares most of the clinical features of DSP, except that the neuropathy typically develops while taking the “offending” antiretroviral agent. The incidence of ATN is related both to the dose and duration of dideoxynucleoside use, with incidence rates ranging from 8% to 26%, depending on whether single or double dideoxynucleoside regimens are used^18^.

The most common symptom of HIV-SN is pain or uncomfortable sensations in the feet. The pathology of DSP involves a length-dependent degeneration of peripheral nerve fibers affecting both small and large nerve fibers. The pathogenesis is unknown, although multifocal macrophage-mediated inflammation with local intra-neural release of proinflammatory cytokines seems the most likely explanation^15^. The pathogenesis of ATN is thought to reflect the selective ability of the dideoxynucleoside analogues to inhibit gamma DNA polymerase, reduce mitochondrial DNA content and lead to mitochondrial dysfunction. Elevated serum lactate levels have been associated with ATN, and mitochondrial DNA levels in subcutaneous fat obtained by punch skin biopsies are also reduced^19^.

Note: The abbreviation DSPN has been corrected to DSP throughout the amended protocol.

### 1.3.2-AM2. Study Protocol/Section 1.3.4

#### Paragraphs one and two of Amendment 1 read:

This study will be a multicenter, randomized, double-blind, placebo-controlled study of 13 weeks duration to evaluate the effects of 2, 4, 8 and 16 mg per day of prosaptide on neuropathic pain in HIV-associated sensory neuropathies. Subjects will be stratified by presence or absence of a recordable sural nerve action potential (SNAP) amplitude on nerve conduction testing (0-4 μV or >4 μV antidromic).

The study will be a collaborative effort between the Neurologic AIDS Research Consortium (NARC) and Savient Pharmaceuticals, Inc. (formally Bio-Technology General Corp.). It will be funded jointly by the National Institutes of Health (NIH), National Institutes of Neurological Disorders and Stroke (NINDS) via the NARC (Principal investigator, Dr. David Clifford) and by Savient.

#### Changed to read:

This study will be a multicenter, randomized, double-blind, placebo-controlled study of 13 weeks duration to evaluate the effects of various doses of prosaptide on neuropathic pain in HIV-associated sensory neuropathies. The protocol was initially designed to evaluate 2, 4, 8, and 16 mg per day of prosaptide. Due to slow enrollment, and with no unexpected safety concerns in the study to date, Amendment 2 curtails enrollment into the 2 mg dose arm in order to focus on completing enrollment in the 4, 8 and 16 mg arms. Subjects will be stratified by presence or absence of a recordable sural nerve action potential (SNAP) amplitude on nerve conduction testing (0-4 μV or >4 μV antidromic).

The study will be a collaborative effort between the Neurologic AIDS Research Consortium (NARC), Savient Pharmaceuticals, Inc. (formally Bio-Technology General Corp.) and the Adult AIDS Clinical Trials Group. It will be funded jointly by the National Institute of Allergy and Infectious Diseases (NIAID), National Institutes of Neurological Disorders and Stroke (NINDS) via the NARC (Principal investigator, Dr. David Clifford) and by Savient.

### 1.3.3-AM2. Dose selection/Section 1.3.5

#### Paragraph 3 of Amendment 1 reads:

Due to possible differences between HIV neuropathy and diabetic neuropathy as well as the “inverted U” response curve observed in the previous diabetic study, this dose ranging study will utilize doses on either side of 4 mg, in addition to a dose (8 mg) in between 4 and 16 mg. In order to evaluate the “sural nerve hypothesis”, subjects will be stratified based on SNAP amplitude.

#### Changed to read:

Due to possible differences between HIV neuropathy and diabetic neuropathy as well as the “inverted U” response curve observed in the previous diabetic study, this dose ranging study will utilize doses on either side of 4 mg, in addition to a dose (8 mg) in between 4 and 16 mg. It is anticipated that even with curtailed enrollment in the 2 mg arm, there will be a sufficient number of subjects to characterize the dose-response across all dose groups. In order to evaluate the “sural nerve hypothesis”, subjects will be stratified based on SNAP amplitude.

### 1.3.4-AM2 Study objectives/Section 2

#### Changed to read:

(The following paragraph has been added prior to listing the objectives.)

The study was originally designed as a randomized, double-blind, placebo-controlled study to evaluate 2, 4, 8 and 16 mg per day of prosaptide. An uncertain number of subjects (estimated at 40-45) will have been randomized to receive the 2 mg dose before the amendment can be fully implemented. Therefore, the 2 mg dose group will be included in the primary efficacy analysis only if the number enrolled is sufficient to allow an inferential statistical comparison based on a power calculation. All dose groups will be used to characterize safety and dose response.

### 1.3.5-AM2. Investigational Plan/Section 3

#### Amendment 1 reads:

Approximately 1200 subjects will be screened in order to randomize 390 subjects into the study, equally balanced between treatment groups. Assuming a 10% drop out rate, it is anticipated that 350 subjects will complete the study.

#### Changed to read:

Approximately 1000 subjects will be screened in order to randomize 355 subjects into the study. Assuming a 10% drop out rate, it is anticipated that 280 subjects will complete the study in the four treatment groups (equally balanced across the four treatment groups: 4, 8, 16 mg prosaptide and placebo), and approximately 40-45 will be enrolled in the 2 mg prosaptide group.

Pending acceptable chronic toxicology data, an open-label study will be introduced that will afford subjects who meet minimal criteria and have successfully completed the double-blind study the opportunity to receive prosaptide for an extended period of time. In addition to allowing patients the possibility of extended benefit from drug treatment, the study will provide some longer-term safety data. The open-label study will be made available to sites actively participating in this current protocol.

### 1.3.6-AM2. Overall study design/Section 3.1

#### Amendment 1, paragraph 2, reads:

Subjects will be washed out of **adjuvant pain medications** including anticonvulsants, antidepressants, topical analgesics and short-acting narcotics. Long acting opioid analgesics (maintained at a constant daily dose throughout the study) will be permitted in up to 20% of screened subjects at each study site. Subjects taking long-acting narcotics must have received a constant daily dose for at least 90 days prior to Visit 1. All subjects will receive a supply of **acetaminophen 500 mg tablets** as **rescue medication**. Subjects who have intolerable pain despite rescue medication are permitted to discontinue form the study at any time.

#### Changed to read:

Subjects will be washed out of **adjuvant pain medications**, including anticonvulsants, antidepressants, topical analgesics and short-acting narcotics. Long-acting opioid analgesics (maintained at a constant daily dose throughout the study) will be permitted in up to 20% of screened subjects at each study site. Subjects taking long-acting narcotics must have received a constant daily dose for at least 30 days prior to Visit 1. All subjects will receive a supply of **acetaminophen 500 mg caplets** as **rescue medication**. Subjects who have intolerable pain despite rescue medication are permitted to discontinue from the study at any time.

Note: All references to acetaminophen 500 mg tablets in subsequent protocol sections will be changed to read: acetaminophen 500 mg caplets.

### 1.3.7-AM2. Stratification/Section 3.1.2

#### First paragraph of Amendment 1 reads:

Study subjects will be stratified into 2 categories by the sural nerve action potentials determined at baseline by standardized antidromic nerve conduction velocity measurements (0-4.0 μV and >4.0 μV).

#### Changed to read:

Study subjects will be stratified into 2 categories by the sural nerve action potentials determined at baseline by standardized antidromic sural nerve action potential amplitude measurements (0-4.0 μV and >4.0 μV).

### 1.3.8-AM2. Inclusion and exclusion criteria/Section 3.2.2.

#### Sub-section titled: Inclusion criteria

#### Criterion 10 Amendment 1 reads:

1. Subject successfully completed ≥80% of electronic diary endpoints between Visit 2 and 3.

#### Changed to read:

1. Subject successfully completed ≥75% of electronic diary endpoints between Visit 2 and 3.

#### Sub-section titled: Laboratory abnormalities

#### Amendment 1 reads:

1. Subject has an absolute neutrophil count (ANC) <750/mm^3^.
2. Subject has hemoglobin <8.0 g/dL for males or <7.5 g/dL for females.
3. Subject has a platelet count <75,000/mm^3^.
4. Subject has a creatinine >1.5 x upper limit of normal (ULN).
5. Subject has AST (SGOT), ALT (SGPT), and alkaline phosphatase >5x ULN.
6. Subject has total bilirubin >1.5 x ULN. Subjects receiving indinavir, are eligible if their total bilirubin is <4 x ULN and their direct bilirubin is less than the ULN.
7. Subject has HgbA_1_C >6.5.
8. Subject has serum B_12_ ≤200 pg/mL.

#### Changed to read:

1. Subject has an absolute neutrophil count (ANC) <750/mm^3^ (<0.75 x 10^9^/L).
2. Subject has hemoglobin <8.0 g/dL for males or <7.5 g/dL for females.
3. Subject has a platelet count <75,000/mm^3^ (<75 x 10^9^/L). A subject with mild, chronic stable thrombocytopenia and no bleeding complications, as determined and documented by the investigator, may be enrolled with a platelet count as low as 50,000/ mm^3^ (50 x 10^9^/L).
4. Subject has a creatinine >1.5 x upper limit of normal (ULN).
5. Subject has AST (SGOT), ALT (SGPT), and alkaline phosphatase >5x ULN.
6. Subject has total bilirubin >1.5 x ULN. Subjects receiving indinavir, atazanavir, or other drugs with the same known effect on bilirubin levels are eligible if their total bilirubin is <5 x ULN. (Note: Savient must be contacted to discuss possible discontinuation of any subject allowed into the study with a drug-induced hyperbilirubinemia whose total bilirubin approaches 7.0 during the study.)
7. Subject has HgbA_1_C >6.5.
8. Subject has serum B_12_ ≤200 pg/mL.

### 1.3.9-AM2. Investigational therapy/Section 3.3.1

#### Treatment plan of Amendment 1 (first two bullets) reads:

The treatment plan is as follows:

- At Visit 3 (see Table 3-1 under Section 3.4, designated as “**R**” column), subjects will be randomized to one of four prosaptide doses (2 mg, 4 mg, 8 mg, or 16 mg), or look-alike placebo in a double-blind manner.
- At Visit 4, subjects will receive one of the 4 dosing levels of drug or placebo (double-blind treatment phase).

#### Changed to read:

The treatment plan is as follows:

- At Visit 3 (see Table 3-1 under Section 3.4, designated as “**R**” column), subjects will be randomized to one of four prosaptide doses (2 mg, 4 mg, 8 mg, or 16 mg), or look-alike placebo in a double-blind manner until all opened drug blocks with the 5 dose groups have been used. Subsequently, subjects will be randomized to one of only three prosaptide doses (4, 8 or 16 mg) or placebo in a double-blind manner (see section 3.3.2).
- At Visit 4, subjects will receive one of the dosing levels of drug or placebo (double-blind treatment phase).

### 1.3.10-AM2 Treatment assignment/Section 3.3.2

#### Amendment 1 reads:

Subjects will be stratified by center and SNAP status and randomized to receive prosaptide 2, 4, 8 or 16 mg or look-alike placebo every 24 hours for 6 weeks. Study kits will be distributed in numerical order, with each subject receiving the next kit for his or her SNAP group available at the site.

#### Changed to read:

Subjects will be stratified by center and SNAP status and randomized to receive prosaptide 2, 4, 8 or 16 mg or look-alike placebo every 24 hours for 6 weeks. All blocks of 5 kits that have had one or more kits assigned must be used until the supply is exhausted in order to maintain the blind. A mechanism for removing the 2 mg kit from a block with five intact, unassigned study kits will be implemented by the Sponsor in a manner that preserves the blind. All subsequent shipments of study drug will consist of four study kits: 4, 8 or 16 mg prosaptide or placebo. Study kits will be distributed in numerical order, with each subject receiving the next kit for his or her SNAP group available at the site.

### 1.3.11-AM2. Concomitant therapy/Section 3.3.4

#### Sub-section titled: Neuroregenerative and neurotoxic agents

#### Amendment 1 reads:

Vitamin B12 supplementation other than a daily multiple vitamin is prohibited during the study.

#### Changed to read:

Vitamin B12 supplementation other than a daily multiple vitamin is prohibited during the study. (Note: subjects taking B12 injections for at least 3 months prior to screening for a documented B12 deficiency may continue receiving their standard regimen).

#### Sub-section titled: Relief analgesics and pain modifiers

#### Amendment 1 reads:

All adjunctive pain medication must be discontinued at Visit 1 (wash out) and are not permitted at any time during the study. Adjunctive pain medications include but are not limited to tricyclic antidepressants (e.g., amitripyline, [Elavil®], doxepin [Sinequan®], imipramine [Tofranil®], nortriptyline, [Aventyl®, Pamelor®], desipramine [Norpramin®]), mexiletine (Mexitil®), phenytoin (Dilantin®), carbamazepine (Tegretol®, Carbatrol®), gabapentin (Neurontin®), and topical preparations, i.e. capsaicin (Dolorac®, Recapsin®, Theragen®, Zostrix®), lidocaine gel (Lidoderm®).

Short acting opiates must be discontinued at Visit 1 (wash out) and are not permitted at any time during the study. Short acting opiates include but are not limited to fentanyl (Actiq®), propoxyphene compounds (Darvocet®, Darvon®, Wygesic®), meperidine (Demerol®), hydromorphone (Dilaudid®), levorphanol (Levo-Dromoran®), hydrocodone compounds (Lortab®, Norco®, Vicodin®, Zydone®), oxymorphone (Numorphan®), oxycodone compounds (Percocet®, Percodan®, Percolan®, Roxicodone™, OxyIR®, OxyFast®), morphine (Roxanol™), and codeine compounds (Tylox®).

Long-acting opiates including oxycodone (OxyContin®), fentanyl transdermal system (Duragesic®), sustained release morphine (Kadian®, Oramorph® SR, MSContin®), and methadone (Dolophine®) are permitted in up to 20% of screened subjects. Additional subjects requiring long-acting opiates at a given site will result in screen failures. All subjects taking long-active opiates must maintain a constant daily dose throughout the study, and for at least 90 days prior to Visit 1. **Subjects requiring modification of the total daily dose of long-acting opiate prior to Visit 3 (randomization) should be discontinued from the study due to a protocol violation**. Subjects requiring modification of the total daily dose of long-acting opiate after Visit 3 (randomization) should continue in the study for inclusion in the intent-to-treat analysis, but will be flagged as a protocol violator and excluded from the per protocol analysis.

Non-prescription (over-the-counter) analgesics including acetaminophen, aspirin, non-steroidal anti-inflammatory agents, and combination agents must be discontinued at Visit 1 (wash out) and are not permitted at anytime during the study, except for protocol-specified rescue medication.

#### Changed to read:

All adjunctive pain medications being used for neuropathic pain must be discontinued at Visit 1 (wash out). Medications that require weaning may be tapered between Visits 1 and 2 at the discretion of the investigator. After washout, no adjunctive pain medication is permitted **at any time** during the study. Adjunctive pain medications include but are not limited to tricyclic antidepressants (e.g., amitriptyline, [Elavil®], doxepin [Sinequan®], imipramine [Tofranil®], nortriptyline, [Aventyl®, Pamelor®], desipramine [Norpramin®]), lamotrigine [Lamictal®], mexiletine (Mexitil®), phenytoin (Dilantin®), carbamazepine (Tegretol®, Carbatrol®), oxcarbazepine [Trileptal®], topiramate [Topamax®], gabapentin (Neurontin®), and topical preparations, i.e. capsaicin (Dolorac®, Recapsin®, Theragen®, Zostrix®), lidocaine gel (Lidoderm®). (Note, potential subjects receiving other classes of antidepressants for psychiatric use, or anticonvulsants for seizure control, rather than as adjunctive pain medication may be enrolled if they have been on a stable dose for over 30 days).

Short acting opiates must be discontinued at Visit 1 (wash out), or weaned between Visits 1 and 2 at the discretion of the investigator. After washout, no short acting opiates are permitted **at any time** during the study. Short acting opiates include but are not limited to fentanyl (Actiq®), propoxyphene compounds (Darvocet®, Darvon®, Wygesic®), meperidine (Demerol®), hydromorphone (Dilaudid®), levorphanol (Levo-Dromoran®), hydrocodone compounds (Lortab®, Norco®, Vicodin®, Zydone®), oxymorphone (Numorphan®), oxycodone compounds (Percocet®, Percodan®, Percolan®, Roxicodone™, OxyIR®, OxyFast®), morphine (Roxanol™), and codeine compounds (Tylox®).

Long-acting opiates including oxycodone (OxyContin®), fentanyl transdermal system (Duragesic®), sustained release morphine (Kadian®, Oramorph® SR, MSContin®), and methadone (Dolophine®) are permitted in up to 20% of screened subjects. Additional subjects requiring long-acting opiates at a given site will result in screen failures. All subjects taking long-acting opiates must maintain a constant daily dose throughout the study, and for at least 30 days prior to Visit 1. **Subjects requiring modification of the total daily dose of long-acting opiate prior to Visit 3 (randomization) should be discontinued from the study due to a protocol violation**. Subjects requiring modification of the total daily dose of long-acting opiate after Visit 3 (randomization) should continue in the study for inclusion in the intent-to-treat analysis, but will be flagged as a protocol violator and excluded from the per protocol analysis.

Non-prescription (over-the-counter) analgesics including acetaminophen, aspirin, non-steroidal anti-inflammatory agents, and combination agents must be discontinued at Visit 1 (wash out) and are not permitted at anytime during the study, with the exception of no more than 72 hours of over the counter non-steroidal anti-inflammatory drugs if prescribed for the treatment of an adverse event (e.g., sprained ankle). **This exception does not apply during baseline (the week before the first dose) or during Week 6 of therapy (the week before Visit 7).** Protocol-specified rescue medication should be used for all other pain relief (e.g., headache, backache, toothache).

### 1.3.12-AM2. Visit procedures/Section 3.4.2

#### Sub-section titled: Visit 1- Initial screen visit (1^st^ screen phase)

#### Second paragraph of Amendment 1 reads:

Subjects failing to meet the inclusion/exclusion criteria at Visit 1 may be **re-screened up to one time** during the enrollment period at the discretion of the investigator. Subjects who are re-screened must sign a new informed consent form and should receive a new subject number. Subjects who have successfully completed Visit 2 and have been **issued an electronic diary but then become ineligible may not be re-screened, and are therefore withdrawn.**

#### Changed to read:

Subjects failing to meet the inclusion/exclusion criteria at Visit 1 may be **re-screened up to one time** during the enrollment period at the discretion of the investigator. (Subjects who failed screening twice earlier due to a criterion that has been revised in this amendment may be re-screened one additional time.) Subjects who are re-screened must sign a new informed consent form and should receive a new subject number. Subjects who have successfully completed Visit 2 and have been **issued an electronic diary but then become ineligible may not be re-screened, and are therefore withdrawn from the study.**

#### Fifth paragraph of Amendment 1 reads:

Documentation of HIV-1 infection determined by any FDA licensed ELISA test kit and confirmed by Western blot at any time prior to study entry is required. HIV-1 culture, HIV-1 antigen, plasma HIV-1 RNA, or a second antibody test by a method other than ELISA is acceptable as an alternative confirmatory test.

#### Changed to read:

Documentation of HIV-1 infection determined by any FDA licensed ELISA test kit and confirmed by Western blot is required. HIV-1 culture, HIV-1 antigen, plasma HIV-1 RNA, or a second antibody test by a method other than ELISA is acceptable as an alternative confirmatory test. Subjects unable to provide the required documentation at Visit 1 may elect to have confirmatory testing performed by the central laboratory. An ELISA and confirmatory Western blot will be performed on the blood sample collected at Visit 1 only for subjects who have a negative HIV-1 RNA result at Visit 3.

#### Sub-section titled: Visit 2-Second screening visit (2^nd^ screen phase)

#### Amendment 1 reads:

Visit 2 should occur **14 ±3** days from the initial screening visit. Visit 2 procedures will include:

- vital signs
- McGill pain questionnaire
- nerve conduction testing
- issue electronic diary
- review AEs
- review concomitant medications
- rescue drug accountability
- dispense rescue pain medication

#### Changed to read:

Visit 2 should occur **14 ±3** days from the initial screening visit. Visit 2 procedures will include:

- vital signs
- McGill pain questionnaire
- issue electronic diary
- review AEs
- review concomitant medications
- rescue drug accountability
- dispense rescue pain medication
- nerve conduction testing – this procedure may be performed anytime during the screening phase after the results of the screening labs confirm subject eligibility. Care should be taken to allow enough time for a re-test, if needed, before the scheduled randomization visit.

#### Sub-section titled: Visit 3-Randomization visit

#### Paragraph 1, Amendment 1 reads:

Visit 3 should occur **14 ±3** days from the **2^nd^** screening visit. Subjects must have successfully completed at least 80% of electronic diary endpoints in order to be randomized. The **baseline** period will begin at Visit 3. Visit 3 procedures will include:

#### Changed to read:

Visit 3 should occur **14 ±3** days from the **2^nd^** screening visit. Subjects must have successfully completed at least 75% of electronic diary endpoints in order to be randomized. The **baseline** period will begin at Visit 3. Visit 3 procedures will include:

#### Sub-section titled: Unscheduled visit

#### Second paragraph of Amendment 1 reads:

Unscheduled visit procedures will include at least:

- vital signs
- review of adverse events
- review concomitant medications

#### Changed to read:

Delete vital signs. (Note, this has also been deleted from the table entitled “Table 3-2: Flow chart for unscheduled and discontinuation visits” in Section 3.4.1.)

#### Sub-section titled: Discontinuation visit

#### First paragraph of Amendment 1 reads:

All subjects discontinuing from the study after **Visit 3** must complete a discontinuation visit (See Section 3.4.3) for safety reasons. Discontinuation visit procedures will include:

- vital signs
- physical examination
- neurologic examination
- neuropathy assessment
- Karnofsky performance score
- McGill pain questionnaire
- global physician impression
- review of adverse events
- review concomitant medications
- 12-lead EKG
- laboratory safety tests
- urinalysis
- serum β-HCG (females of child-bearing potential only)
- HIV-1 RNA
- CD4 cell count
- review electronic diary
- prosaptide antibody
- drug accountability (rescue and study drug)
- collection of electronic diary

#### Changed to read:

All subjects discontinuing from the study after administration of study drug at **Visit 4** must complete a discontinuation visit (See Section 3.4.3.) for safety reasons. Discontinuation visit procedures will include:

(To the above list is added the following procedure:)

- nerve conduction testing (unless Visit 7 was already completed)

(Note, this has also been added to the table entitled “Table 3-2: Flow chart for unscheduled and discontinuation visits” in Section 3.4.1.)

### 1.3.13-AM2. Subject withdrawal from study or discontinuation/Section 3.4.3

#### Sub-section titled: Criteria for treatment discontinuation

#### Criterion 7 in Amendment 1 reads:

7. treatment failure

#### Changed to read:

7. treatment failure (defined as subject request to leave study due to the need for disallowed pain medication)

#### Sub-section titled: Discontinuation

#### Sixth paragraph of Amendment 1 reads:

If a subject discontinues from the study and has an ongoing adverse experience at the time of discontinuation, **the subject must receive follow up for at least 2 weeks to clarify the nature of the adverse experience**. If a clinical visit(s) is (are) necessary, the procedures performed during the follow up visit(s) will be recorded on Unscheduled visit pages of the CRF. If, after a 2-week period, the adverse experience has not resolved, then a plan for further follow up of the subject will be determined by the investigator in consultation with the NARC Protocol Chair and Savient (the sponsor).

#### Changed to read:

If a subject discontinues from the study and has an ongoing adverse experience at the time of discontinuation, **the subject must receive follow up for at least 2 weeks to clarify the nature of the adverse experience**. If, after a 2-week period, the adverse experience has not resolved, then a plan for further follow up of the subject will be determined by the investigator in consultation with the Protocol Chair and Savient (the sponsor).

#### 1.3.14 –AM2. Global physician impression/Section 3.5.4

#### Amendment 1 reads:

The Global Physician impression will be completed by the neurologist evaluating the study subject at **Visits 1, 4, 7** and **8**. Physicians should rate their subject’s neuropathic pain at the time of the visit by choosing one descriptor in each category. Ideally, the same neurologist should perform all of the evaluations for a given subject in the study. Table 3-4 shows global impression scale for the severity measure.

Table 3-4: Global physician impression

| **Global impression** | **Severity** |
| --- | --- |
| 1 | **normal** with no peripheral neuropathy pain |
| 2 | **borderline** peripheral neuropathy pain |
| 3 | **mild** peripheral neuropathy pain |
| 4 | **moderate** peripheral neuropathy pain |
| 5 | **marked** peripheral neuropathy pain |
| 6 | **severe** peripheral neuropathy pain |
| 7 | among the most **extreme** cases of peripheral neuropathy pain |

#### Changed to read:

The Global Physician impression will be completed by the neurologist evaluating the study subject at **Visits 1, 4, 7** and **8**. Physicians should rate their subject’s neuropathic pain at the time of the visit by choosing one descriptor in each category. Ideally, the same neurologist should perform all of the evaluations for a given subject in the study. Table 3-4 shows global impression scale for the severity measure. Use descriptive categories (in italics below) to identify severity.

Table 3-4: Global physician impression

| **Global impression** | **Severity** |
| --- | --- |
| 1 | **normal** with no peripheral neuropathy pain |
| 2 | **borderline** peripheral neuropathy pain  *e.g. intermittent paresthesias or discomfort; no functional limitation in walking; usually no pain modifying therapy* |
| 3 | **mild** peripheral neuropathy pain  *e.g. intermittent or constant neuropathic pain; mild functional limitation in exercise capacity* |
| 4 | **moderate** peripheral neuropathy pain  *e.g. constant discomfort from neuropathic pain; moderate functional limitation, e.g. cannot walk long distance; frequent nocturnal wakening* |
| 5 | **marked** peripheral neuropathy pain  *e.g. constant neuropathic pain; significant limitation of walking (short distances only); may require assistive device* |
| 6 | **severe** peripheral neuropathy pain  *e.g. neuropathic pain severe enough to require long-acting narcotic; requires assistive device - i.e., cane, walker* |
| 7 | among the most **extreme** cases of peripheral neuropathy pain  *e.g. neuropathic pain severe enough to restrict subject to wheelchair* |

### 1.3.15 – AM2. Rescue medication assessment/ Section 3.5.5.

#### Amendment 1 reads:

All subjects will receive acetaminophen 500 mg tablets as rescue medication, which may be taken up to two tablets 3 times per day (every 8 hours) up to the maximum of 100 tablets dispensed per visit. Subjects **should be instructed to take rescue medication only when absolutely necessary**. After Visit 2, rescue medication will be recorded in the electronic diary at the time of use. Further information with regard to rescue medication is given under Section 3.3.1, and Appendix 2.

#### Changed to read:

All subjects will receive acetaminophen 500 mg caplets as rescue medication, which may be taken up to two caplets 3 times per day (every 8 hours) up to the maximum of 100 caplets dispensed per visit (extra caplets are provided in the event that the interval between visits is extended). Subjects **should be instructed to take rescue medication only when absolutely necessary**. After Visit 2, rescue medication will be recorded in the electronic diary at the time of use. Further information with regard to rescue medication is given under Section 3.3.1, and Appendix 2.

### 1.3.16 -AM2. Laboratory evaluations/Section 3.6.1

#### Third paragraph of Amendment 1 reads:

Notable abnormal values will be reported according to Section 7.1 Criteria for clinically notable laboratory abnormalities. Notable abnormalities which are treatment-emergent will be reported separately.

If a subject discontinues from the study after **Visit 3** for any reason, laboratory tests will be performed at the **discontinuation visit**. Any new abnormal laboratory values found at the post-study evaluation must be repeated until an appropriate explanation has been made or until the value have returned to normal.

#### Changed to read:

Notable abnormal values as defined in Section 7.1 (Criteria for clinically notable laboratory abnormalities) will be reported to the investigators by the central laboratory for immediate safety assessment. The purpose of these reports is to ensure investigators are made aware of any potential safety concerns regarding their patients in a timely manner. In addition, all notable abnormalities will be flagged in the study analysis according to Section 6.1.6 (Safety evaluation). Notable abnormalities which are treatment-emergent will be reported separately.

If a subject discontinues from the study for any reason after receiving study drug, laboratory tests will be performed at the **discontinuation visit**. Any new abnormal laboratory values found at the post-study evaluation must be repeated until an appropriate explanation has been made or until the values have returned to normal.

### 1.3.17 -AM2. Neurological examination/Section 3.6.4

#### Second paragraph of Amendment 1 reads:

Information regarding the neurological examination must be present in the source documentation at the study site. Significant findings present at Visit 1 must be included on the Medical History CRF. Significant findings after Visit 1 that meet the definition of an adverse event must be recorded on the Adverse Event page of the CRF.

#### Changed to read:

Information regarding the neurological examination must be present in the source documentation at the study site. Findings present at Visit 1 must be included on the Neurologic Examination CRF. Significant findings after Visit 1 that meet the definition of an adverse event must be recorded on the Adverse Event page of the CRF. Variations in the neurological examination and neuropathy assessment that are consistent with the underlying disease are not to be routinely captured as adverse events since they will be noted on the Neurologic Examination CRF.

### 1.3.18 -AM2. Adverse event definitions/Section 3.7.1

#### Subsection titled Adverse Events

#### Paragraph 2 of Amendment 1 reads:

Abnormal **laboratory values** or test results constitute adverse events only if they induce clinical signs or symptoms or require therapy, and are recorded on the Adverse Events page of the CRF under the signs, symptoms or diagnosis associated with them. In addition, specific laboratory abnormalities may be defined by the investigator as AEs, regardless of the actual laboratory value, and recorded on the AE page of the CRF.

#### Changed to read:

Abnormal **laboratory values** or test results constitute adverse events only if they are associated with or induce clinical signs or symptoms or require therapy, and are recorded on the Adverse Events page of the CRF under the signs, symptoms or diagnosis associated with them.

### 1.3.19 -AM2. Adverse event reporting/Section 3.7.3

#### Fourth paragraph of Amendment 1 reads:

After the telephone report, a SAE report must be faxed to BTGC (sponsor). Information on the SAE report form is required for timely reporting of SAEs to regulatory authorities. The original SAE report should be retained with the subject’s CRF in the study files at each site.

#### Changed to read:

**Instructions for SAE recording and reporting**

The study site will make copies of the blank initial and follow-up SAE report forms as needed. The site will retain originals of all information faxed to PPD in the Investigator Site File. Any Serious Adverse Event (SAE) must be **reported immediately** by telephone to the Medical Affairs/Pharmacovigilance Department at PPD at **800-201-8725**. A written description of any SAE must be sent to PPD by **facsimile** (**888-488-9697**) **within 24 hours** of its occurrence. The site personnel will refer to the protocol for further clarification.

**Note: If there is any question as to whether an event should be reported, call PPD to discuss the event in question at 800-201-8725.**

### 1.3.20 -AM2. Statistical methods/Section 6.1.

#### First two paragraphs of Amendment 1 read:

This is a double-blind, randomized, placebo controlled study with five dose groups (0, 2, 4, 8, 16 mg/per day). The primary efficacy endpoint is the change of the Gracely pain average score during Week 6 from the Week 0 baseline level (Week 0 minus Week 6 score). The primary objective is to select at least one dose group which is significantly better than the placebo with respect to this endpoint. Furthermore, the dose response relationship among these four groups will be examined.

The study will be stratified with respect to SNAP (+ or -). For each stratum, a random treatment allocation rule will be utilized so that the balance among these five dose groups within each center will be maintained during the study.

#### Changed to read:

This is a double-blind, randomized, placebo controlled study with five dose groups (0, 2, 4, 8, 16 mg/per day). The primary efficacy endpoint is the change of the Gracely pain average score during Week 6 from the Week 0 baseline level (Week 0 minus Week 6 score). The primary objective is to select at least one dose group which is significantly better than the placebo with respect to this endpoint. Furthermore, the dose response relationship among these four groups will be examined.

Due to the slow enrollment, the protocol is amended to restrict further enrollment in the lowest dose arm (2 mg). It is expected that a sufficient number of subjects will have been treated at 2 mg before the amendment is fully implemented. An inferential statistical comparison between the 2 mg and placebo groups will still be carried out if there is a sufficient statistical power to detect a clinically meaningful treatment difference. Regardless, all dose groups will be used to characterize safety and dose response.

The study will be stratified with respect to SNAP (+ or -). For each stratum, a random treatment allocation rule will be utilized so that the balance among these five dose groups (four dose groups after the amendment) within each center will be maintained during the study.

Also, the following final paragraph has been added:

Since subjects receiving active treatment during the 42-day double-blind period will receive placebo injections for 2 weeks at visit 7 and those receiving placebo will be crossed over to receive prosaptide 4 mg, within group comparisons between visits 7 and 8 can be performed to examine the treatment effects due to the cross over. Change from visit 7 by dose group for the following parameters will be summarized: Gracely pain scale, McGill pain questionnaire, global physician impression, and safety laboratories.

### 1.3.21 -AM2. Concomitant therapy/Section 6.1.4

#### First paragraph of Amendment 1 reads:

Concomitant medications will be summarized by therapeutic/pharmacological class and treatment group. In addition, they will be listed by treatment group, name of drug, dates administered, total daily dose, and indication.

#### Changed to read:

Revise 2^nd^ sentence by deleting: “total daily dose”

1.3.22 –AM2 Safety evaluation/Section 6.1.6

#### Third paragraph of Amendment 1 reads:

Laboratory data will be summarized by presenting shift tables using extended normal ranges (baseline to most extreme post-baseline value), by presenting summary statistics of raw data and change from baseline values (means, medians, standard deviations, ranges) and by the flagging of notable values in data listings. Two (or more)-way frequency tables of changes in laboratory test results in terms of normal ranges at the follow-up visits versus baseline by treatment group, as well as changes in absolute values from baseline will be examined for treatment differences. Change from baseline of plasma HIV-1 RNA levels by treatment group will be specifically evaluated.

#### Changed to read:

Laboratory data will be summarized by presenting shift tables using extended normal ranges (baseline to most extreme post-baseline value), by presenting summary statistics of raw data and change from baseline values (means, medians, standard deviations, ranges) and by the flagging of notable values (as defined in section 7.1) in data listings. Two (or more)-way frequency tables of changes in laboratory test results in terms of normal ranges at the follow-up visits versus baseline by treatment group, as well as changes in absolute values from baseline will be examined for treatment differences. Change from baseline of plasma HIV-1 RNA levels by treatment group will be specifically evaluated.

### 1.3.23 –AM2 Periodic safety report/ Section 6.2.

#### Amendment 1 reads:

A periodic safety report will be provided to the Data Safety Monitoring Board once every 6 months without breaking the blind. The report will include summary tables of the safety parameters listed in Section 6.1.6. If there are any safety concerns, the committee will request the trial statistician to perform additional analyses by treatment.

#### Changed to read:

Section title changed to “Safety monitoring and periodic safety reporting”

A periodic safety report will be provided to the Data Safety Monitoring Board and external personnel once every 6 months without breaking the blind, as detailed in the Monitoring Plan developed by CBAR. The report will include summary tables of the safety parameters listed in Section 6.1.6. If there are any safety concerns, the committee will request the trial statistician to perform additional analyses by treatment. Further, summary information on Serious Adverse Events will be provided to the DSMB, DAIDS and the study team as they occur.

### 1.3.24-AM2 Sample size and power considerations/Section 6.3

#### Amendment 1 reads:

Based on two studies of neuropathic pain in subjects with HIV-SN^20, 21^, the average placebo response in the Gracely pain scale is about 0.17, and the standard deviation of the change in Gracely pain scale is approximately 0.35. Therefore, any treatment differences of less than 0.17 can be considered to be not clinically meaningful. This study is designed to detect a minimum treatment difference of 0.17 between any dose group and placebo. In order to have at least 80% power to detect this difference and a significance level of 0.05 (2-sided), 70 evaluable subjects at the end of the trial in each group are required. With 70 subjects per group, the 95% confidence interval for the changes between any dose group and the placebo covers (d-0.12, d+0.12), where d is the observed difference between the two groups. Allowing for early dropouts, 390 subjects will be enrolled in order to have 350 evaluable in the final analysis.

#### Changed to read:

Based on two studies of neuropathic pain in subjects with HIV-SN^20, 21^, the average placebo response in the Gracely pain scale is about 0.17, and the standard deviation of the change in Gracely pain scale is approximately 0.35. Therefore, any treatment differences of less than 0.17 can be considered to be not clinically meaningful. This study is designed to detect a minimum treatment difference of 0.17 between any dose group and placebo. In order to have at least 80% power to detect this difference and a significance level of 0.05 (2-sided), 70 evaluable subjects at the end of the trial in the 4 mg, 8 mg, 16 mg and placebo groups are required. The power to detect this difference between the 2 mg and placebo groups will also be calculated before the final efficacy analysis. With 70 subjects per group, the 95% confidence interval for the changes between any dose group and the placebo covers (d-0.12, d+0.12), where d is the observed difference between the two groups. Allowing for early dropouts and assuming the enrollment of 45 subjects in the 2 mg group, 355 subjects will need to be enrolled for the final analysis.

### 1.3.25-AM2 Criteria for clinically notable laboratory abnormalities /Section 7.1.

#### Amendment 1 reads:

Safety laboratories for this study will include serum chemistry, hematology and liver function tests. Urinalysis will also be performed along with safety laboratories (see Appendix 6). Due to the advanced state of immunodeficiency and multiple concomitant medications anticipated for subjects in this study, a greater variability in laboratory tests is anticipated than would be seen in a normal adult population. In addition, it is anticipated that most subjects will have multiple other concomitant conditions that may adversely influence laboratory tests. Therefore, **determination of causality for laboratory abnormalities will be particularly difficult and will require active, ongoing clinical correlation on the part of the investigator**.

For the purposes of this study, **clinically notable serum chemistry** abnormalities will include blood urea nitrogen (BUN) >3 times the upper limit of normal (ULN), creatinine >3 times ULN, SGOT or SGPT >5 times ULN. Since values of total bilirubin are influenced by concomitant medications, a total bilirubin of >4 times ULN will be considered clinically notable. All levels of bilirubin >1.5 times ULN will be fractionated.

**Clinically notable hematology** values will include a hemoglobin of <2.5 times normal, a total white blood cell count of 2.5 times above or below the normal, and a platelet count of 2.5 times above or below the normal.

**Clinically notable urinalysis** values include dipstick positivity of greater than 1+ on any parameter.

#### Changed to read:

Safety laboratories for this study will include serum chemistry, hematology and liver function tests. Urinalysis will also be performed along with safety laboratories (see Appendix 6). Due to the advanced state of immunodeficiency and multiple concomitant medications anticipated for subjects in this study, a greater variability in laboratory tests is anticipated than would be seen in a normal adult population. In addition, it is anticipated that most subjects will have multiple other concomitant conditions that may adversely influence laboratory tests. Therefore, **determination of causality for laboratory abnormalities will be particularly difficult and will require active, ongoing clinical correlation on the part of the investigator**. The central laboratory will report the predetermined clinically notable laboratory results as specified below to the investigative site immediately. The investigator is responsible for evaluating the laboratory abnormality as a potential adverse event, and for determining required follow-up. Source records should clearly document all follow-up performed.

For the purposes of this study, **clinically notable serum chemistry** abnormalities will include blood urea nitrogen (BUN) >3 times the upper limit of normal (ULN), creatinine >3 times ULN, SGOT or SGPT >5 times ULN. Since values of total bilirubin are influenced by concomitant medications, a total bilirubin of >5 times ULN will be considered clinically notable.

**Clinically notable hematology** values will include a hemoglobin of <6.9 g/dL, a total white blood cell count of <2 x 10^9^/L or ≥ 27.75 x 10^9^/L, and a platelet count of <50 x 10^9^/L or ≥1000 x 10^9^/L.

### 1.3.26 -AM2. Packaging of clinical supplies/Appendix2/Section 2.1.1-AP2

#### Paragraph one of Amendment 1 reads:

Clinical supplies for 855 subjects (randomization numbers 90001-90885) will be provided. One or more blocks of five subjects will initially be sent to each individual center in validated, insulated containers with cold packs. Additional supplies will be packaged and held by Fisher Clinical Services under frozen conditions. Each subject will be assigned a kit containing eight cartons of study medication. There will be nine single use vials per carton.

#### Changed to read:

Clinical supplies for 855 subjects (randomization numbers 90001-90885) will be provided. One or more blocks of five subjects will initially be sent to each individual center in validated, insulated containers with cold packs. Additional supplies will be packaged and held by Fisher Clinical Services under frozen conditions. Fisher will remove the 2 mg kit from the remaining unshipped blocks, so that each block will contain only four kits (4, 8 or 16 mg prosaptide, and placebo). Each subject will be assigned a kit containing eight cartons of study medication. There will be nine single use vials per carton.

### 1.3.27 -AM2. Labeling/Appendix 2/Section 2.1.2-AP2

#### Third paragraph of Amendment 1 reads:

A one-part label will be affixed to each carton containing 9 vials. The label will contain the following information:

- protocol number
- randomization number
- week to be used
- contents
- BTGC identification (name and address)
- caution statement required by US federal law
- administration instructions
- storage instructions

#### Changed to read:

A one-part label will be affixed to each carton containing 9 vials. The label will contain the following information:

- protocol number
- randomization number
- week to be used
- contents
- BTGC identification (name and address)

Note: The sponsor’s former name was Bio-Technology General Corp., which included an abbreviated designation, “BTGC.” Therefore, the labels on the study medication refer to the sponsor as “BTGC.”

- caution statement required by US federal law
- administration instructions
- storage instructions

### 1.3.28 –AM2. Changes to the protocol/Appendix 2/Section 2.3.1-AP2

#### Second paragraph of Amendment 1 reads:

Amendments affecting only administrative aspects of the study do NOT require formal protocol amendments or IRB/IEC approval but the IRB/IEC of each center must be kept informed of such administrative changes. Examples of administrative changes not requiring formal protocol amendments and IRB/IEC approval that can be treated as administrative amendments include:

- changes in the staff used to monitor trials (i.e. BTGC staff versus a CRO)
- minor changes in the packaging or labeling of study drug.

#### Changed to read:

Amendments affecting only administrative aspects of the study do NOT require formal protocol amendments or IRB/IEC approval but the IRB/IEC of each center must be kept informed of such administrative changes. Examples of administrative changes that may not require formal protocol amendments and IRB/IEC approval, that may be or may not be treated as administrative amendments include:

- changes in the staff used to monitor trials (i.e. Savient staff versus a CRO)
- minor changes in the packaging or labeling of study drug.

| The revised protocol |
| --- |

## Protocol synopsis with flow chart

| **Name of product :** | Prosaptide^TM^ (commonly referred as prosaptide) |
| --- | --- |
| **Drug substance:** | Prosaptide is a 14 amino acid polypeptide with the following sequence:  H-Thr-D-Ala-Leu-Ile-Asp-Asn-Asn-Ala-Thr-Glu-Glu-Ile-Leu-Tyr-OH |
| **Protocol numbers:** | NARC Protocol No. 009/Savient Protocol No. C0603/AACTG Protocol No. A5180 |
| **Study title:** | A randomized, double-blind, placebo-controlled, multicenter, dose ranging study to evaluate the efficacy and safety of prosaptide over 6 weeks of treatment for the relief of neuropathic pain associated with HIV-1 |
| **Phase:** | 2 |
| **Objectives:** | **Primary:**   - to determine the efficacy of 2, 4, 8, and 16 mg per day of prosaptide compared to placebo during Week 6 of treatment in subjects with painful HIV-associated sensory neuropathy (DSP or ATN)   **Secondary:**   - to determine the efficacy of 2, 4, 8, and 16 mg per day of prosaptide compared to placebo over 6 weeks of treatment (Week 1 through Week 6) - to determine the efficacy of 2, 4, 8 and 16 mg per day of prosaptide compared to placebo during Week 1 of treatment - to determine the number of subjects who were treatment successes during Week 6 of treatment in the 2, 4, 8, and 16 mg prosaptide groups compared to placebo - to determine the pharmacokinetic profile of prosaptide within this subject population   The study was originally designed as a randomized, double-blind, placebo-controlled study to evaluate 2, 4, 8 and 16 mg per day of prosaptide. An uncertain number of subjects will have been randomized to receive the 2 mg dose before Amendment 2, dropping the 2 mg arm, can be fully implemented. Therefore, the 2 mg dose group will be included in the primary efficacy analysis only if the number enrolled is sufficient to allow an inferential statistical comparison based on a power calculation. Otherwise, it will be used to characterize safety and dose response. |
| **Study design:** | Randomized, double-blind, placebo-controlled |

**Protocol synopsis with flow chart** (cont’d)

| **Investigational plan:** | Approximately 1000 subjects will be screened in order to enroll 355 subjects at approximately 30 U.S. centers in order to obtain approximately 70 evaluable subjects for the placebo, 4, 8 and 16 mg prosaptide dose arms. Approximately 18 months will be allotted for recruitment from the first to the last subject’s 1^st^ screening visit (Visit 1). The study will be divided into a screening period, baseline period, a six week active treatment period, and a two week crossover period. Thirteen weeks will be allotted for each subject from Visit 1 until the completion of the study (Visit 8). |
| --- | --- |
| **Treatment:** | Active treatment: Subjects will be randomized to prosaptide 2, 4, 8, or 16 mg or look-alike placebo in a double blind manner until all opened drug blocks with the 5 dose groups have been used. Subsequently, subjects will be randomized to one of only three prosaptide doses (4, 8 or 16 mg) or placebo in a double-blind manner. |
| **Regimen:** | The treatment plan is as follows:  At Visit 3, subjects will be randomized to one of the prosaptide doses), or look-alike placebo in a double-blind manner.  At Visit 4, subjects will receive one of the dosing levels of drug or placebo (double-blind treatment phase).  At Visit 7, subjects will be “crossed over” with subjects who received active drug receiving placebo and subjects who received placebo receiving 4 mg of prosaptide (double-blind crossover phase). |
| **Investigational drug:** | Prosaptide is supplied for human use as a clear, colorless, odorless sterile solution in phosphate buffer containing 3% mannitol with a pH of 6.8. All dosages are packaged in 2 mL sterile glass vials with Teflon-faced stoppers and flip-off caps. |
| **Reference therapy:** | Look-alike placebo will be supplied as sterile phosphate buffered saline with 3% mannitol, pH 6.8. |

**Protocol synopsis with flow chart (cont’d)**

| **Efficacy criteria:** | **Primary** efficacy endpoint:   - difference between the weekly average of evaluable daily Gracely pain scale during Week 0 baseline period and the weekly average during Week 6 (or the last week before discontinuation of treatment) of the active treatment period   **Secondary** efficacy endpoints:   - difference between the weekly average of daily Gracely pain scale during Week 0 baseline period and the weekly average over the 6-week active treatment period (Week 1 through Week 6) - difference between the weekly average of daily Gracely pain scale during Week 0 baseline period and the weekly average during Week 1 of the active treatment period - number of subjects who were treatment successes at each week of the active treatment period (defined as ≥0.35 units of pain improvement from baseline on the Gracely scale)   **Exploratory** efficacy endpoints:   - comparison of the duration of treatment effect in the 2-week placebo crossover period (Week 7 through Week 8) among the treatment arms in subjects who were treatment successes - comparison between the difference in number of doses of rescue medication used during the Week 0 baseline period and number used during each week of the active treatment period between 2, 4, 8, and 16 mg prosaptide and placebo - comparison between McGill pain questionnaire differences from the Visit 4 baseline measurement and Week 7 measurements between 2, 4, 8, and 16 mg prosaptide and placebo - comparison between global physician impression differences from the Visit 4 baseline measurement and Week 7 measurements between 2, 4, 8, and 16 mg prosaptide and placebo |
| --- | --- |

**Protocol synopsis with flow chart** (cont’d)

| **Safety criteria:** | - vital signs - physical examination - adverse events - neurological examination - laboratory safety tests - HIV-1 RNA - EKG (12-lead) - Karnofsky performance score - nerve conduction studies - prosaptide serum antibodies |
| --- | --- |
| **Statistical methods:** | The primary analysis will be an intent-to-treat analysis. The standard 95% confidence intervals for treatment differences between each dose group and placebo will be constructed for the primary efficacy endpoint. If the safety profiles for the dose groups are similar, the recommended dose will be the one with the best efficacy (largest lower bound of the confidence interval). Similar analyses will be repeated for the secondary efficacy endpoints. In addition, an analysis of covariance which adjusts for the differences in baseline Gracely pain levels for the primary efficacy endpoint will be conducted.  A single interim futility analysis will be conducted after the first 200 subjects have completed the 6-week double-blind treatment period. Specifically, an evaluation of the primary efficacy endpoint between each treatment arm and placebo will be assessed. Treatment arms determined to have minimal possibility of efficacy in a futility analysis will be dropped from the study. In addition, an estimate of variance of the primary efficacy endpoint will be performed, and an evaluation of effect of SNAP will be assessed. Subjects will be added if required to a maximum of 500 randomized subjects. |

**Flow chart for study schedule (page 1 of 2)**

| **Evaluation** | **1^st^ screen phase** | **2^nd^  screen phase** | **R** | **study drug start** | **2 weeks on drug** | **4 weeks on drug** | **crossover** | **final**  **visit** |
| --- | --- | --- | --- | --- | --- | --- | --- | --- |
| **Visit** | **1** | **2** | **3** | **4** | **5** | **6** | **7** | **8** |
| **Week** | **- 4** | **- 2** | **0** | **1** | **3** | **5** | **7** | **9** |
| Informed consent | X |  |  |  |  |  |  |  |
| Status of HIV | X |  |  |  |  |  |  |  |
| Medical/medication history | X |  |  |  |  |  |  |  |
| Vital signs | X | X | X | X | X | X | X | X |
| Physical exam | X |  |  | X |  |  | X | X |
| Neurologic exam | X |  |  | X |  |  | X | X |
| Neuropathy assessment | X |  |  | X |  |  | X | X |
| Karnofsky performance score | X |  |  |  |  |  | X |  |
| Gracely pain score (in clinic) | X |  |  |  |  |  |  |  |
| Issue electronic diary |  | X |  |  |  |  |  |  |
| Review electronic diary**^†^** |  |  | X | X | X | X | X | X |
| Assign randomization number |  |  | X |  |  |  |  |  |
| 12-lead EKG | X |  |  |  |  |  | X |  |
| McGill pain questionnaire |  | X | X | X | X | X | X | X |
| Global physician impression | X |  |  | X |  |  | X | X |
| Safety laboratories | X |  | X |  | X | X | X | X |
| HgbA_1_C | X |  |  |  |  |  |  |  |
| B_12_ level | X |  |  |  |  |  |  |  |
| Urinalysis | X |  | X |  | X | X | X | X |
| Serum β-HCG^‡^ | X |  | X |  |  |  |  | X |
| HIV-1 RNA |  |  | X |  |  |  | X |  |
| Nerve conduction testing |  | X |  |  |  |  | X |  |
| Initiate pain medication washout | X |  |  |  |  |  |  |  |
| Dispense rescue medication | X | X | X | X | X | X | X |  |
| Key: **R= randomize;** crossover=end of treatment phase  ^†^Gracely pain score, rescue medication use, study drug use  ^‡^women of child bearing potential only | | | | | | | | |

**Flow chart for study schedule** (page 2 of 2)

| **Evaluation** | **1^st^ screen phase** | **2^nd^ screen phase** | **R** | **study drug start** | **2 weeks on drug** | **4 weeks on drug** | **crossover** | **final**  **visit** |
| --- | --- | --- | --- | --- | --- | --- | --- | --- |
| **Visit** | **1** | **2** | **3** | **4** | **5** | **6** | **7** | **8** |
| **Week** | **- 4** | **- 2** | **0** | **1** | **3** | **5** | **7** | **9** |
| Administer study drug in clinic |  |  |  | X |  | X**^§^** | X**^§^** |  |
| Instructions on self-injection |  |  |  | X |  |  |  |  |
| Dispense study drug |  |  |  | X | X | X | X |  |
| CD4 cell count |  |  | X |  |  |  | X |  |
| Prosaptide antibody |  |  |  |  |  |  | X |  |
| PK blood sampling**^§^** |  |  |  | X |  | X | X |  |
| Review of AEs |  | X | X | X | X | X | X | X |
| Review concomitant medications |  | X | X | X | X | X | X | X |
| Rescue drug accountability |  | X | X | X | X | X | X | X |
| Study drug accountability |  |  |  |  | X | X | X | X |
| Collection of electronic diary |  |  |  |  |  |  |  | X |
| Key: **R= randomize;** crossover=end of treatment phase  **^§^**designated PK centers only | | | | | | | | |

## Reporting of serious adverse events to sponsor’s representative

ANY SERIOUS ADVERSE EVENT, OR DEATH DUE TO ANY CAUSE, WHICH OCCURS TO ANY SUBJECT ENTERED INTO THE STUDY OR WITHIN 30 DAYS AFTER CESSATION OF TREATMENT, WHETHER OR NOT RELATED TO THE INVESTIGATIONAL PRODUCT, MUST BE REPORTED WITHIN 24 HOURS TO ONE OF THE INDIVIDUALS LISTED BELOW. ALL SUBJECTS WITH SERIOUS ADVERSE EVENTS MUST BE FOLLOWED FOR OUTCOME.

### Contact information of sponsor’s representative for reporting SAEs

**Instructions for SAE recording and reporting**

The study site will make copies of the blank initial and follow-up SAE report forms as needed. The site will retain originals of all information faxed to PPD Development in the Investigator Site File. Any Serious Adverse Event (SAE) must be **reported immediately** by telephone to the Medical Affairs/Pharmacovigilance Department at PPD Development at **800-201-8725**. A written description of any SAE must be sent to PPD Development by **facsimile** (**888-488-9697**) **within 24 hours** of its occurrence. The site personnel will refer to the protocol for further clarification.

**Note: If there is any question as to whether an event should be reported, call PPD to discuss the event in question at 800-201-8725.**

### Contact information for study conduct:

**Edward J. Rozhon, PhD**

Associate Director, Development Planning and Project Management

PPD Development

1111 Bayhill Drive, Suite 125

San Bruno, CA 94066

office: (650) 616-5114

mobile: (650) 302-3023

fax: (650) 616-5200

Email: [edward.rozhon@sanbruno.ppdi.com](mailto:edward.rozhon@sanbruno.ppdi.com)

### Corporate Sponsor’s contact information

**Claudia D. Rehrig, MS**

Manager, Development and Medical Affairs

Savient Pharmaceuticals, Inc.

One Tower Center Boulevard (14^th^ floor)

East Brunswick, NJ 08816

office: (732) 565-4738

fax: (732) 418-9702

email: crehrig@savientpharma.com

**Judith Cohn, MD, PhD**

Senior Director, Development and Medical Affairs

Savient Pharmaceuticals, Inc.

One Tower Center Boulevard (14^th^ floor)

East Brunswick, NJ 08816

office: (732) 565-4754

fax: (732) 418-9702

email: jcohn@savientpharma.com

**Zeb Horowitz, MD**

Senior Vice President, Chief Medical Officer

Savient Pharmaceuticals, Inc.

One Tower Center Boulevard (14^th^ floor)

East Brunswick, NJ 08816

office: (732) 565-4750

fax: (732) 418-8721

email: zhorowitz@savientpharma.com

# Introduction

## Prosaptide

Saposins are a group of small glycoproteins that activate lysosomal hydrolysis of a variety of sphingolipids and are mutated in saposin-deficient human storage diseases. Prosaposin, the protein precursor of saposin A, B, C, and D, was identified as a neurotrophic factor. Prosaptide™ (commonly referred to as prosaptide), is a 14-mer peptide, synthesized from the neuroactive region of saposin C^1-5^.

Prosaptide was active in several *in vivo* neuropathic pain models including the streptozotocin diabetic rat, Seltzer rat (partial ligation of sciatic nerve), and TNF-α administration rat model^6-8^. Prosaptide has also demonstrated a beneficial effect in animal models of neuropathy, including both type 1 (streptozotocin) and type 2 (galactose feeding) diabetes, and paclitaxel-induced toxic neuropathy^9-11^.

In phase 1 clinical trials, prosaptide was compared to placebo in both a dose-escalation trial of single administrations in 29 healthy male volunteers, and a multidose trial of daily injections for 12 days in 30 healthy male volunteers. In both studies, subcutaneous injections at doses up to 300 μg/kg of prosaptide were found to be safe in healthy controls. Pharmacokinetic analysis showed that prosaptide was rapidly absorbed, had a short half life, and showed no accumulation after repeated dosing.

A four-week double-blind, randomized, placebo-controlled multicenter phase 2 clinical trial was conducted to study the safety, tolerability, pharmacokinetic profile, and efficacy of prosaptide for the relief of neuropathic pain associated with diabetes mellitus. Following a 7-day single blind placebo lead-in period, 3 dose levels (1, 4, or 16 mg) of prosaptide or placebo were self-administered daily in a double blind fashion for 28 days by subcutaneous injection in type 1 or type 2 diabetic patients with established painful peripheral neuropathy. The efficacy of prosaptide compared with placebo was evaluated using 5 different pain scales, all of which showed statistically significant or near significant neuropathic pain reduction compared to placebo at the 4 mg dose using an intent-to-treat analysis. The primary efficacy endpoint at week 1 was met with the 4-mg dose, using a subject daily diary visual analogue scale and the McGill visual analogue scale. The analgesic effect was demonstrable throughout the study period using all 5 pain scales. The 1 mg and 16 mg doses did not achieve statistical significance compared to placebo. In a post-hoc analysis, subjects with measurable sural nerve amplitudes on nerve conduction testing showed a treatment effect on mean McGill visual analog scale (VAS) scores compared to placebo. Subjects with absent sural nerve action potential (SNAP) amplitude showed a reduced treatment effect compared to those with detectable SNAPs. The distribution of subjects with measurable SNAPs was not balanced across the 3 groups (25% in the 16 mg dose compared to 58%, 43%, and 50% in the placebo, 1 mg, and 4 mg doses, respectively). This suggests that the treatment effect may be attenuated in severe neuropathies, in which sufficient axonal degeneration has occurred to render SNAPs undetectable.

In the phase 2 diabetes trial, safety was measured by physical and neurological exams, vital sign measurements, electrocardiograms, clinical laboratory tests and adverse event monitoring. There were no apparent dose-related changes in nerve conduction or vibration perception testing, hematology, clinical chemistry, or urinalysis, nor did any subjects develop anti-prosaptide antibodies. A similar number of treatment emergent adverse events were seen in all dose groups, including placebo. Pharmacokinetic analyses showed increased blood levels of prosaptide with increasing dose, with no evidence of drug accumulation after 28 days of dosing.

## HIV-associated sensory neuropathies

Sensory neuropathies are the most frequent neurological complication of HIV infection and its treatment with antiretrovirals. Despite recent declines in the incidence rates of HIV-associated dementia and CNS opportunistic infections^12, 13^, sensory neuropathies (HIV-SN) have increased in prevalence to become the most common neurological disorders associated with AIDS. There are two major types of HIV-associated peripheral sensory neuropathy, distal sensory polyneuropathy (DSP) and antiretroviral toxic neuropathy (ATN), which affect up to 30% of subjects with advanced HIV disease^14, 15^. The risk of development of DSP is clearly related to CD4 count and plasma HIV RNA levels^16^. The incidence of development of symptomatic DSP was 52% at 2 years in a cohort of individuals with advanced HIV/AIDS with CD4 count less than 200 (x10^6^ cells/liter)^17^. Antiretroviral toxic neuropathy is associated with dideoxynucleoside analogue usage and shares most of the clinical features of DSP, except that the neuropathy typically develops while taking the “offending” antiretroviral agent. The incidence of ATN is related both to the dose and duration of dideoxynucleoside use, with incidence rates ranging from 8% to 26%, depending on whether single or double dideoxynucleoside regimens are used^18^.

The most common symptom of HIV-SN is pain or uncomfortable sensations in the feet. The pathology of DSP involves a length-dependent degeneration of peripheral nerve fibers affecting both small and large nerve fibers. The pathogenesis is unknown, although multifocal macrophage-mediated inflammation with local intra-neural release of proinflammatory cytokines seems the most likely explanation^15^. The pathogenesis of ATN is thought to reflect the selective ability of the dideoxynucleoside analogues to inhibit gamma DNA polymerase, reduce mitochondrial DNA content and lead to mitochondrial dysfunction. Elevated serum lactate levels have been associated with ATN, and mitochondrial DNA levels in subcutaneous fat obtained by punch skin biopsies are also reduced^19^.

## 1.3. Justification for present study

### Available therapy

To date, there are few effective treatments for HIV-associated peripheral neuropathy, all of which are symptomatic treatments for pain. Management typically involves the initiation and adjustment of pain-modifying therapies, and the adjustment or discontinuation of potentially neurotoxic antiretrovirals (specifically the dideoxynucleoside analogues). Thus, symptomatic HIV-SN may limit the choice of highly active anti-retroviral therapy (HAART) regimens.

In clinical trials, treatment with recombinant human nerve growth factor improved HIV-associated neuropathic pain, assessed both on the Gracely pain scale and global pain assessments, and neurological examination (specifically pin sensitivity)^20^. However, no effect on epidermal nerve fiber densities was determined during an 18 week placebo-controlled trial in a subset of individuals with open label use for up to 70 weeks^17^. In another trial, in HIV-associated subjects with neuropathic pain neither mexiletine nor amitriptyline provided statistically significant relief of neuropathic pain^21^.

### 1.3.2. Rationale for protocol

Although patients with HIV-associated neuropathic pain represent a large and growing subject population, it remains significantly underserved, with no approved therapies and few pharmaceutical clinical trials.

In preclinical animal models, prosaptide has been shown to be efficacious in the treatment of neuropathic pain caused by a variety of different mechanisms. Although damage to the peripheral nervous system occurs via a different mechanism in diabetes compared to HIV, the end result, a neuropathic pain syndrome, is the same. Therefore, evaluation of prosaptide for the treatment of HIV-related neuropathic pain, both DSP and ATN, is reasonable.

### 1.3.3. Hypotheses

- Prosaptide will improve neuropathic pain in HIV-associated sensory neuropathies (DSP or ATN)
- Prosaptide will be safe and well tolerated in subjects with HIV-associated sensory neuropathies (DSP or ATN)

### 1.3.4. Study protocol

This study will be a multicenter, randomized, double-blind, placebo-controlled study of 13 weeks duration to evaluate the effects of various doses of prosaptide on neuropathic pain in HIV-associated sensory neuropathies. The protocol was initially designed to evaluate 2, 4, 8, and 16 mg per day of prosaptide. Due to slow enrollment, and with no unexpected safety concerns in the study to date, Amendment 2 curtails enrollment into the 2 mg dose arm in order to focus on completing enrollment in the 4, 8 and 16 mg arms. Subjects will be stratified by presence or absence of a recordable sural nerve action potential (SNAP) amplitude on nerve conduction testing (0-4 μV or >4 μV antidromic).

The study will be a collaborative effort between the Neurologic AIDS Research Consortium (NARC), Savient Pharmaceuticals, Inc. (formally Bio-Technology General Corp.) and the Adult AIDS Clinical Trials Group. It will be funded jointly by the National Institute of Allergy and Infectious Diseases (NIAID), National Institutes of Neurological Disorders and Stroke (NINDS) via the NARC (Principal investigator, Dr. David Clifford) and by Savient.

The study will be conducted in accordance with the principles set forth under the Declaration of Helsinki (dated: 1989), and the applicable regulatory requirements of Good Clinical Practice (GCP). Details relevant to the applicable standards are given under Appendix 1 (Ethics and good clinical practice) of this protocol. Additional details relevant to study conduct are given under Appendix 2 (Procedures and instructions).

### Dose selection

Despite the short half life of prosaptide, once daily dosing is supported by pre-clinical animal models, including the streptozotocin diabetic rat and the Seltzer rat, suggesting that the duration of pain reduction with prosaptide dosing is >24 hours^6, 7^. It is further supported by efficacy seen in the phase 2 diabetic trial that was dosed once daily.

Although pre-clinical dose ranging studies suggested a linear dose-response pattern for prosaptide, the dose response in the phase 2 diabetic neuropathy trial was complex, suggesting an optimum dose of 4 mg SQ daily with an “inverted U” response curve. One plausible explanation for the unexpected dose-response relationship is the “sural nerve hypothesis.” Namely, subjects with better preserved nerve function appear to have an augmented response to prosaptide.

Due to possible differences between HIV neuropathy and diabetic neuropathy as well as the “inverted U” response curve observed in the previous diabetic study, this dose ranging study will utilize doses on either side of 4 mg, in addition to a dose (8 mg) in between 4 and 16 mg. It is anticipated that even with curtailed enrollment in the 2 mg arm, there will be a sufficient number of subjects to characterize the dose-response across all dose groups. In order to evaluate the “sural nerve hypothesis”, subjects will be stratified based on SNAP amplitude.

# Study objectives

The study was originally designed as a randomized, double-blind, placebo-controlled study to evaluate 2, 4, 8 and 16 mg per day of prosaptide. An uncertain number of subjects (estimated at 40-45) will have been randomized to receive the 2 mg dose before the amendment can be fully implemented. Therefore, the 2 mg dose group will be included in the primary efficacy analysis only if the number enrolled is sufficient to allow an inferential statistical comparison based on a power calculation. All dose groups will be used to characterize safety and dose response.

## 2.1. Primary

- to determine the efficacy of 2, 4, 8, and 16 mg per day of prosaptide compared to placebo during Week 6 of treatment in subjects with painful HIV-associated sensory neuropathy (DSP or ATN)

## Secondary

- to determine the efficacy of 2, 4, 8, and 16 mg per day of prosaptide compared to placebo over 6 weeks of treatment (Week 1 through Week 6)
- to determine the efficacy of 2, 4, 8 and 16 mg per day of prosaptide compared to placebo during Week 1 of treatment
- to determine the number of subjects who were treatment successes during Week 6 of treatment in the 2, 4, 8, and 16 mg prosaptide groups compared to placebo
- to determine the pharmacokinetic profile of prosaptide within this subject population

## Safety

To determine the safety and tolerability of prosaptide 2, 4, 8, and 16 mg per day compared to placebo in subjects with painful HIV-associated neuropathy as demonstrated by the following endpoints:

- vital signs
- physical examination
- adverse events
- neurological examination
- laboratory safety tests
- HIV-1 RNA
- EKG (12-lead)
- Karnofsky performance score
- nerve conduction studies
- prosaptide serum antibodies

## 2.4. Endpoint measures

#### Primary efficacy endpoint

- difference between the weekly average of evaluable daily Gracely pain scale during Week 0 baseline period and the weekly average during Week 6 (or the last week before discontinuation of treatment) of the active treatment period

#### Secondary efficacy endpoints

- difference between the weekly average of daily Gracely pain scale during Week 0 baseline period and the average over the 6-week active treatment period (Week 1 through Week 6)
- difference between the weekly average of daily Gracely pain scale during Week 0 baseline period and the weekly average during Week 1 of the active treatment period
- number of subjects who were treatment successes at each week of the active treatment period (defined as ≥0.35 units of pain improvement from baseline on the Gracely scale)

#### Safety

- comparison of treatment emergent adverse event rates between the 2, 4, 8 and 16 mg prosaptide and placebo groups
- comparison of plasma HIV RNA levels between the 2, 4, 8, and 16 mg prosaptide and placebo groups.

The safety measures will include reported/observed adverse events, vital signs, physical examination, neurological examination, laboratory safety tests, HIV-1 RNA, EKG, Karnofsky performance score, nerve conduction studies and prosaptide serum antibodies.

#### Exploratory measures

- comparison of the duration of treatment effect in the 2-week placebo crossover period (Week 7 through Week 8) among the treatment arms in subjects who were treatment successes
- comparison between the difference in number of doses of rescue medication used during the Week 0 baseline period and number used during each week of the active treatment period between 2, 4, 8, and 16 mg prosaptide and placebo
- comparison between McGill pain questionnaire differences from the Visit 4 baseline measurement and Week 7 measurements between 2, 4, 8, and 16 mg prosaptide and placebo
- comparison between global physician impression differences from the Visit 4 baseline measurement and Week 7 measurements between 2, 4, 8, and 16 mg prosaptide and placebo

# Investigational plan

Approximately 1000 subjects will be screened in order to randomize 355 subjects into the study. Assuming a 10% drop out rate, it is anticipated that 280 subjects will complete the study in the four treatment groups (equally balanced across the four treatment groups: 4, 8, 16 mg prosaptide and placebo), and approximately 40-45 will be enrolled in the 2 mg prosaptide group.

Pending acceptable chronic toxicology data, an open-label study will be introduced that will afford subjects who meet minimal criteria and have successfully completed the study the opportunity to receive prosaptide for an extended period of time. In addition to allowing patients the possibility of extended benefit from drug treatment, the study will provide some longer-term safety data. The open-label study will be made available to sites actively participating in this current protocol.

## 3.1. Overall study design

This will be a randomized, double-blind, placebo-controlled multicenter study design using four dosing levels (2, 4, 8, and 16 mg/day) to evaluate the efficacy and safety of prosaptide for the relief of neuropathic pain associated with HIV-1. An interim analysis will be performed when 200 subjects complete 6 weeks of treatment.

Subjects will be washed out of **adjuvant pain medications** including anticonvulsants, antidepressants, topical analgesics and short-acting narcotics. Long acting opioid analgesics (maintained at a constant daily dose throughout the study) will be permitted in up to 20% of screened subjects at each study site. Subjects taking long-acting narcotics must have received a constant daily dose for at least 30 days prior to Visit 1. All subjects will receive a supply of **acetaminophen 500 mg caplets** as **rescue medication**. Subjects who have intolerable pain despite rescue medication are permitted to discontinue from the study at any time.

The double-blind phase will include a 6 week active treatment phase with subjects receiving one of the 4 dosing levels of prosaptide or placebo as illustrated in Figure 3-1.

Subjects who meet entry criteria, including a pain level of Gracely ≥0.74 log units averaged over the 2-week period between Visit 2 and Visit 3, and have completed written informed consent will be randomized into the study. Subjects randomized after the **two part** screening phase will complete a one week baseline period, then will receive one of four active treatment or placebo for a **42-day** double-blind treatment period. This will be followed by a **2-week** double-blind **crossover period**, during which subjects previously receiving active treatment will receive placebo injections. **The group receiving placebo during the 42-day double-blind treatment period will be crossed over to receive prosaptide 4 mg for 2 weeks,** forming a modified **cross-over** design*.* The purpose of the crossover period is to assess the duration of prosaptide response after discontinuation of treatment. In addition, it increases the number of subjects who receive active treatment, enhancing the ability to assess the magnitude of the placebo response, and the secondary outcome of early pain response. It also allows all subjects the opportunity to receive active therapy at some point during the study. Study visits will occur every 2 weeks during both double-blind periods.

Figure 3-1: Schematic illustration of study

The figure below represents a schematic presentation of the study.

**Double-blind**

**Crossover**

**Baseline**

**Double-blind Treatment**

**Washout**

#### Placebo

**Prosaptide**

# Prosaptide

**4 mg**

**Randomize**

# Placebo

**-4**

**0**

**1**

**7**

**9**

**3**

**5**

**-2**

**-1**

**-3**

**2**

**4**

**6**

**8**

**Week**

**Visit**

**1**

**3**

**4**

**5**

**6**

**7**

**8**

**2**

### 3.1.2. Stratification

Study subjects will be stratified into 2 categories by the sural nerve action potentials determined at baseline by standardized antidromic sural nerve action potential amplitude measurements (0-4.0 μV and >4.0 μV).

A stratified randomization schema will be included to balance the distribution of SNAP positive and SNAP negative subjects at approximately 1:1 to avoid a mal-distribution with an excessive number of SNAP negative subjects who might have reduced treatment response.

### 3.1.3. Interim analysis

An interim futility analysis will be conducted after the first 200 subjects have completed the 6-week double-blind treatment period. Specifically, an evaluation of the primary endpoint, which is the effect of prosaptide on neuropathic pain compared to placebo during Week 6 of treatment, for each treatment arm will be determined. Treatment arms determined to have minimal possibility of efficacy in the futility analysis will be dropped from the study. In addition, an evaluation of variance of the primary endpoint and effect of SNAP will be assessed. Subjects will be added if required to a maximum of 500 randomized subjects.

Subjects **will continue to be randomized** into the study during the interim data analysis, and follow their assigned treatment regimen to completion. If the interim analysis results in dropping of one or more treatment arms, a protocol amendment will be issued for all subjects randomized subsequently to the interim analysis results. The protocol amendment will detail revised study procedures and drug supply.

## 3.2. Study population

### 3.2.1. Subject population

The study population will consist of male and female subjects ≥18 years old with HIV-associated painful sensory neuropathies (either DSP or ATN). The diagnosis of HIV-associated sensory neuropathy will be confirmed by a neurologist, and each subject must report severity of neuropathic pain of **≥0.74** units on the Gracely pain-intensity scale in their electronic diaries averaged over 2 weeks between Visits 2 and 3.

Subjects **failing** to meet inclusion/exclusion criteria at **Visit 1** may be **re-screened** a maximum of **one time** during the study enrollment period. After successful completion of Visit 2, subjects may NOT be re-screened.

### 3.2.2. Inclusion and exclusion criteria

#### Inclusion criteria

1. Subject is a male or female >18 years old.
2. Subject has documented HIV-1 infection.
3. Subject has stable use or nonuse of dideoxynucleoside reverse transcriptase inhibitors for ≥4 months prior to Visit 1.
4. Subject has painful HIV-associated sensory neuropathy (either DSP or ATN), as confirmed by a neurologist.
5. Subject has an average severity of neuropathic pain reported in the electronic diary between Visit 2 and Visit 3 of **≥0.74** units measured with the Gracely pain intensity scale.
6. Subject agrees to the use of pain-modifying agents during the study as specified by the protocol.
7. Subject (either male or female) agrees not to participate in a conception process (i.e. active attempt to become pregnant or to impregnate, sperm donation, *in vitro* fertilization), and use of contraception as specified in the protocol.
8. Subject has a Karnofsky performance score of ≥60.
9. Subject or legal guardian/representative agrees to written informed consent.
10. Subject successfully completed ≥75% of electronic diary endpoints between Visits 2 and 3..

#### Exclusion criteria

Neurologic conditions

1. Subject has any condition other than HIV infection or antiretroviral therapy that in the opinion of the site neurologist confounds the diagnosis of neuropathy.
2. Subject has received insulin or oral hypoglycemic products for treatment of diabetes mellitus ≤30 days from Visit 1. Dietary control for diabetes will be allowed as long as the diabetes is NOT thought by the site neurologist to be a contributory cause of DSP.
3. Subject has a history of documented vitamin B_12_ deficiency (serum B_12_ level <200 pg/mL) with less than 3 months of B_12_ supplementation prior to Visit 1.
4. Subject has hereditary neuropathy.
5. Subject has compression-related neuropathies, i.e. spinal stenosis.
6. Subject has received treatment with any drug other than the dideoxynucleoside analogues that the site neurologist considers to have significantly contributed to the subject’s neuropathy ≤30 days from Visit 1.
7. Subject has a history of any alcohol-related medical complications within 6 months of Visit 1 including, but not limited to, alcohol withdrawal seizures, hallucinosis, delirium tremens, or being in a detoxification program.
8. Subject has received neurotoxic chemotherapeutic agents ≤90 days from Visit 1.
9. Subject has received neuroregenerative agents ≤90 days from Visit 1.
10. Subject has myelopathy that, in the opinion of the site investigator, would interfere with the evaluation of the subject or accurate completion of the electronic diary.

Other conditions

1. Subject has an active AIDS-defining opportunistic infection (OI) or OI-defining condition ≤30 days from Visit 1. Subjects who have no evidence of active disease and are receiving maintenance therapy for AIDS-related OIs will be eligible.
2. Subject has active major disease, both HIV-related and non-HIV-related including, but not limited to, cardiac disease, pulmonary, or hepatorenal, which in the opinion of the investigator might affect the study.
3. Subject is pregnant or breast-feeding.
4. Subject has any currently active malignancy. Malignancies in remission that do not require further treatment or skin cancers including Kaposi’s sarcoma that do not require systemic treatment **are permitted**.
5. Subject has an allergy/sensitivity to prosaptide, acetaminophen, or its formulations.
6. Subject has received any investigational agent(s) that is not FDA-approved or has participated in any interventional research study ≤30 days from Visit 1.
7. Subject is actively using recreational intravenous drugs, crack cocaine, or intranasal/smoked heroin or methamphetamine.

Laboratory abnormalities

1. Subject has an absolute neutrophil count (ANC) <750/mm^3^ (<0.75 x 10^9^/L).
2. Subject has hemoglobin <8.0 g/dL for males or <7.5 g/dL for females.
3. Subject has a platelet count <75,000/mm^3^ (<75 x 10^9^/L). A subject with mild, chronic stable thrombocytopenia and no bleeding complications, as determined and documented by the investigator, may be enrolled with a platelet count as low as 50,000/mm^3^ (50 x 10^9^/L).
4. Subject has a creatinine >1.5 x upper limit of normal (ULN).
5. Subject has AST (SGOT), ALT (SGPT), and alkaline phosphatase >5x ULN.
6. Subject has total bilirubin >1.5 x ULN. Subjects receiving indinavir, atazanavir, or other drugs with the same known effect on bilirubin levels are eligible if their total bilirubin is <5 x ULN. (Note: Savient must be contacted to discuss possible discontinuation of any subject allowed into the study with a drug-induced hyperbilirubinemia whose total bilirubin approaches 7.0 mg/dL during the study.)
7. Subject has HgbA_1_C >6.5.
8. Subject has serum B_12_ ≤200 pg/mL.

## 3.3. Treatments

### 3.3.1. Investigational therapy, reference therapy and rescue medication

#### Treatment plan

The treatment plan is as follows:

- At Visit 3 (see Table 3-1 under Section 3.4, designated as “**R**” column), subjects will be randomized to one of four prosaptide doses (2 mg, 4 mg, 8 mg, or 16 mg), or look-alike placebo in a double-blind manner until all opened drug blocks with the 5 dose groups have been used. Subsequently, subjects will be randomized to one of only three prosaptide doses (4, 8 or 16 mg) or placebo in a double-blind manner (see section 3.3.2).
- At Visit 4, subjects will receive one of the dosing levels of drug or placebo (double-blind treatment phase).
- At Visit 7, subjects will be “**crossed over,**” with subjects who received active drug receiving placebo and subjects who received placebo receiving 4 mg of prosaptide (double-blind crossover phase).

#### Investigational drug

Prosaptide is supplied for human use as a clear, colorless, odorless sterile solution in phosphate buffer containing 3% mannitol with a pH of 6.8. All dosages are packaged in 2 mL sterile glass vials with Teflon-faced stoppers and flip-off caps.

Prosaptide is stable for at least 12 months when stored at -15° C.

See Appendix 2 (Section 2.1-AP2), Labeling, packaging, storage, and return of clinical supplies for details.

#### Reference therapy

Look-alike placebo will be supplied as sterile phosphate buffered saline with 3% mannitol, pH 6.8. Placebo is packed in 2 mL sterile glass vials with Teflon-faced stoppers and flip-off caps.

#### Study drug administration

After the two phase screening procedures (see Table 3-1 under Section 3.4) and the one week baseline period have been completed, the subject will be instructed in self-administration of subcutaneous injections. The **initial dose** of study drug will be **self-administered** by the study **subject** under the **observation of site personnel**. All subjects should be observed for adverse reactions for at least 30 minutes following the initial dose of study drug. All subsequent doses will be administered at 24-hour intervals for a period of 6 weeks. If a dose is **missed by more than 12 hours, it should not be given**. If the subject **misses more than 4 non-consecutive or 3 consecutive doses of study drug in a 2-week interval, the subject should be discontinued from the study due to noncompliance**.

Subjects at designated centers will also have doses administered in clinic at Visits 6 and 7 so that accurately timed samples for PK analysis can be collected. Study drug from the previous supply, not the new box should be administered at these visits. See Section 3.8: Drug levels and pharmacokinetic assessments for details.

See Appendix 3 (Study drug administration instructions) for details of the study drug administration procedure.

#### Rescue medication

Rescue medication, acetaminophen 500 mg caplets, will be supplied in sealed child proof containers at each visit. Subjects may take up to two caplets of rescue medication every 8 hours on an as needed basis. One hundred caplets of rescue medication will be provided at each visit. **All rescue medication use after Visit 2 must be recorded in the electronic diary.**

Further details are given under Appendix 2 of the protocol.

### 3.3.2. Treatment assignment

Subjects will be stratified by center and SNAP status and randomized to receive prosaptide 2, 4, 8 or 16 mg or look-alike placebo every 24 hours for 6 weeks. All blocks of 5 kits that have had one or more kits assigned must be used until the supply is exhausted in order to maintain the blind. A mechanism for removing the 2 mg kit from a block with five intact, unassigned study kits will be implemented by the Sponsor in a manner that preserves the blind. All subsequent shipments of study drug will consist of four study kits: 4, 8 or 16 mg prosaptide or placebo. Study kits will be distributed in numerical order, with each subject receiving the next kit for his or her SNAP group available at the site.

### 3.3.3. Blinding of treatment assignment

To ensure the integrity of the data being collected, this study is conducted under the sponsor’s (Savient) blinding procedures. These blinding procedures are designed to ensure that the subject, the investigators, and the sponsor’s clinical staff responsible for study conduct remain blinded as to treatment assignment until after completion of the study. Therefore, a subject’s treatment will not be unblinded without prior sponsor approval except in the case of an emergency (i.e. for a serious adverse experience). If unblinding of therapy occurs (i.e., accidental unblinding, emergency unblinding for a serious adverse experience), the investigator will promptly document the circumstances and immediately notify the sponsor’s representative.

The study drug will be packaged in subject-specific kits. Each numbered kit will consist of eight weekly cartons containing nine vials of study drug. Each kit label will consist of a two-part tear off label. The tear off portion should be affixed to the label page of the case report form. In addition, a blinded drug disclosure report will be provided for each subject specific kit. A full explanation of study drug packaging is included under Appendix 2 Section 2.1-AP2: Labeling, packaging, storage, and return of clinical supplies. In the event that it becomes necessary to determine a subject’s treatment assignment, the blinded portion of the report will be scratched off to reveal the study drug identity. All unblinded subjects will be discontinued from the study and must have a discontinuation visit performed as soon as possible (See Section 3.4.3, Subject withdrawal from study or discontinuation).

The randomization schedule will be generated by a qualified Savient (the study sponsor) statistician or designee using a validated system that automates the random assignment of treatment groups to randomization numbers. The sponsor’s statistics department will review the randomization scheme and keep it in a locked storage area after approval.

Randomization data are kept strictly confidential, accessible only to authorized persons, until the time of unblinding. At the conclusion of the study, the incidence of any emergency code breaks will be verified after return of all code break reports and unused drug supplies to Savient. Except as part of the pre-specified interim analysis, only when the study has been completed, the data file verified, and the protocol violations determined, will the drug codes be broken and made available for data analysis.

### 3.3.4. Concomitant therapy

#### Prohibited medications

##### Neuroregenerative and neurotoxic agents

Vitamin B12 supplementation other than a daily multiple vitamin is prohibited during the study. (Note: subjects taking B12 injections for at least 3 months prior to screening for a documented B12 deficiency may continue receiving their standard regimen).

Any neuroregenerative agents including carnitine, propionyl-L-carnitine, D-carnitine, levocarnitine (Carnitor®), rhNGF, neurotrophins, i.e., human growth hormone (somatropin, Serostim®), tacrolimus (Prograf®, FK506) or other similar drug other than the study medications are prohibited within 90 days of Visit 1 and during the study.

Chloramphenicol (Chloromycetin®), disulfiram (Antabuse®), ethionamide (Trecator SC®), hydralazine (Apresoline®), metronidazole and nitrofurantoin (Macrodantin®), or treatment with any drug the investigator considers to be neurotoxic other than the dideoxynucleoside analogues are prohibited within 30 days of Visit 1 and during the study.

Any neurotoxic systemic chemotherapeutic agent including, but not limited to, cisplatin (Platinol-AQ®), vincristine (Oncovin®, Vincasar PFS®) and paclitaxel (Taxol®) are prohibited within 90 days of Visit 1 and during the study.

##### Other investigational agents

Any other investigational agents that are not FDA-approved are prohibited within 30 days of Visit 1 and during the study.

##### Relief analgesics and pain modifiers

All adjunctive pain medications being used for neuropathic pain must be discontinued at Visit 1 (wash out). Medications that require weaning may be tapered between Visits 1 and 2 at the discretion of the investigator. After washout, no adjunctive pain medication is permitted at any time during the study. Adjunctive pain medications include but are not limited to tricyclic antidepressants (e.g., amitripyline, [Elavil®], doxepin [Sinequan®], imipramine [Tofranil®], nortriptyline, [Aventyl®, Pamelor®], desipramine [Norpramin®]), lamotrigine [Lamictal®], mexiletine (Mexitil®), phenytoin (Dilantin®), carbamazepine (Tegretol®, Carbatrol®), oxcarbazepine [Trileptal®], topiramate [Topamax®], gabapentin (Neurontin®), and topical preparations, i.e. capsaicin (Dolorac®, Recapsin®, Theragen®, Zostrix®), lidocaine gel (Lidoderm®). (Note, potential subjects receiving other classes of antidepressants for psychiatric use or anticonvulsants for seizure control rather than as adjunctive pain medication may be enrolled if they have been on a stable dose for over 30 days).

Short acting opiates must be discontinued at Visit 1 (wash out), or weaned between Visits 1 and 2 at the discretion of the investigator. After washout, no short acting opiates are permitted **at any time** during the study. Short acting opiates include but are not limited to fentanyl (Actiq®), propoxyphene compounds (Darvocet®, Darvon®, Wygesic®), meperidine (Demerol®), hydromorphone (Dilaudid®), levorphanol (Levo-Dromoran®), hydrocodone compounds (Lortab®, Norco®, Vicodin®, Zydone®), oxymorphone (Numorphan®), oxycodone compounds (Percocet®, Percodan®, Percolan®, Roxicodone™, OxyIR®, OxyFast®), morphine (Roxanol™), and codeine compounds (Tylox®).

Long-acting opiates including oxycodone (OxyContin®), fentanyl transdermal system (Duragesic®), sustained release morphine (Kadian®, Oramorph® SR, MSContin®), and methadone (Dolophine®) are permitted in up to 20% of screened subjects. Additional subjects requiring long-acting opiates at a given site will result in screen failures. All subjects taking long-acting opiates must maintain a constant daily dose throughout the study, and for at least 30 days prior to Visit 1. **Subjects requiring modification of the total daily dose of long-acting opiate prior to Visit 3 (randomization) should be discontinued from the study due to a protocol violation**. Subjects requiring modification of the total daily dose of long-acting opiate after Visit 3 (randomization) should continue in the study for inclusion in the intent-to-treat analysis, but will be flagged as a protocol violator and excluded from the per protocol analysis.

Non-prescription (over-the-counter) analgesics including acetaminophen, aspirin, non-steroidal anti-inflammatory agents, and combination agents must be discontinued at Visit 1 (wash out) and are not permitted at anytime during the study, except for protocol-specified rescue medication, with the exception of no more than 72 hours of over the counter non-steroidal anti-inflammatory drugs if prescribed for the treatment of an adverse event (e.g., sprained ankle). **This exception does not apply during baseline (the week before the first dose) or during Week 6 of therapy (the week before Visit 7).** Protocol-specified rescue medication should be used for all other pain relief (e.g., headache, backache, toothache).

#### Permitted medications

A constant daily dose of aspirin to a maximum of 325 mg per day is permitted for cardiovascular prophylaxis only.

Use of dideoxynucleoside reverse transcriptase inhibitors is permitted during the study. However, subjects are required to take a stable dideoxynucleoside regimen, i.e. to receive/not receive the same dideoxynucleoside regimen ≥4 months prior to Visit 1. Dideoxynucleoside reverse transcriptase inhibitors include, but are not limited to Zerit® (stavudine, d4T), Videx® (didanosine, ddI) and Hivid® (zalcitabine, ddC).

An alteration in dosing frequency while maintaining the same total daily dose of dideoxynucleosides is allowed. Subjects are strongly encouraged to remain on the same dideoxynucleoside therapy throughout the study. Subjects **changing or discontinuing their dideoxynucleoside regimen prior to Visit 3 should be discontinued from the study due to protocol violation.** After Visit 3, subjects discontinuing or changing their dideoxynucleoside regimen will remain on study drug and complete the study for inclusion in the intention-to-treat analysis. However, these subjects will be flagged as protocol violators and excluded from the per protocol analysis.

### Treatment compliance

Records of **study medication** used, **rescue medication** used, and times of administration will be recorded in the subject **electronic diary**. The subject must return all medication vials both used and unused to the study site for drug accountability at each visit. The subject must also return bottles of rescue medication at each visit. PPD’s site monitor will perform per subject drug accountability during site visits and at the completion of the study. If the subject **misses more than 4 nonconsecutive or 3 consecutive doses of study drug in a 2-week interval, the subject should be discontinued from study due to noncompliance**.

## 3.4. Visit schedule and assessments

### 3.4.1. Visit schedule

The study schedule for the active treatment phase assessments and the two phase screening procedures are depicted in the flow chart displayed under Table 3-1:

######

###### Table 3-1: Study flow chart (page 1 of 2)

| **Evaluation** | **1^st^ screen phase** | **2^nd^  screen phase** | **R** | **study drug start** | **2 weeks on drug** | **4 weeks on drug** | **crossover** | **final**  **visit** |
| --- | --- | --- | --- | --- | --- | --- | --- | --- |
| **Visit** | **1** | **2** | **3** | **4** | **5** | **6** | **7** | **8** |
| **Week** | **- 4** | **- 2** | **0** | **1** | **3** | **5** | **7** | **9** |
| Informed consent | X |  |  |  |  |  |  |  |
| Status of HIV | X |  |  |  |  |  |  |  |
| Medical/medication history | X |  |  |  |  |  |  |  |
| Vital signs | X | X | X | X | X | X | X | X |
| Physical exam | X |  |  | X |  |  | X | X |
| Neurologic exam | X |  |  | X |  |  | X | X |
| Neuropathy assessment | X |  |  | X |  |  | X | X |
| Karnofsky performance score | X |  |  |  |  |  | X |  |
| Gracely pain score (in clinic) | X |  |  |  |  |  |  |  |
| Issue electronic diary |  | X |  |  |  |  |  |  |
| Review electronic diary**^†^** |  |  | X | X | X | X | X | X |
| Assign randomization number |  |  | X |  |  |  |  |  |
| 12-lead EKG | X |  |  |  |  |  | X |  |
| McGill pain questionnaire |  | X | X | X | X | X | X | X |
| Global physician impression | X |  |  | X |  |  | X | X |
| Safety laboratories | X |  | X |  | X | X | X | X |
| HgbA_1_C | X |  |  |  |  |  |  |  |
| B_12_ level | X |  |  |  |  |  |  |  |
| Urinalysis | X |  | X |  | X | X | X | X |
| Serum β-HCG^‡^ | X |  | X |  |  |  |  | X |
| HIV-1 RNA |  |  | X |  |  |  | X |  |
| Nerve conduction testing |  | X |  |  |  |  | X |  |
| Initiate pain medication washout | X |  |  |  |  |  |  |  |
| Dispense rescue medication | X | X | X | X | X | X | X |  |
| Key: **R= randomize;** crossover=end of treatment phase  **^†^**Gracely pain score, rescue medication use, study drug use  **^‡^**women of child bearing potential only | | | | | | | | |

######

###### Table 3-1 (cont’d): Study flow chart (page 2 of 2)

| **Evaluation** | **1^st^ screen phase** | **2^nd^ screen phase** | **R** | **study drug start** | **2 weeks on drug** | **4 weeks on drug** | **crossover** | **final**  **visit** |
| --- | --- | --- | --- | --- | --- | --- | --- | --- |
| **Visit** | **1** | **2** | **3** | **4** | **5** | **6** | **7** | **8** |
| **Week** | **- 4** | **- 2** | **0** | **1** | **3** | **5** | **7** | **9** |
| Administer study drug in clinic |  |  |  | X |  | X**^§^** | X**^§^** |  |
| Instructions on self-injection |  |  |  | X |  |  |  |  |
| Dispense study drug |  |  |  | X | X | X | X |  |
| CD4 cell count |  |  | X |  |  |  | X |  |
| Prosaptide antibody |  |  |  |  |  |  | X |  |
| PK blood sampling**^§^** |  |  |  | X |  | X | X |  |
| Review of AEs |  | X | X | X | X | X | X | X |
| Review concomitant medications |  | X | X | X | X | X | X | X |
| Rescue drug accountability |  | X | X | X | X | X | X | X |
| Study drug accountability |  |  |  |  | X | X | X | X |
| Collection of electronic diary |  |  |  |  |  |  |  | X |
| Key: **R= randomize;** crossover=end of treatment phase  **^§^**designated PK centers only | | | | | | | | |

Table 3-2 presents a flow chart for unscheduled and discontinuation visits with the list of respective evaluation tests.

######

###### Table 3-2: Flow chart for unscheduled and discontinuation visits

| **Evaluation** | **Unscheduled** | **Discontinuation** |
| --- | --- | --- |
| Vital signs |  | X |
| Physical exam |  | X |
| Neurologic exam |  | X |
| Karnofsky performance score |  | X |
| Review of AEs | X | X |
| Review concomitant medications | X | X |
| Neuropathy assessment |  | X |
| Nerve conduction testing |  | X |
| 12-lead EKG |  | X |
| Laboratory safety tests |  | X |
| Urinalysis |  | X |
| Serum β-HCG**^†^** |  | X |
| CD4 cell count and percent |  | X |
| Prosaptide antibody |  | X |
| HIV-1 RNA |  | X |
| Review electronic diary |  | X |
| McGill pain questionnaire |  | X |
| Global physician impression |  | X |
| Drug accountability |  | X |
| Collection of electronic diary |  | X |
| **^†^**Females of child bearing potential only | | |

### 3.4.2. Study procedures

#### Visit 1—Initial screen visit (1^st^ screen phase)

Once a candidate for the study has been identified, details of the protocol must be carefully discussed with the candidate, and the candidate will be asked to read and sign the informed consent form approved by the local IRB. The screening process may only begin after informed consent is signed. For further details see Section 1.2-AP1 of Appendix 1.

Subjects failing to meet the inclusion/exclusion criteria at Visit 1 may be **re-screened up to one time** during the enrollment period at the discretion of the investigator. (Subjects who failed screening twice earlier due to a criterion that has been revised in this amendment may be re-screened one additional time.) Subjects who are re-screened must sign a new informed consent form and should receive a new subject number. Subjects who have successfully completed Visit 2 and have been **issued an electronic diary but then become ineligible may not be re-screened, and are therefore withdrawn from the study.**

All women of child bearing potential (defined for this study as sexually mature women who have not reached menopause, or who have not undergone hysterectomy, bilateral oophorectomy, or tubal ligation as documented by subject-reported history) must have a negative serum pregnancy test at both Visit 1 and Visit 3.

All subjects participating in the study must agree to use an appropriate form of contraception during the entire period of the study and for 30 days after stopping study medication. For the purposes of this study, acceptable forms of contraception include: 1) abstinence, 2) condoms (male or female) with a spermicidal agent (3) diaphragm or cervical cap with spermicidal agent, 4) IUD, 5) oral contraceptive, 6) injectable hormonal contraceptive, 7) successful vasectomy with resulting azoospermia or azoospermia for any other reason, and 8) hysterectomy, bilateral oophorectomy, or tubal ligation.

Documentation of HIV-1 infection determined by any FDA licensed ELISA test kit and confirmed by Western blot is required. HIV-1 culture, HIV-1 antigen, plasma HIV-1 RNA, or a second antibody test by a method other than ELISA is acceptable as an alternative confirmatory test. Subjects unable to provide the required documentation at Visit 1 may elect to have confirmatory testing performed by the central laboratory. An ELISA and confirmatory Western blot will be performed on the blood sample collected at Visit 1 only for subjects who have a negative HIV-1 RNA result at Visit 3.

A medical history must be present in source documents. The medical history should include any major and/or chronic HIV-related diagnoses and non-HIV-related diagnoses of major organ systems. All signs, symptoms, HIV-related and AIDS-defining events, and toxicities must be documented at screening.

Documentation of the following on the CRF is required:

- any AIDS-defining diagnoses
- any active diagnoses or HIV-related diagnoses
- any allergies to any medications and their formulations
- any neurologic condition

A complete HIV treatment history of any antiretroviral medication, immune-based therapy, or HIV-related vaccines, including blinded study medications will be recorded. It is important to obtain the initiation dates or dates of any changes in the dideoxynucleoside analogues. Complete treatment history of any prescription medications taken for the treatment or prophylaxis of OIs will be documented. All prescription medications within 90 days of Visit 1, including adjunctive analgesic medication (both for chronic and occasional or as needed use) should be recorded.

Screening neurologic examination and neuropathy assessment, including documentation of the diagnosis of HIV-associated peripheral neuropathy, must be completed by a neurologist. In addition, neuropathy onset associated with dideoxynucleosides use is to be determined by the neurologist.

Other screening procedures will include:

- vital signs
- physical examination
- global physician impression
- Karnofsky performance score
- Gracely pain score (**in clinic**)
- 12-lead EKG
- initiation of wash out for concomitant pain medications
- dispense rescue pain medication

Screening laboratories evaluations will include:

- laboratory safety tests
- urinalysis
- serum ß-HCG (females of child bearing potential only)
- serum B_12_
- HgbA_1_C

#### Visit 2—Second screening visit (2^nd^ screen phase)

Visit 2 should occur **14 ±3** days from the initial screening visit. Visit 2 procedures will include:

- vital signs
- McGill pain questionnaire
- issue electronic diary
- review AEs
- review concomitant medications
- rescue drug accountability
- dispense rescue pain medication
- nerve conduction testing – this procedure may be performed anytime during the screening phase after the results of the screening labs confirm subject eligibility. Care should be taken to allow enough time for a re-test, if needed, before the scheduled randomization visit.

#### Visit 3—Randomization visit

Visit 3 should occur **14 ±3** days from the **2^nd^** screening visit. Subjects must have successfully completed at least 75% of electronic diary endpoints in order to be randomized. The **baseline** period will begin at Visit 3. Visit 3 procedures will include:

- vital signs
- review electronic diary
- evaluation of adverse events
- review of concomitant medications
- McGill pain questionnaire
- rescue drug accountability
- dispense rescue medication
- assignment of randomization number
- safety laboratories
- urinalysis
- HIV-1 RNA
- CD4 cell count
- serum β-HCG (females of child bearing potential only)

#### Visit 4—Study drug initiation visit

Visit 4 should occur **7 ±3** days after Visit 3. The active double-blind treatment period will begin at Visit 4. Visit 4 procedures will include:

- vital signs
- physical examination
- neurologic examination
- neuropathy assessment
- global physician impression
- evaluation of adverse events
- review of concomitant medications
- McGill pain questionnaire
- review of electronic diary
- rescue drug accountability
- dispense rescue medication
- instructions in study drug administration
- administer study drug in clinic
- blood collection for PK analysis (designated PK centers)
- dispense study drug and administration supplies (needles/syringes, alcohol wipes, sharps containers)

Note: Detailed instructions for each subject regarding study drug administration are given under Appendix 3 of the protocol. They include relevant study drug information, description of the actual drug supplies for each visit, syringe use, preparation of dose, steps to be followed for self-injection, and information for drug storage.

#### Visits 5 and 6—Active treatment visits

Visits 5 and 6 should occur **14 ±3** days from the previous study visit. Visits 5 and 6 procedures will include:

- vital signs
- evaluation of adverse events
- safety laboratory tests
- urinalysis
- review of concomitant medications
- review of electronic diary understanding and compliance
- McGill pain questionnaire
- administer study drug in clinic (designated PK centers at Visit 6 only)
- blood collection for PK analysis (selected sites at Visit 6 only)
- rescue drug accountability
- dispense rescue medication
- study drug accountability
- dispense study drug and administration supplies (needles/syringes, alcohol wipes, sharps containers)

#### Visit 7—Crossover visit (end of active treatment phase)

Visits 7 should occur **14 ±3** days from the previous study visit. Visit 7 procedures will include:

- vital signs
- physical examination
- neurologic examination
- neuropathy assessment
- global physician impression
- nerve conduction studies
- Karnofsky performance score
- evaluation of adverse events
- review of concomitant medications
- review of electronic diary understanding and compliance
- McGill pain questionnaire
- safety laboratories
- urinalysis
- 12-lead EKG
- HIV-1 RNA
- CD4 cell count
- administer study drug in clinic (designated PK centers)
- blood collection for PK analysis (selected sites only)
- collect blood samples for prosaptide antibody levels
- rescue drug accountability
- dispense rescue medication
- study drug accountability
- dispense study drug and administration supplies (needles/syringes, alcohol wipes, sharps containers)

#### Visit 8—Final visit

Visits 8 should occur **14 ±3** days from the previous study visit. Visit 8 procedures will include:

- vital signs
- physical examination
- neurologic examination
- neuropathy assessment
- global physician impression
- McGill pain questionnaire
- evaluation of adverse events
- review of concomitant medications
- rescue drug accountability
- study drug accountability
- safety laboratories
- urinalysis
- serum ß-HCG (females of child bearing potential only)
- review electronic diary
- collection of electronic diary

#### Unscheduled visit

Any subject visit to the study site between protocol specified study visits, or for follow up of adverse events after completion of the study is termed an unscheduled visit. Subjects must complete unscheduled visit procedures specified below. Additional study procedures performed at unscheduled visits are at the discretion of the investigator.

Unscheduled visit procedures will include at least:

- review of adverse events
- review concomitant medications

#### Discontinuation visit

All subjects discontinuing from the study after administration of study drug at **Visit 4** must complete a discontinuation visit (See Section 3.4.3) for safety reasons. Discontinuation visit procedures will include:

- vital signs
- physical examination
- neurologic examination
- neuropathy assessment
- nerve conduction testing (unless Visit 7 was already completed)
- Karnofsky performance score
- McGill pain questionnaire
- global physician impression
- review of adverse events
- review concomitant medications
- 12-lead EKG
- laboratory safety tests
- urinalysis
- serum β-HCG (females of child-bearing potential only)
- HIV-1 RNA
- CD4 cell count
- review electronic diary
- prosaptide antibody
- drug accountability (rescue and study drug)
- collection of electronic diary

### 3.4.3. Subject withdrawal from study or discontinuation

It is the goal of the study that all subjects will complete blinded therapy, and will complete all follow up procedures through **Visit 8**. However, it is understood that there may be reasons that subjects may require discontinuation from the study.

#### Criteria for treatment discontinuation

Subjects can be discontinued for any of the following reasons:

1. adverse event
2. consent withdrawn
3. lost to follow up
4. non-compliance (defined by investigator judgment as significant risk of failing to comply with the provisions of the protocol that causes harm to the subject or seriously interferes with the validity of the study results)
5. protocol violation
6. administrative reason (at the discretion of the clinical investigator or sponsor)
7. treatment failure (defined as subject request to leave study due to the need for disallowed pain medication)

#### Discontinuation

Subjects may be discontinued from the study due to the criteria for treatment discontinuation listed above. A subject who discontinues from the study at any time after receiving study drug at **Visit 4** must have a **discontinuation visit** performed. A discontinuation visit should be performed as soon as possible after it has been determined that a subject will actually be discontinued from the study, even if it is before the time of the next regularly scheduled visit. The purpose of the discontinuation visit is to ensure that adequate safety information has been collected for the subject.

All discontinuations due to an adverse event with the outcome of **death** should be reported to the sponsor’s representative (PPD) immediately. Death should NOT be listed as a reason for discontinuation. Instead, the adverse event leading to death should be recorded as the reason for discontinuation.

For those subjects who die, as much information as possible should be completed on the Discontinuation visit pages of the CRF. In addition, a **serious adverse event form** should be completed for the adverse event leading to death. If an autopsy is performed, a copy of the autopsy report must be submitted to Savient (the sponsor).

Any subject **missing** more than **4 non-consecutive or** **3 consecutive doses** of study drug during a 2-week interval should be discontinued due to noncompliance.

If a subject **moves** to an area outside the region of the study center, he or she should be considered to be **lost** to follow up and discontinued from the study.

If a subject discontinues from the study and has an ongoing adverse experience at the time of discontinuation, **the subject must receive follow up for at least 2 weeks to clarify the nature of the adverse experience**. If, after a 2-week period, the adverse experience has not resolved, then a plan for further follow up of the subject will be determined by the investigator in consultation with the Protocol Chair and Savient (the sponsor).

Any subject **discontinuing** or **changing** his or her **dideoxynucleoside regimen prior to Visit 3** should be **discontinued** from study due to protocol violation. In addition, any subject **changing** his or her **long-acting opioid regimen prior to Visit 3** should be **discontinued**. After Visit 3, subjects changing their dideoxynucleoside or long-acting opioid regimen will remain on study drug and complete the study for inclusion in the intention-to-treat analysis. However, these subjects will be flagged as protocol violators and excluded from the per protocol analysis.

## 3.5. Efficacy assessments

Efficacy endpoints will include assessments of neuropathic pain as measured by the Gracely pain scale, McGill pain questionnaire and the Global Physician impression. In addition, use of rescue pain medication will be recorded. All daily assessments will be recorded by the study subject using an **electronic diary**.

### 3.5.1. Electronic diary (ED)

Subjects will be issued an electronic diary and instructed in its use at the second screening phase (see Table 3-1, Visit 2). Data captured on the ED will include the Gracely pain scale, study medication dosing, and rescue medication use. In addition, a **morning** assessment of sleep quality and an **evening** daily pain score will be captured. Data will be uploaded from each ED on a nightly basis to a central database. At each visit, use of the diary should be reviewed with the subject and any questions answered. The subject must return the ED to the study site upon completion of the study. Further details regarding the ED are available in Appendix 4.

### 3.5.2. Gracely pain scale

Subjects will be instructed to choose pain descriptors (see Table 3-3) that describe their current pain intensity from the neuropathy 13-point Gracely pain scale at the time of the random ED prompt. After Visit 2, subjects will be instructed to enter the information into the ED when prompted, approximately 5 times per day. Gracely pain scale measurements performed during **Visit 1** (prior to issuing the electronic diary) will be recorded directly on the CRF by the study subject^22^.

###### Table 3-3: Subject pain intensity measures (13-point Gracely pain intensity scale)

| **Letter** | **Description** | **Scale*** |
| --- | --- | --- |
| A | nothing | 0.00 |
| B | faint | 0.36 |
| C | very weak | 0.40 |
| D | weak | 0.45 |
| E | very mild | 0.59 |
| F | mild | 0.74 |
| G | moderate | 1.09 |
| H | barely strong | 1.10 |
| I | slightly intense | 1.33 |
| J | strong | 1.36 |
| K | intense | 1.54 |
| L | very intense | 1.64 |
| M | extremely intense | 1.77 |
| *log units | | |

### 3.5.3. McGill pain questionnaire

The short form of the McGill Pain Questionnaire (SF-MPQ) will be utilized in this study. It should be completed by the study subject at **Visits 2-8**. The main component of the SF-MPQ consists of 15 descriptors (11 sensory, 4 affective) which are rated on an intensity scale as 0=none, 1=mild, 2=moderate, and 3=severe. Three pain scores will be derived from the sum of the intensity rank values of the words chosen for sensory, affective and total descriptors. The SF-MPQ also includes a present pain intensity (PPI) index and a visual analog scale (VAS). An example of the SF-MPQ is found in Appendix 5^23^.

### Global physician impression

The Global Physician impression will be completed by the neurologist evaluating the study subject at **Visits 1, 4, 7** and **8**. Physicians should rate their subject’s neuropathic pain at the time of the visit by choosing one descriptor in each category. Ideally, the same neurologist should perform all of the evaluations for a given subject in the study. Table 3-4 shows global impression scale for the severity measure. Use descriptive categories (in italics below) to identify severity.

#### Table 3-4: Global physician impression

| **Global impression** | **Severity** |
| --- | --- |
| 1 | **normal** with no peripheral neuropathy pain |
| 2 | **borderline** peripheral neuropathy pain  *e.g. intermittent paresthesias or discomfort; no functional limitation in walking; usually no pain modifying therapy* |
| 3 | **mild** peripheral neuropathy pain  *e.g. intermittent or constant neuropathic pain; mild functional limitation in exercise capacity* |
| 4 | **moderate** peripheral neuropathy pain  *e.g. constant discomfort from neuropathic pain; moderate functional limitation, e.g. cannot walk long distance; frequent nocturnal wakening* |
| 5 | **marked** peripheral neuropathy pain  *e.g. constant neuropathic pain; significant limitation of walking (short distances only); may require assistive device* |
| 6 | **severe** peripheral neuropathy pain  *e.g. neuropathic pain severe enough to require long-acting narcotic; requires assistive device - i.e., cane, walker.* |
| 7 | among the most **extreme** cases of peripheral neuropathy pain  *e.g. neuropathic pain severe enough to restrict subject to wheelchair* |

### 3.5.5. Rescue medication assessment

All subjects will receive acetaminophen 500 mg caplets as rescue medication, which may be taken up to two caplets 3 times per day (every 8 hours) up to the maximum of 100 caplets dispensed per visit (extra caplets are provided in the event that the interval between visits is extended). Subjects **should be instructed to take rescue medication only when absolutely necessary**. After Visit 2, rescue medication will be recorded in the electronic diary at the time of use. Further information with regard to rescue medication is given under Section 3.3.1, and Appendix 2.

## Safety assessments

Safety assessments will consist of monitoring and recording all adverse events and serious adverse events, the regular monitoring of safety laboratories, regular measurement of vital signs, and the performance of physical and neurological examinations. In addition, prosaptide serum antibodies and HIV-1 RNA will be measured, and nerve conduction studies, 12-lead EKGs, and the Karnofsky performance score completed. All adverse events must be recorded beginning at the time of informed consent, whether or not the subject has actually received study drug. Additional information with regard to safety assessments and recorded AEs is found under Sections 3.7.1-3.7.3.

### Laboratory evaluations

All laboratory safety tests will be performed by one central laboratory facility. Contact information for the laboratory, details about collection of samples, shipment of samples, reporting of results, and alerting of extreme values are given in the manual provided by the central laboratory facility (ICON Laboratories) supplied to each site. Appendix 6, Laboratory safety tests, lists the laboratory safety tests required during the study; the Study Flow Charts (see Table 3-1: Study flow chart, and Table 3-2: Flow chart for unscheduled and discontinuation visits) lists the visits at which these tests should be obtained.

ANY CLINICALLY SIGNIFICANT CHANGE IN LABORATORY TESTS FOUND DURING THE STUDY WILL BE EVALUATED AND A DECISION AS TO WHETHER THE SUBJECT SHOULD CONTINUE IN THE STUDY WILL BE MADE BY THE INVESTIGATOR IN CONJUNCTION WITH THE PROTOCOL CHAIR AND SAVIENT (the sponsor).

Notable abnormal values as defined in Section 7.1 (Criteria for clinically notable laboratory abnormalities) will be reported to the investigators by the central laboratory for immediate safety assessment. The purpose of these reports is to ensure investigators are made aware of any potential safety concerns regarding their patients in a timely manner. In addition, all notable abnormalities will be flagged in the study analysis according to Section 6.1.6 (Safety evaluation). Notable abnormalities which are treatment-emergent will be reported separately.

If a subject discontinues from the study for any reason after receiving study drug, laboratory tests will be performed at the **discontinuation visit**. Any new abnormal laboratory values found at the post-study evaluation must be repeated until an appropriate explanation has been made or until the values have returned to normal.

### Vital signs

Vital sign measurements will be performed at each study visit. These measurements will consist of respiratory rate, heart rate, blood pressure, temperature, and weight. Height (in bare feet or wearing only thin socks) will be measured at Visit 1.

### 3.6.3. Physical examination

A physical examination will be performed at **Visits 1, 4, 7,** and **8** (or upon discontinuation from the study). Ideally, the same clinician should perform all of the examinations for a given subject in the study. A complete physical examination will include general appearance, eyes, ears, nose, mouth/throat, neck chest/lungs, heart, abdomen, lymph nodes, extremities, vasculature and skin. More extensive examinations may be done at any time at the discretion of the investigator.

Information about the physical examination must be present in the source documentation at the study site. Significant findings that are present at Visit 1 must be included on the Medical History page of the CRF. Significant findings made after Visit 1 which meet the definition of an adverse event must be recorded on the Adverse Event page of the CRF.

### 3.6.4. Neurological examination

Neurological examinations will be performed at **Visits 1, 4, 7**, and **8** (or upon discontinuation from the study). All neurological examinations must be performed by a qualified neurologist. Ideally, the **same neurologist** should perform all of the examinations for a given subject. A complete neurological examination will include grading of sensory symptoms, grading of motor symptoms, pin sensibility, vibration sensibility using a 128-Hz tuning fork, motor function and tendon reflex testing, and cranial nerve testing. In addition, the neurologist completing the neurological examination must also complete the neuropathy assessment.

Information regarding the neurological examination must be present in the source documentation at the study site. Findings present at Visit 1 must be included on the Neurologic Examination CRF. Significant findings after Visit 1 that meet the definition of an adverse event must be recorded on the Adverse Event page of the CRF. Variations in the neurological examination and neuropathy assessment that are consistent with the underlying disease are not to be routinely captured as adverse events since they will be noted on the Neurologic Examination CRF.

### Nerve conduction studies

Nerve conduction evaluations will include unilateral antidromic sural nerve conduction and unilateral peroneal motor nerve conduction. Amplitude, latency and velocity will be calculated for both sural and peroneal nerves. These evaluations will be performed at **Visit 2** for stratification and at **Visit 7** as a safety measurement.

All nerve conduction studies must be performed by an experienced electromyographer or a technician who is under the direct supervision of an experienced electromygrapher. All studies will be assessed by the “Core Laboratory”, Washington University in St. Louis, MO.

#### Certification

Prior to performing study-related nerve conduction evaluations, each study site must be certified by the “core laboratory”. To receive certification, the site must receive “core laboratory” approval of 2 nerve conduction evaluations for HIV-uninfected control subjects. The original nerve conduction tracing will be retained by the site as source document and a copy will be faxed to the core laboratory for quality assurance. After sign off by the core laboratory, data from study subjects may be entered onto the CRF by the sites.

Details regarding procedures for nerve conduction testing are available in Appendix 7.

### Karnofsky score

The Karnofsky performance score^24^ will be performed at **Visit 1** as an entrance criterion and also at the end of the 6 week double blind treatment period (**Visit 7**) in order to track functional performance of study subjects. Subjects must have a Karnofsky score of ≥60 in order to enter the study. Table 3-5 displays scoring categories.

###### Table 3-5: Karnofsky performance score

| **Description** | **Score** |
| --- | --- |
| **normal** with no complaints or evidence of disease | 100 |
| able to **perform normal activity** with minor signs and symptoms of disease | 90 |
| able to **perform normal activity with effort** but with some signs and symptoms of disease | 80 |
| **cares** for self but is **unable** to perform normal activity or to do active work | 70 |
| requires **occasional assistance** but is able to care for most of own needs | 60 |
| requires **considerable assistance** and **frequent** medical care | 50 |
| requires **special care** and assistance and is disabled | 40 |
| **hospitalization** indicated although **death not imminent** but is severely disabled | 30 |
| **hospitalization necessary** and active **supportive treatment required** and is very sick | 20 |
| fatal processes progressing rapidly and is **moribund** | 10 |
| is **dead** | 0 |

## Adverse event definitions, evaluation criteria and reporting

### Adverse event definitions

#### Adverse events

An adverse event is any untoward event whether or not considered related to the use of study drug. Study drug includes the drug under evaluation, and the look-alike placebo given during active treatment of the study. **Any worsening** (i.e. **any clinically significant adverse change in frequency or intensity**) **of a pre-existing condition which is temporally associated with the use of the sponsor’s product, is also an adverse event**. Unless a worsening occurs in these baseline conditions, they should NOT be considered adverse events.

Abnormal **laboratory values** or test results constitute adverse events only if they are associated with or induce clinical signs or symptoms or require therapy, and are recorded on the Adverse Events page of the CRF under the signs, symptoms or diagnosis associated with them.

#### Serious adverse events

A serious adverse event is any adverse event that results in any of the following outcomes:

- death
- is life-threatening (places the subject, in the view of the investigator, at immediate risk of death from the event as it occurred)
- inpatient hospitalization or prolongation of existing hospitalization (hospitalization is defined as an inpatient admission, regardless of length of stay, even if hospitalized as a precautionary measure for continued observation)
- a persistent or significant disability/incapacity (substantial disruption of the ability to conduct normal life functions)
- a congenital anomaly/birth defect
- a medically significant event that may jeopardize the subject and may require medical or surgical intervention to prevent one of the outcomes listed in this definition

Events NOT considered to be serious adverse events are:

- hospitalization for treatment, which was elective or pre-planned, for a pre-existing condition that did not worsen
- treatment on an emergency, outpatient basis for an event NOT fulfilling any of the definitions of serious given above and NOT resulting in hospital admission
- an adverse event that, had it occurred in a more severe form, might have caused death

#### Defining treatment-emergent AEs including SAEs

A treatment-emergent adverse event is ANY event that emerges during treatment, having been absent during pretreatment, or worsens relative to the pretreatment state. For the purposes of this study, treatment-emergent adverse events will be defined as those events occurring after the first dose of study drug at **Visit 4**.

### 3.7.2. Adverse event evaluation criteria

#### Relationship to study drug

The determination of the likelihood that the test drug caused the adverse event will be provided by the investigator. The investigator’s signature and date on the source document or case report form that supports the causality noted on the AE page of the CRF, ensures that a medically qualified assessment of causality was done. The signed document must be retained for the required regulatory time frame. The criteria below are intended as reference guidelines to assist the investigator in assessing the likelihood of a relationship between the test drug and the adverse event.

The following components will be used to assess this relationship; the greater the correlation with the components and their respective elements, the more likely the study drug caused the adverse event.

- **Exposure**: Is there evidence that the subject was actually exposed to the study drug such as: reliable history, acceptable compliance assessment, expected pharmacologic effect, or measurement of drug/metabolite in bodily specimen?
- **Time course**: Did the AE follow in a reasonable temporal sequence from administration of the study drug? Is the time of onset of the AE compatible with a drug-induced effect?
- **Likely Cause**: Is the AE not reasonably explained by another etiology such as underlying disease, other drug(s), or other host or environmental factors?
- **De-challenge**: Was the dose of study drug discontinued or reduced? If yes, did the AE resolve or improve? If yes, this is a positive de-challenge. If no, this is a negative de-challenge.

Note: This criterion is not applicable if: 1) the AE resulted in death or permanent disability, 2) the AE resolved or improved despite continuation of the study drug, or 3) the study is a single-dose drug study.

- **Re-challenge**: Was the subject re-exposed to the test drug in this study? If yes, did the AE recur or worsen? If yes, this is a positive re-challenge. If no, this is a negative re-challenge (note: this criterion is not applicable if: 1) the initial AE resulted in death or permanent disability, or 2) the study is a single-dose drug study).

IF A RE-CHALLENGE IS PLANNED FOR AN ADVERSE EVENT WHICH WAS SERIOUS AND WHICH MAY HAVE BEEN CAUSED BY THE STUDY DRUG, OR IF RE-EXPOSURE TO THE STUDY DRUG POSES ADDITIONAL POTENTIAL SIGNIFICANT RISK TO THE SUBJECT, THEN THE RE-CHALLENGE MUST BE APPROVED IN ADVANCE BY SAVIENT (THE SPONSOR) AND BY THE INDEPENDENT ETHICS COMMITTEE/INSTITUTIONAL REVIEW BOARD.

- **Consistency with the study drug profile**: Is the clinical and pathological presentation of the AE consistent with previous knowledge regarding the study drug or drug class pharmacology or toxicology?

The assessment of relationship will be reported by the investigator according to his/her best clinical judgment, including consideration of the above elements. The following scale of criteria may be used as a guidance (not all criteria must be present in order to be indicative of a drug relationship).

**Probably related to study drug:**

- there is evidence of exposure to the study drug
- the temporal sequence of the AE onset relative to administration of the study drug is reasonable
- the AE is more likely explained by the study drug than by another cause
- re-challenge (if performed) is positive
- de-challenge (if performed) is positive

**Possibly related to study drug:**

- there is evidence of exposure to study drug
- the temporal sequence of the AE onset relative to administration of the study drug is reasonable
- the AE could have been due to another equally likely cause
- de-challenge (if performed) is positive

**Unlikely related to study drug:**

- there is evidence of exposure to the study drug
- there is another more likely cause of the AE
- de-challenge (if performed) is negative or ambiguous
- re-challenge (if performed) is negative or ambiguous
- there is no temporal relationship to study drug

#### Adverse event severity (intensity)

The following definitions are to be used in grading the severity of adverse events:

**Mild**: A sign, symptom or event that is noticeable but easily tolerated. It does not significantly influence performance or prevent the subject or patient from carrying on with usual life activities. Treatment intervention may be required.

**Moderate**: A sign, symptom or event that causes enough discomfort to interfere with usual life activities. Treatment intervention may be required.

**Severe**: A sign, symptom or event that causes severe discomfort to the subject and significantly affects clinical status or the ability to perform usual life activities. Treatment intervention is warranted.

SERIOUS AND SEVERE ARE NOT SYNONYMOUS. A SERIOUS ADVERSE EVENT MUST FULFILL THE REQUIREMENTS LISTED IN THE DEFINITION.

### 3.7.3. Adverse event reporting

Information regarding all adverse events, discovered by investigator, or detected through physical examination, laboratory test or other means, will be collected and recorded on the Adverse Event page of the CRF or Serious Adverse Event page of CRF and followed as appropriate. Adverse events must be reported for the entire duration of the study, not just during administration of study drug. Treatment-emergent adverse events will be listed separately in the final clinical study report.

As far as possible, each adverse event will also be described by:

- its **duration** (start and end dates),
- the **severity** grade (mild, moderate, severe),
- its **relationship** to the study drug (probably related, possibly related, and unlikely),
- and the **action(s)** taken and, as relevant, the outcome.

ANY SERIOUS ADVERSE EVENT, OR DEATH DUE TO ANY CAUSE, WHICH OCCURS TO ANY SUBJECT IN THIS STUDY OR WITHIN 30 DAYS FOLLOWING CESSATION OF TREATMENT, WHETHER OR NOT RELATED TO THE INVESTIGATIONAL PRODUCT, MUST BE REPORTED WITHIN 24 HOURS TO ONE OF THE INDIVIDUAL(S) LISTED ON THE CONTACT INFORMATION PAGE (THE SPONSOR’S REPRESENTATIVE). ALL SUBJECTS WITH SERIOUS ADVERSE EXPERIENCES MUST BE FOLLOWED UP FOR OUTCOME.

**Instructions for SAE recording and reporting**

The study site will make copies of the blank initial and follow-up SAE report forms as needed. The site will retain originals of all information faxed to PPD Development in the Investigator Site File. Any Serious Adverse Event (SAE) must be **reported immediately** by telephone to the Medical Affairs/Pharmacovigilance Department at PPD Development at **800-201-8725**. A written description of any SAE must be sent to PPD Development by **facsimile** (**888-488-9697**) **within 24 hours** of its occurrence. The site personnel will refer to the protocol for further clarification.

**Note:** **If there is any question as to whether an event should be reported, call PPD to discuss the event in question at 800-201-8725.**

## 3.8. Drug levels and pharmacokinetic assessments

No drug levels other than study drug will be assessed. Blood samples for pharmacokinetic analysis of prosaptide will be drawn from subjects at **designated** study sites only. It is anticipated that approximately 100 study subjects will participate in the pharmacokinetic testing.

Pharmacokinetic analysis will be performed according to a predetermined randomization schedule at Visits 4, 6 and 7. Subjects will be randomized to one of three sampling sequences (ABC, BCA, CAB) and will have samples drawn at the following schedule as indicated under Table 3-6. Subjects participating in the pharmacokinetic analysis will take their dose of study medication for the day at the clinic visit. The **dose at Visit 4** should be a vial from the **Week 1** carton. The **dose at Visit 6** should be a vial from **Week 4** carton, and the **dose at Visit 7** should be a vial from the **Week 6** carton.

###### Table 3-6: Blood sample collection schedule for pharmacokinetic analysis

| **Schedule** | **Time** |
| --- | --- |
| A | predose, **1** hour and **3** hours postdose |
| B | predose, **1.5** hours and **4** hours postdose |
| C | predose, **0.5** hours and **2** hours postdose |
| Note: All subjects will be required to have blood samples taken within ±15 minutes of the specified time following drug dosing. Further details are given under Appendix 2, Section 1.2-AP2. | |

The study coordinator at each site will document the exact time of dose administration as well as the exact timing of the blood sample on the CRF. All subjects will be required to have blood samples taken within ±15 minutes of the specified time following dosing administration for pharmacokinetic plasma analysis.

Statistical methods for the pharmacokinetic analysis are given under Section 6.1.7.

# 4. Protocol amendments and other changes in study conduct

## 4.1. Protocol amendments

Any changes to the protocol will be made in the form of an amendment. Based on the results of the interim futility analysis, it is possible that one or more treatment arms may be dropped from the study. If this occurs, a protocol amendment will be issued to all sites delineating this change.

## 4.2. Other changes in study conduct

Changes in study conduct are not permitted. Any unforeseen changes in study conduct will be recorded in the clinical study report.

# 5. Data management

## Data collection

Investigators must enter the subject information required by the protocol onto the study sponsor’s case report forms (CRFs) that are printed on 3-part, non-carbon required (NCR) paper. The original and one copy of the CRFs will be forwarded to the CRF desk of the sponsor’s representative (PPD). One copy will be retained at the investigational site and is subject to the required document retention policy (see Appendix 1 Section 4. Document retention). Once the CRFs are received by PPD, their receipt is recorded, the original copy is placed in the study files and the NCR copy is forwarded to the responsible data management staff for processing.

During their regular visits, a study monitor will review the CRFs for completeness and accuracy, and instruct site personnel to make any required corrections or additions to CRFs that are at the site. Study representative (the study monitor) initiated data queries will be utilized for any corrections required to CRFs that have already been forwarded to PPD at the time of the study monitor’s visit.

Additional information is given under Appendix 2, Section 2.3.3-AP2 of this protocol.

## Database management and quality control

Data items from the CRFs are entered into the study database using appropriate methodology to ensure that the CRFs are accurately represented.

Information entered into the database is subjected to systematic logic and consistency checking routines. Obvious errors will be corrected by the appropriate data management personnel in accordance with the data handling and entry guidelines for the study. Findings from the electronic process defined above are reviewed by qualified data management or clinical personnel, after which electronically generated query forms are returned to the investigational site for resolution. Each site is expected to respond promptly to data queries. **The investigator** **must sign and date all responses to queries from CRF pages requiring an investigator signature**. Other query responses may be signed by either the investigator or a designee. After signature, the original query form should be sent to the sponsor’s representative (PPD) for database update and a copy filed with the subject’s CRF. Quality control edit checks of key safety and efficacy data in the database will be made regularly throughout the duration of the study.

Concomitant medications entered into the database will be coded using the WHO Drug Dictionary which classifies according to therapeutic/pharmacological class. Coexistent diseases and adverse events will be coded using MedDRA.

Laboratory samples including blood samples for prosaptide antibody determinations will be processed through the central laboratory facility (ICON Laboratories), and the results will be sent electronically to PPD with copies to the site. Prosaptide antibody and plasma drug levels will remain blinded until the conclusion of the study.

# 6. Statistical methods

## 6.1. Statistical methods

This is a double-blind, randomized, placebo controlled study with five dose groups (0, 2, 4, 8, 16 mg/per day). The primary efficacy endpoint is the change of the Gracely pain average score during Week 6 from the Week 0 baseline level (Week 0 minus Week 6 score). The primary objective is to select at least one dose group which is significantly better than the placebo with respect to this endpoint. Furthermore, the dose response relationship among these four groups will be examined.

Due to the slow enrollment, the protocol is amended to restrict further enrollment in the lowest dose arm (2 mg). It is expected that a sufficient number of subjects will have been treated at 2 mg before the amendment is fully implemented. An inferential statistical comparison between the 2 mg and placebo groups will still be carried out if there is a sufficient statistical power to detect a clinically meaningful treatment difference. Regardless, all dose groups will be used to characterize safety and dose response.

The study will be stratified with respect to SNAP (+ or -). For each stratum, a random treatment allocation rule will be utilized so that the balance among these five dose groups (four dose groups after the amendment) within each center will be maintained during the study.

It is planned that the data from all centers that participate in this protocol will be combined, so that an adequate number of subjects will be available for analysis. The results from each center will also be presented individually. In addition, it is planned that the data from both SNAP positive and SNAP negative subjects will be combined in the final analysis if there is no interaction between treatment effects and SNAP status.

The significance for all statistical tests will be reported at the p=0.05 level, and all tests will be 2-sided. Each dose group will be compared to the placebo group and a 95% confidence interval for each comparison will be provided. Because this is a phase 2 study, no adjustments for multiplicity will be made.

Since subjects receiving active treatment during the 42-day double-blind period will receive placebo injections for 2 weeks at visit 7 and those receiving placebo will be crossed over to receive prosaptide 4 mg, within group comparisons between visits 7 and 8 can be performed to examine the treatment effects due to the cross over. Change from visit 7 by dose group for the following parameters will be summarized: Gracely pain scale, McGill pain questionnaire, global physician impression, and safety laboratories.

### 6.1.1. Populations

Intent-to-treat subjects are defined as all randomized subjects who receive at least one dose of study medication and have some follow-up data. Per protocol subjects are a subset of the intent-to-treat subjects who complete the study without any major deviations from the protocol procedures. The primary efficacy analysis will be carried out for the intent-to-treat population, and the analysis will also be performed for the per protocol population. The safety analysis will be performed on the intent-to-treat population only.

### 6.1.2. Background and demographic characteristics

Demographic variables such as race, gender, and other baseline categorical parameters will be analyzed for treatment group comparability using the Chi-square test and Fisher’s exact test. Continuous type of parameters will be analyzed using the t-test and analysis of variance (ANOVA).

### 6.1.3. Study medication

Summary statistics for the total exposure to study drug during the 6-week double blind treatment period (number of doses) will be computed for each treatment group.

### 6.1.4. Concomitant therapy

Concomitant medications will be summarized by therapeutic/pharmacological class and treatment group. In addition, they will be listed by treatment group, name of drug, dates administered and indication.

Use of rescue pain medication will be evaluated as an efficacy parameter.

### 6.1.5. Efficacy evaluation

The primary analysis will be an intent-to-treat analysis. The standard 95% confidence intervals for treatment differences between each dose and placebo groups will be constructed for the primary efficacy endpoint. Since the study is stratified with respect to the SNAP status, separate confidence intervals will be constructed for each stratum. If there is no interaction between treatment efficacy and SNAP, the two intervals will be combined across the strata to have a global confidence interval for evaluating each dose effect. If the safety profiles for the four dose groups are similar, the recommended dose will be the one with the best efficacy (largest lower bound of the confidence interval).

Similar analyses will be repeated for the secondary efficacy endpoints. For the secondary endpoint analyses, the weekly pain scores will be calculated for each subject. In order to take advantage of such repeated measurements in the analysis, a 95% confidence interval of the difference between a dose group and the placebo will be constructed at each week. These intervals will be combined using the technique by Wei and Johnson^25^ to obtain a single more informative confidence interval for the treatment difference over the entire study period.

Subjects with greater than 0.35-unit pain improvement on the 13-point Gracely scale at Week 6 will be classified as treatment successes. Treatment group comparisons will be carried out using the Chi-square test and Fisher’s exact test. The standard confidence interval of the difference of the weekly success rates between a dose group and placebo will be constructed. For this dichotomized response, a single interval estimate across the entire study period will be computed using the Wei and Johnson technique.

In addition, an analysis of covariance (ANCOVA) which adjusts for the differences in baseline Gracely pain scale will be conducted for both primary and secondary efficacy endpoints. Primary and secondary efficacy analyses will also be repeated adjusting for other factors that may affect the outcome. Candidate predictive factors are SNAP amplitude at baseline, type of sensory neuropathy (DSP versus ATN), CD4 counts, plasma viral load, age, body mass index (BMI), duration of disease, and background antiretroviral therapy.

Additional efficacy analyses will include per protocol analyses. The previously described statistical approach will be repeated for this population.

For those subjects in the prosaptide groups who were treatment successes, the mean Gracely pain scale during the crossover placebo period will be calculated. The time at which the mean Gracely pain scale returns to the baseline level will be summarized by treatment group.

### 6.1.6. Safety evaluation

The assessment of safety will be based mainly on the frequency of adverse events and on the number of laboratory values that fall outside of pre-determined ranges. The safety measures will also include vital signs, physical examination, neurological examination, HIV-1 RNA, EKG, Karnofsky performance score, nerve conduction studies and prosaptide serum antibodies.

Adverse events will be summarized by presenting, for each treatment group, the number and percentage of subjects having any adverse event, having an adverse event in each body system and having each individual adverse event. Any other information collected (i.e. severity or relatedness to study medication) will be listed as appropriate.

Laboratory data will be summarized by presenting shift tables using extended normal ranges (baseline to most extreme post-baseline value), by presenting summary statistics of raw data and change from baseline values (means, medians, standard deviations, ranges) and by the flagging of notable values (as defined in section 7.1) in data listings. Two (or more)-way frequency tables of changes in laboratory test results in terms of normal ranges at the follow-up visits versus baseline by treatment group, as well as changes in absolute values from baseline will be examined for treatment differences. Change from baseline of plasma HIV-1 RNA levels by treatment group will be specifically evaluated.

Data from other tests (i.e. electrocardiogram or vital signs) will be listed, notable values will be flagged, and any other information collected will be listed as appropriate. Any statistical tests performed, such as the Chi-square test and the t-test, to explore the data will be used only to highlight any interesting comparisons that may warrant further consideration.

### 6.1.7. Statistical methods for pharmacokinetic analysis

The sparse plasma concentration data collected in this study will be utilized in a subsequent population pharmacokinetic analysis using a non-linear mixed effects modeling approach. The software package, NONMEM (NONlinear Mixed Effects Models) version V, Level 1.1 will be used to estimate population means and variances of specific pharmacokinetic parameters, such as clearance (CL) and volume of distribution (V). The relationship between the pharmacokinetics of prosaptide (CL, V) and covariates will be explored. The following covariates will be examined for a possible relationship to prosaptide drug exposure and include but are not limited to: age, body weight, height, body mass index, race, serum creatinine, creatinine clearance (as calculated by Cockcroft-Gault), aspartate transaminase, alanine transferase, alkaline phosphatase, serum albumin, total protein, and concomitant medications.

### 6.1.8. Interim analysis

A single interim analysis of the primary efficacy endpoint will be conducted after the first 200 subjects have completed the 6-week double-blind treatment period. Treatment arms determined to have minimal possibility of efficacy in a futility analysis will be dropped from the study. In addition, an estimate of variance of the primary endpoint will be performed and an evaluation of effect of SNAP will be assessed. Because the study will not be terminated due to any premature treatment efficacy, there should NOT be a penalty on the global Type I error rate for this interim look.

For the interim analysis, the standard deviation of the changes of the pain score will be estimated to see if the assumed standard deviation of 0.35 is valid. Next, a “predicted” 95% confidence interval for the treatment difference between each dose and placebo groups will be computed for the primary efficacy endpoint. If the confidence interval for a dose group does not include 0.17 (projected placebo effect, see Section 6.3 for justification), this dose group will be dropped from the study. However, this treatment effect of 0.17 will be re-estimated from the interim analysis data, and the decision rule can be modified based on the new estimate. If subjects with a negative SNAP do not benefit from the treatment, future subjects with a negative SNAP will not be enrolled in the study. Finally, the sample size based on the new estimate of the standard deviation will be determined so that the length of the 0.95 confidence intervals of the difference of changes between a dose group and the placebo will be within 0.24 (twice of 0.12, see Section 6.3 Sample size and power considerations) at the final analysis.

The interim analysis will be performed by CBAR at the Harvard School of Public Health. The results will be shared only with members of the independent Data Safety Monitoring Board (DSMB), who will make recommendations to the Protocol Chair and the study team regarding protocol modifications. In order to avoid bias, the DSMB will not disclose any findings except for issuing a decision statement. All staff, including the Savient clinical team and investigators, involved in the conduct of the study will remain blinded to the results of such analyses.

## 6.2. Safety monitoring and periodic safety reporting

A periodic safety report will be provided to the Data Safety Monitoring Board and external personnel once every 6 months without breaking the blind, as detailed in the Monitoring Plan developed by CBAR. The report will include summary tables of the safety parameters listed in Section 6.1.6. If there are any safety concerns, the committee will request the trial statistician to perform additional analyses by treatment. Further, summary information on Serious Adverse Events will be provided to the DSMB, DAIDS and the study team as they occur.

## Sample size and power considerations

Based on two studies of neuropathic pain in subjects with HIV-SN^20, 21^, the average placebo response in the Gracely pain scale is about 0.17, and the standard deviation of the change in Gracely pain scale is approximately 0.35. Therefore, any treatment differences of less than 0.17 can be considered to be not clinically meaningful. This study is designed to detect a minimum treatment difference of 0.17 between any dose group and placebo. In order to have at least 80% power to detect this difference and a significance level of 0.05 (2-sided), 70 evaluable subjects at the end of the trial in the 4 mg, 8 mg, 16 mg and placebo groups are required. The power to detect this difference between the 2 mg and placebo groups will also be calculated before the final efficacy analysis. With 70 subjects per group, the 95% confidence interval for the changes between any dose group and the placebo covers (d-0.12, d+0.12), where d is the observed difference between the two groups. Allowing for early dropouts and assuming the enrollment of 45 subjects in the 2 mg group, 355 subjects will need to be enrolled for the final analysis.

# 7. Notable laboratory value criteria, special methods and scales

## 7.1. Criteria for clinically notable laboratory abnormalities

Safety laboratories for this study will include serum chemistry, hematology and liver function tests. Urinalysis will also be performed along with safety laboratories (see Appendix 6). Due to the advanced state of immunodeficiency and multiple concomitant medications anticipated for subjects in this study, a greater variability in laboratory tests is anticipated than would be seen in a normal adult population. In addition, it is anticipated that most subjects will have multiple other concomitant conditions that may adversely influence laboratory tests. Therefore, **determination of causality for laboratory abnormalities will be particularly difficult and will require active, ongoing clinical correlation on the part of the investigator**. The central laboratory will report the predetermined clinically notable laboratory results as specified below to the investigative site immediately. The investigator is responsible for evaluating the laboratory abnormality as a potential adverse event, and for determining required follow-up. Source records should clearly document all follow-up performed.

For the purposes of this study, **clinically notable serum chemistry** abnormalities will include blood urea nitrogen (BUN) >3 times the upper limit of normal (ULN), creatinine >3 times ULN, SGOT or SGPT >5 times ULN. Since values of total bilirubin are influenced by concomitant medications, a total bilirubin of >5 times ULN will be considered clinically notable. **Clinically notable hematology** values will include a hemoglobin of <6.9 g/dL, a total white blood cell count of <2 x 10^9^/L or ≥ 27.75 x 10^9^/L, and a platelet count of <50 x 10^9^/L or ≥1000 x 10^9^/L.

## Special methods and scales

Anti-prosaptide antibody will be measured at Visit 7 via an ELISA assay. Details regarding collection and shipment of samples are given in the investigator folder supplied to each site. Results from the prosaptide antibody tests and prosaptide drug levels will remain blinded to investigators as well as all NARC and Savient study personnel until after the database lock and unblinding of the database at the conclusion of the study.

Further information is provided under Appendix 2, Section 2.2-AP2.

# 8. Reference list

1. Campana WM, Hirairwa M, Addison KC, O'Brien JS. Induction of MAPK phosphorylation by prosaposin and prosaptide in PC12 cells. Biochemical and Biophysical Research Communications 1996; 229:706-712.

2. Hirairwa M, Martin BM, Kishimoto Y, Conner GE, Tsuji S, O'Brien JS. Lysosomal proteolysis of prosaposin, the precursor of saposins (sphingolipid activator proteins): Its mechanism and inhibition by ganglioside. Archives of Biochemistry and Biophysics 1997; 341:17-24.

3. Hiraiwa M, Taylor E, Campana W, Darin S, O'Brien J. Cell death prevention, mitogen-activated protein kinase stimulation, and increased sulfatide concentrations in Schwann cells and oligodendrocytes by prosaposin and prosaptides. USA Proc. Nat. Acad. Sci 1997; 94:4778-4781.

4. O'Brien J, Carson G, Seo H, Hiraiwa M, Kishimoto Y. Identification of prosaposin as a neurotrophic factor. USA Proc. Nat. Acad. Sci 1994; 91:9593-9596.

5. O'Brien J, Carson G, Seo H, et al. Identification of the neurotrophic factor sequence of prosaposin. FASEB J. 1995; 9:681-685.

6. Calcutt N, Freshwater J, O'Brien J. Protection of sensory function and antihyperalgesic properties of prosaposin-derived peptide in diabetic rats. Anesthesiology 2000; 93:1271-1278.

7. Otero D, Conrad B, O'Brien J. Reversal of thermal hyperalgesia in a rat partial sciatic nerve ligation model of Prosaptide TX14(A). Neuroscience Letters 1999; 270:29-32.

8. Wagner R, Myers R, O'Brien J. Prosaptide prevents hyperalgesia and reduces peripheral TNFR1 expression following TNF-alfa nerve injection. Neuroreport 1998; 9:2827-2831.

9. Calcutt NA, Campana WM, Eskeland NL, et al. Prosaposin gene expression and the efficacy of a prosaposin-derived peptide in preventing structural and functional disorders of peripheral nerve in diabetic rats. Journal of Neuropathology and Experimental Neurology 1999; 58:628-636.

10. Mizisin A, Steinhardt R, O'Brien J, Calcutt N, TX14(A). A prosaposin-derived peptide, reverses established nerve disorders in streptozotocin-diabetic rats and prevents them in glalactose-fed rats. J. Neuropathol Exp. Neurol. 2001; 60:953-960.

11. Campana W, Eskeland N, Calcutt N, Misasi R, Myers R, O'Brien J. Prosaptide prevents paclitaxel neurotoxicity. Neuro Tox 1998; 19:237-244.

12. Sacktor N, Lyles R, Skolasky R, et al. HIV-associated neurologic disease incidence changes: Multicenter AIDS Cohort Study. Neurology 2001; 56:257-260.

13. Baceller H, Munoz A, Miller E, et al. Temporal trends in the incidence of HIV-1-related neurologic diseases: Multicenter AIDS Cohort Study, 1985-1992. Neurology 1984; 44:1892-1900.

14. Wulff E, Wang A, Simpson D. HIV-associated peripheral neuropathy: epidemiology, pathophysiology and treatment. Drugs 2000; 59:1251-1260.

15. Cornblath D, McArthur J. Predominantly sensory neuropathy in patients with AIDS and AIDS-related complex. Neurology 1988; 1938:794-796.

16. Childs E, Lyles R, Selnes O, et al. Plasma viral load and CD4 lymphocytes predict HIV-associated dementia and sensory neuropathy. Neurology 1999; 52:607-613.

17. Schifitto G, McDermott M, McArthur J, et al. Incidence of and risk factors for HIV-associated distal sensory polyneuropathy. Neurology 2002; 58:1764-1768.

18. Moore RD, Wong W-ME, Keruly JC, McArthur J. Incidence of neuropathy in HIV-infected patients on monotherapy versus those on combination therapy with didanosine, stavudine and hydroxyurea. AIDS 2000; 14:273-278.

19. Cherry CL, Gahan ME, McArthur J, Lwein SR, Hoy JF, Wesselingh SL. Exposure to dideoxynucleosides is reflected in lowered mitochondrial DNA in subcutaneous fat. Journal of Acquired Immunoe Deficiency Syndrome 2002; 30:271-277.

20. McArthur J, Yiannoutsos C, Simpson D, et al. A phase II trial of nerve growth factor for sensory neuropathy associated with HIV infection. Neurology 2000; 54:1080-1088.

21. Kieburtz K, Simpson D, Yiannoutsos C, et al. A randomized trial of amitriptyline and mexiletine for painful neuropathy in HIV infection. AIDS Clinical Trial Group 242 Protocol Team. Neurology 1998; 51:1682-1688.

22. Gracely RH, McGrath P, Dubner R. Ratio scales of sensory and affective verbal pain descriptors. Pain 1978; 5:5-18.

23. Melzack R. The short-form McGill Questionnaire. Pain 1987; 30:191-197.

24. O'Dell MW, Lubeck DP, O'Droscoll P, Matsuno S. Validity of the Karnofsky Performance Status in an HIV-infected sample. Journal of Acquired Immunoe Deficiency Syndrome 1995; 10:350-357.

25. Wei L, Johnson W. Combining dependent tests with incomplete repeated measurements. Biometrika 1985; 72:359-364.

| Appendices to the protocol |
| --- |

# Appendix 1: Ethics and good clinical practice

This study must be carried out in compliance with the protocol and in accordance with the sponsor’s (Savient) standard operating procedures. These are designed to ensure adherence to Good Clinical Practice, as described in:

1. ICH Harmonized Tripartite Guidelines for Good Clinical Practice 1996. Directive 91/507/EEC, The Rules Governing Medicinal Products in the European Community.
2. US 21 Code of Federal Regulations dealing with clinical studies (including parts 50 and 56 concerning informed consent and IRB regulations).
3. Declaration of Helsinki, concerning medical research in humans (Recommendations Guiding Physicians in Biomedical Research Involving Human Subjects, Helsinki 1964, amended Tokyo 1975, Venice 1983, Hong Kong 1989, Somerset West 1996).

The investigator agrees, when signing the protocol, to adhere to the instructions and procedures described in it and thereby to adhere to the principles of Good Clinical Practice that it conforms to. Copies of these regulations are available at:

[www.fda.gov/cder/guidance/959fnl.pdf](http://www.fda.gov/cder/guidance/959fnl.pdf)

[www.access.gpo.gov/nara/cfr/waisidx_00/21cfr50_00.html](http://www.access.gpo.gov/nara/crf/waisidx_00/21cfr50_00.html)

[www.access.gpo.gov/nara/cfr/waisidx_00/21cfr56_00.html](http://www.access.gpo.gov/nara/cfr/waisidx_00/21cfr56_00.html)

[www.fda.gov/oc/health/helsinki89.html](http://www.fda.gov/oc/health/helsinki89.html)

## 1.1-AP1. Institutional review board/Independent ethics committee

Before implementing this study and initiating any protocol-related activities, the protocol, the proposed informed consent form and other information to subjects must be reviewed by a properly constituted Institutional review board/Independent ethics committee (IRB/IEC). The original copy of the signed and dated statement that the protocol and informed consent have been approved by the IRB/IEC must be sent to sponsor’s representative (PPD) before study initiation. The investigator must maintain a copy of the approval letter that contains the study number, protocol title, and identification of all documents approved. The name and occupation of the chairman and the members of the IRB/IEC must also be supplied to PPD. If the investigator or any member of the study staff is also a member of the IRB/IEC, he or she must provide a written statement that he/she absented from voting on this study. Any amendments to the protocol, other than administrative ones, must also be approved by the IRB/IEC.

The investigator must submit annual status reports to the IRB/IEC and provide a copy to PPD. Within three months after completion or termination of the study a final report must be submitted to the IRB/IEC and PPD. The investigator must maintain an accurate and complete record of all submissions made to the IRB including a list of all reports and documents submitted.

The IRB will comply with all federal, state, and local laws. The investigator will obtain from the IRB/ERC and submit to PPD a signed statement indicating that it complies with Good Clinical Practices. A sample IRB compliance letter is provided in the investigator folder at each site.

The sponsor (Savient) and the sponsor’s representative (PPD) will promptly be advised of any regulatory inspection (relating to this study) of either the institution or the IRB. The investigator will promptly provide a copy of any inspection report.

## 1.2-AP1. Informed consent (IC)

The investigator must explain to each subject or subject’s legally authorized representative the nature of the study, its purpose, the procedures involved, the expected duration, the potential risks and benefits involved and any discomfort it may entail. Each subject or subject’s legally authorized representative must be informed that participation in the study is voluntary and that he/she may withdraw from the study at any time and that withdrawal of consent will not affect his/her subsequent medical treatment or relationship with the treating physician.

This informed consent should be given by means of a standard statement, written in non-technical language. The subject should read and consider the statement before signing and dating it, and should be given a copy of the signed document. No subject can enter the study before informed consent has been obtained.

The study specific informed consent form must be submitted by the investigator with the protocol for IRB/IEC approval. The sponsor supplies a proposed informed consent form, which complies with regulatory requirements and is considered appropriate for the study. Any changes to the proposed consent form suggested by the investigator must be agreed to by Savient, and a copy of the approved version must be provided to the PPD monitor after IRB/IEC approval.

A copy of the FDA regulations with regard to informed consent, the World Medical Association Declaration of Helsinki, and a sample consent form are available at:

[www.access.gpo.gov/nara/cfr/waisidx_00/21cfr50_00.html](http://www.access.gpo.gov/nara/crf/waisidx_00/21cfr50_00.html)

[www.fda.gov/oc/health/helsinki89.html](http://www.fda.gov/oc/health/helsinki89.html)

## 1.3-AP1. Compliance with law, audit, and debarment

By signing this protocol, the investigator agrees to conduct the study in an efficient and diligent manner and in conformance with this protocol; generally accepted standards of Good Clinical Practice; and all applicable federal, state, and local laws, rules and regulations relating to the conduct of the clinical study.

The investigator also agrees to allow monitoring, audits, Institutional review board/Independent ethics committee review and regulatory agency inspection of study-related documents and procedures and provide for direct access to all study-related source data and documents.

The investigator agrees not to seek reimbursement from subjects, their insurance providers, or from government programs for procedures included as part of the study reimbursed to the investigator by Savient.

The investigator shall prepare and maintain complete and accurate study documentation in compliance with Good Clinical Practice standards and applicable federal, state, and local laws, rules and regulations. For each subject participating in the study, the investigator shall provide all data and submit any other reports to PPD as required by this protocol or by any other agreement with Savient.

Study documentation (see Document retention section) will be promptly and fully disclosed to PPD and Savient by the investigator upon request and also shall be made available at the investigator’s site upon request for inspection, copying, review and audit at reasonable times by representatives of Savient or any regulatory agencies. The investigator agrees to promptly take any reasonable steps that are requested by Savient as a result of an audit to cure deficiencies in the study documentation and CRFs.

Persons debarred from conducting or working on clinical studies by any court or regulatory agency will NOT be allowed to conduct or work on Savient studies. The investigator will immediately disclose in writing to Savient if any person who is involved in conducting the study is debarred, or if any proceeding for debarment is pending or, to the best of the investigator’s knowledge, threatened.

## 1.4-AP1. Compliance with financial disclosure requirements

By signing this protocol, the investigator agrees to provide to Savient accurate financial information to allow submission of complete and accurate certification and disclosure statements as required by US Food and Drug Administration regulations (21 CFR Part 54). This requirement also extends to sub-investigators.

# Appendix 2: Procedures and instructions

## 2.1-AP2. Labeling, packaging, storage, and return of clinical supplies

### 2.1.1-AP2. Packaging of clinical supplies

#### Study drug (prosaptide or placebo)

Clinical supplies for 855 subjects (randomization numbers 90001-90885) will be provided. One or more blocks of five subjects will initially be sent to each individual center in validated, insulated containers with cold packs. Additional supplies will be packaged and held by Fisher Clinical Services under frozen conditions. Fisher will remove the 2 mg kit from the remaining unshipped blocks, so that each block will contain only four kits (4, 8 or 16 mg prosaptide, and placebo). Each subject will be assigned a kit containing eight cartons of study medication. There will be nine single use vials per carton.

Prosaptide is stable for at least 12 months when stored at -15° C. Prosaptide is stored frozen at -5^o^ C to -20^o^ C until shipment to the investigative centers. Shipment is performed by validated shipping containers that allow the study medication to remain below 8° C during transport.

INVESTIGATIVE SITES MUST STORE THE MEDICATION IN A SECURE FREEZER AT -15^o^C ±5^o^C. TWELVE ICE PACKS WILL BE INCLUDED IN THE SHIPMENT. THESE MUST BE TAKEN FROM THE SHIPPER AND STORED FROZEN AT THE SITE FOR DISTRIBUTING TO SUBJECTS WHEN DISPENSING THE FIRST 2 WEEKS OF STUDY SUPPLIES. THE STUDY SUBJECTS WILL BE INSTRUCTED THAT THE MEDICATION MUST BE STORED IN A REFRIGERATOR AT ALL TIMES AND WILL BE PROVIDED WITH A SMALL COOLER AND 2 COLD PACKS EACH FOR TRANSPORTING THE STUDY DRUG TO AND FROM THE CLINICAL CENTERS.

At 5°C, prosaptide is stable for up to 3 months. It is stable at room temperature for short periods of time. If left at room temperature for more than **24 hours**, **the sponsor’s representative (PPD) should be contacted for further instructions.**

Prosaptide or look-alike placebo is **dosed subcutaneously into the abdomen on a once daily basis**. One mL of prosaptide should be given at each administration regardless of dosage strength. Each vial is intended for a single subject dose, and any excess left in the vial after dosing should **not be used for further dosing.** Specific instructions for self-administration are provided in Appendix 3.

Look-like placebo will be supplied as sterile phosphate buffered saline with 3% mannitol, pH 6.8. Placebo is packed in 2 mL sterile glass vials with Teflon-faced stoppers and flip-off caps.

#### Rescue medication

Clinical supplies of rescue (acetaminophen 500 mg caplets) medication will be provided by Savient. Rescue medication supplies will be shipped in non-subject specific packaging at ambient temperature. The medication will be supplied in sealed child-proof containers with 100 caplets per bottle. It should be stored at room temperature in a locked storage area.

Two caplets of rescue medication may be taken on an as needed basis every 8 hours. Subjects should be instructed to record all rescue medication use in their electronic diaries.

Subjects should receive one bottle of rescue medication (100 acetaminophen caplets) at each visit. When dispensing rescue medication, the investigator or designee must write the subject number on each bottle for tracking purposes, and record the lot number on the Drug Accountability page of the CRF.

#### Other clinical supplies

In addition to the coolers and ice packs, subjects will also receive supply packages for study drug administration. Savient will provide non-subject specific “Sundry” packages containing supplies for a two-week period. One Sundry package should be given to the subject each time drug is dispensed. Each Sundry package will contain 20 syringes with needles, alcohol wipes, gauze pads, and two sharps disposal containers. The disposal containers and any unused syringes with needles should be returned to the study site with the used and unused vials.

### 2.1.2-AP2. Labeling

All kits will be labeled with a two-part subject specific label. Labels will be printed with the protocol number, randomization number, applicable storage conditions, Federally mandated caution statement and company identification. The kit labels will also include space for subject number, subject initials, investigator number and date dispensed. This information should be filled in at the time of subject randomization.

In addition, a blinded drug disclosure report containing the blinded treatment information will be provided with each kit. Treatment will remain blinded unless disclosure becomes necessary in the event of a medical emergency (See Section 3.3.3 Blinding). Each subject kit will contain 8 cartons of study drug.

A one-part label will be affixed to each carton containing 9 vials. The label will contain the following information:

- protocol number
- randomization number
- week to be used
- contents
- BTGC identification (name and address)

**Note:** The sponsor’s former name was Bio-Technology General Corp., which included an abbreviated designation, “BTGC.” Therefore, the labels on the study medication refer to the sponsor as “BTGC.”

- caution statement required by US federal law
- administration instructions
- storage instructions

Each carton label will also include space for subject number, subject initials, and investigator number. This information should be filled in on each all 8 cartons at the time of subject randomization. The space for date dispensed should be filled in at the time the individual carton is dispensed.

All vials will be labeled with a one-part subject specific label. Labels will be printed with the protocol number, randomization number, applicable storage conditions, Federally mandated caution statement and company identification.

### 2.1.3-AP2. Storage and return of clinical supplies

Investigational clinical supplies must be received by a designated person at the study site, handled and stored safely and properly, and kept in a secured location to which only the investigator and designated assistants have access. **All clinical supplies must be frozen at -15°C +/-5°C immediately upon receipt and for storage throughout the study**. Clinical supplies are to be dispensed only in accordance with the protocol. The investigator is responsible for keeping accurate records of the clinical supplies received from Savient, the amount dispensed to and returned from subjects, and the amount remaining at the conclusion of the study. The sponsor’s representative (PPD) should be contacted with any questions concerning drug handling or administration.

The investigator, or a qualified designee, must maintain an inventory of drug supplies received and dispensed. PPD will provide forms to document all inventory transactions. Upon completion or termination of the study, all clinical supplies, both used and unused, must be returned to PPD or authorized designee in the original containers. Study mediations may not be shipped from the investigator’s site until checked, packed and secured by the study monitor. Return shipments of drug supplies do not require dry ice or other refrigeration techniques.

Return all drug supplies to:

**Fisher Clinical Services, Inc**.

Iron Run Corporate Center

6575 Snowdrift Road

Allentown, PA 18106

phone: (610) 391-0800

## 2.2-AP2. Biological specimens

### 2.2.1-AP2. Laboratory safety

#### Supplies for laboratory safety tests

The central laboratory facility will provide each investigative site with specimen collection materials in pre-identified, visit-specific kits for specimens to be tested at the central laboratory facility. Each is labeled with Savient’s name, protocol number, investigator number, and visit. Requisition forms will also be included in each kit. All shipping materials, including cartons and pre-addressed air bills will be provided to simplify the process for the investigators. Kits and specimen collection supplies are bar-coded for optimum accuracy in processing and maintenance of a subject-specific audit trail, therefore, it is mandatory to use laboratory supplies provided for the study.

Additional information regarding laboratory testing and shipment of laboratory supplies is provided in the central laboratory manual provided to each site.

#### Labeling of laboratory safety specimens

The requisition form (provided by the laboratory facility in each kit) must be completed accurately by the study coordinator and enclosed with each shipment. The requisition forms contain the following information:

- subject’s number
- subject’s initials
- subject’s date of birth
- subject’s gender
- date of collection
- time of collection

#### Shipment of laboratory safety tests

All laboratory tests will be shipped by courier service or overnight express mail to the central laboratory. Additional information on shipment of specimens is provided in the central laboratory manual. It is the responsibility of the investigator to ensure that all staff personnel who will be handling, packaging, or shipping clinical specimens act in conformance with International Air Transport Association (IATA) regulations relating to the handling and shipping of hazardous goods.

### 2.2.2-AP2. Prosaptide antibody levels

All supplies for serum prosaptide antibody levels will be provided by the central laboratory in the visit specific kits. Samples will be shipped to the central laboratory in accordance with guidance provided in the central laboratory manual. Results of prosaptide antibody levels will remain blinded to the investigative sites and Savient until the conclusion of the study and unblinding of the subject database.

### 2.2.3-AP2. Pharmacokinetic analysis of plasma samples

All supplies for plasma samples for pharmacokinetic analysis will be provided by the central laboratory in the visit specific kits. Samples will be shipped to the central laboratory in accordance with guidance provided in the central laboratory manual. Results of prosaptide drug levels will remain blinded to the investigative sites and Savient until the conclusion of the study and unblinding of the database.

## 2.3-AP2. Administrative procedures

### 2.3.1-AP2. Changes to the protocol

Any change or addition to this protocol requires a written protocol amendment that must be approved by Savient and the Protocol Chair before implementation. Amendments significantly affecting the safety of subjects, the scope of the investigation or the scientific quality of the study, require additional approval by the IRB/IEC of all centers, and, in some countries, by the regulatory authority. A copy of the written approval of the IRB/IEC must be given to the PPD study monitor. Examples of amendments requiring such approval are:

- an increase in drug dosage or duration of exposure of subjects
- a significant change in the study design (i.e. addition or deletion of a control group)
- an increase in the number of invasive procedures to which subjects are exposed
- addition or deletion of a test procedure for safety monitoring

Amendments affecting only administrative aspects of the study do NOT require formal protocol amendments or IRB/IEC approval but the IRB/IEC of each center must be kept informed of such administrative changes. Examples of administrative changes that may not require formal protocol amendments and IRB/IEC approval, that may be or may not be treated as administrative amendments include:

- changes in the staff used to monitor trials (i.e. Savient staff versus a CRO)
- minor changes in the packaging or labeling of study drug.

### 2.3.2-AP2. Monitoring procedures

Investigational sites will be monitored on a regular basis according to PPD’s monitoring SOPs and study specific monitoring guidelines to assure satisfactory data recording, adherence to the protocol and Good Clinical Practice, and study medication accounting. The frequency of monitoring may vary depending on enrollment rate and the quantity of data collection, but must occur at least yearly. The investigator and staff are expected to assist with the study monitor’s review of all relevant study documentation, including source documents for each study subject. It is essential that the investigator and study coordinator set aside a sufficient amount of time for these visits to permit an adequate review of the study’s progress and of completed CRFs.

The investigator must give the monitor access to relevant hospital or clinical records, to confirm their consistency with the CRF entries. **No information in these records about the identity of the subjects will leave the study center**.

### 2.3.3-AP2. Data collection

Case Report Forms (CRFs) for the study will be provided by PPD. The investigator must complete the CRFs and transmit the data as instructed by PPD at study initiation and must store a copy of the CRF with other study documents, i.e. the protocol, the Investigator’ brochure and any protocol amendments, in a secure place. All entries to the CRFs must be made as described in the Case Report Form Completion Instructions or as instructed by PPD at study initiation.

Data on subjects collected on CRFs during the study will be documented in an anonymous fashion and the subject will only be identified by the subject number, and by his/her initials if also required. If, as an exception, it is necessary for safety or regulatory reasons to identify the subject, both PPD and the investigator are bound to keep this information confidential.

Discrepancies or questions concerning the data will be sent to the investigator using a systematic query resolution process. The discrepancy reports (“queries”) should be resolved by the investigator or study staff, signed and dated, and returned to PPD. A copy of the discrepancy report must be retained in the subject binder as a record of changes or acknowledgment of the receipt of queries on the data.

The investigator must maintain source documents for each subject in the study, consisting of all demographic and medical information, including laboratory data, electrocardiograms, etc, and keep a copy of the signed informed consent form. All information on CRFs must be traceable to these source documents kept in the subject's file. Data obtained without a separate written or electronic source document will be defined before study start and will be recorded directly on the CRFs, which will be documented as being the source data.

### 2.3.4-AP2. Document retention

Essential documents, as listed below, must be retained by the investigator for as long as needed to comply with national and international regulations (generally 2 years after discontinuing clinical development or after the last marketing approval). Savient will notify the investigator(s)/institution(s) when the study-related records are no longer required. If the investigator relocates, retires, or for any reason cannot keep the study records, the records may be transferred to an acceptable designee. Savient must be notified in writing of the name, address, and telephone number of the person designated to retain the study records. The investigator agrees to adhere to the document retention procedures by signing the protocol.

Essential documents include:

- IRB/IEC approvals for the study protocol and all amendments
- all source documents including laboratory and radiology records
- CRF copies
- data change forms or data queries
- subjects' informed consent forms (with study number and title of study)
- monitoring logs and appointment schedules
- FDA form 1572 (as required)
- investigator(s) curriculum vitae, medical license information, and financial disclosure documentation
- all sponsor/sponsor’s representative/investigator correspondence, including telephone logs
- any other pertinent study document

### 2.3.5-AP2. Disclosure and confidentiality

By signing the protocol, the investigator agrees to keep all information provided by Savient and PPD in strict confidence and to request similar confidentiality from his/her staff and the IRB/IEC. Study documents provided by Savient and PPD (protocols, Investigator' brochures, CRFs and other material) will be stored appropriately to ensure their confidentiality. The information provided by Savient and PPD to the investigator may not be disclosed to others without direct written authorization from Savient. Data generated by this study will be considered confidential by the investigator except to the extent that it is included in a publication as provided under Section 1.3.6-AP2 (Publication of results).

Individual subject data obtained during this study are confidential and will not be disclosed to third parties with the following exceptions:

- When data are needed by the subject’s personal physician or other medical personnel responsible for the subject’s welfare. Prior written consent from the subject or legal guardian must first be obtained.
- For data inspection and verification by Savient or designees (e.g., sponsor’s representative), IRB/ERC or regulatory agency representatives.

INDIVIDUAL SUBJECT IDENTITY CAN NOT BE DIVULGED IN ANY COMMUNICATION OR PUBLICATION.

### 2.3.6-AP2. Publication of results

Any formal presentation or publication of data from this trial will be considered as a joint publication by the investigator(s) and appropriate Savient personnel. Authorship will be determined by mutual agreement. For multicenter studies, it is mandatory that the first publication is based on data from all centers that has been analyzed as stipulated in the protocol. The final analysis of the study for publication will be performed by the Center of Biostatistics AIDS Research (CBAR) at the Harvard School of Public Health. Investigators participating in multicenter studies agree not to present data gathered from one center or a small group of centers before the full publication, unless formally agreed to by all other Investigators and Savient.

Savient must receive copies of any intended communication in advance of publication (at least 15 working days for an abstract or oral presentation and 45 working days for a journal submission). Savient will review the communications for accuracy (thus avoiding potential discrepancies with submissions to health authorities), verify that confidential information is not being inadvertently divulged and provide any relevant supplementary information. In accordance with uniform criteria for manuscripts, Savient will impose no impediment, direct or indirect, on publication of the study’s full results, including data perceived to be detrimental to the investigational product.

### 2.3.7-AP2. Discontinuation of study

Savient reserves the right to discontinue any study under the conditions specified in the clinical trial agreement. In the event that Savient prematurely terminates a particular study site, Savient’s representative will promptly notify that site’s IRB/ERC.

### 2.3.8-AP2. Changes in study personnel

If there is a change of any personnel listed on the FDA form FD 1572, a new form reflecting the change must be completed and forwarded to PPD, along with the new staff member’s signed curriculum vitae, medical license, and signed financial disclosure statement.

# Appendix 3: Study drug administration instructions

## 3.1-AP3. Study drug

Study drug is supplied as a clear, colorless, odorless solution packaged in sterile 2 mL glass vials. One mL of study drug should be given **subcutaneously** into the **abdomen** each day with the supplied syringe and needle.

To help avoid contamination and possible infection, follow these instructions for study drug administration exactly.

## 3.2-AP3. Drug supplies

At each study visit, you will be supplied with the following:

- weekly cartons of study drug each containing 9 single-dose vials
- alcohol wipes
- disposable syringes with needles
- 2 needle disposal containers
- gauze pads

**Syringe use**

Disposable syringes and needles should be used only once and then discarded in the needle disposal container. NEEDLES AND SYRINGES MUST NOT BE REUSED OR SHARED. Return used needles and syringes in the provided container to the study site for disposal.

**Preparing the dose**

1. Wash your hands.
2. Remove one vial of study drug from the refrigerator and bring to room temperature by allowing it to sit at room temperature for approximately 15 minutes. **Do not microwave or heat in any artificial fashion**.
3. Carefully shake or rotate the bottle several times to completely mix the study drug.
4. Inspect the study drug. It should be completely clear and colorless. Do not use if you notice anything unusual about the appearance, and notify the study site immediately.
5. Flip off the plastic protective cap from the bottle, but **do not** remove the stopper. Wipe the top of the bottle with an alcohol swab.
6. Remove the cap on the syringe, being careful not the touch the needle. Draw 1 mL of air into the syringe. Put the needle through the rubber top of the study drug vial and inject the air into the vial.
7. Turn the bottle and the syringe upside down. Hold the bottle and syringe firmly in one hand and shake gently.
8. Making sure the tip of the needle is in the liquid, withdraw 1 mL of study drug into the syringe. There should be a small amount of liquid left in the vial.
9. Before removing the needle from the vial, check your syringe for air bubbles which reduce the amount of study drug in it. If bubbles are present, hold the syringe straight up and tap its side until the bubbles float to the top. Push them out with the plunger and withdraw the correct dose.
10. Remove the needle from the vial and lay the syringe down so that the needle does not touch anything.

**Injection**

1. Choose an area on the abdomen that is at least ½ inch away from the previous day’s injection site.
2. Cleanse the skin of the abdomen with a new alcohol wipe where the injection is to be made. Stabilize the skin by spreading it or pinching up a large area.
3. Insert the needle as instructed by the study site. Push the plunger in as far as it will go.
4. Pull the needle out and apply gentle pressure over the injection site with a gauze pad for a few seconds. Do not rub the area.
5. Discard the needle and syringe in the disposal container provided.
6. Replace the study vial back in the carton for return to the study site. DO NOT DISCARD EMPTY VIAL.

## 3.3-AP3. Study drug storage and return

Study drug should be kept refrigerated. Do not expose to extreme heat (**such as inside a parked car**). If study drug remains at room temperature for >24 hours, do not use and notify the study site immediately. Return all used and unused study drug vials to the study site at your next visit, along with your needle disposal container.

# Appendix 4: Electronic diaries

The primary source of data collection will be the patient electronic diary (ED). The ED software system will be implemented on the Palm m500 Handheld, manufactured by Palm Computing. The computer is 4.5 x 3.1 x 0.4 inches, weighs 4.0 oz and has 8MB of random-access memory (RAM). It has a touch-screen LCD display. The ED operates on a rechargeable lithium ion battery. The data captured on the ED is transferred to a database nightly. The investigator can create printouts of the data and access the raw data files via the secure study website.

All ED programming and data management will be performed by:

**invivo data**

2100 Wharton Street., Suite 505

Pittsburgh, PA 15203

phone: (412) 390-3000

## 4.1-AP4. ED software

The ED runs invivodata® proprietary software. The core of the ED software is the collection of assessment data. In this study, the data will be collected via entries made by subjects at relevant times into the ED. The ED also has several ‘user-friendly’ features. Required actions are always specified on-screen, and all entries into the machine are made by tapping directly on the screen with the stylus. Options are presented in menus displayed with simple and self-explanatory interface. Features of the computer diary program also make it suitable for daily use. For example, an alarm clock set by participants so that they are not prompted during sleep has been programmed into the ED. Participants will also be allowed to ‘suspend’ random prompting for up to 2 hours, in anticipation of being in a situation where they cannot be prompted (i.e. to avoid being interrupted in church or meetings).

## 4.2-AP4. ED software/hardware validation

In compliance with 21 CFR, Part 11, extensive testing, cross checking and verification of the data collection and management system have been carried out to ensure accuracy, consistency, completeness and reliability of the data. Additional GCP compliant procedures, training and testing will be performed at the research site to ensure standardized methods and quality assurance procedures.

## 4.3-AP4. ED technical problems

Subjects will be instructed to contact their site coordinators if they encounter any problems using the ED. Problems may subsequently be referred to invivodata’s help desk.

## 4.4-AP4. ED procedures for site staff and study subjects

### 4.4.1-AP4. Site training and qualification

The study site staff will be trained on all ED related procedures during an intensive workshop at the Investigators’ meeting. The site staff will be trained in the following areas:

1. training subjects to use the ED
2. accessing each subject’s diary data for review via the study specific website
3. reviewing printouts of the subject’s compliance with study procedures, and conducting a structured debriefing with the subject
4. documenting all ED related activities on appropriate forms
5. conducting ED hardware/software procedures such as preparing the ED for a subject data transfer, and solving any hardware related issues.

ONLY QUALIFIED STAFF WILL BE PERMITTED TO CARRY OUT ED PROCEDURES. INVESTIGATORS WILL BE PROVIDED WITH ED REFERENCE MATERIALS, AND WILL HAVE ACCESS TO A HELP DESK.

### 4.4.2-AP4. Subject ED training

At study **Visit 2**, subjects will complete the **initial** ED training during which they will learn to use ED’s protocol-specific and general features. The training includes both didactic and interactive components. Subjects are taught the components of the protocol and how these are implemented on the ED. Subjects are then asked to practice many of the ED procedures in the context of the training session. The major components of this training are as follows:

1. **Random pain prompts:** The random prompt will be presented to the subjects approximately 4 to 6 times per day. The subjects will be asked to rate their current level of pain using the modified Gracely pain scale.
2. **Rescue medication:** The rescue medication feature allows the subjects to record when a rescue medication is taken.
3. **Suspend:** The suspend allows subjects to ‘turn off’ ED’s prompting schedule for a period of time from 30 minutes to 2 hours, in 15-minute increments. Subjects will be instructed to use the ED’s suspend feature only when absolutely necessary in situations where the audible alarm will be disruptive to others (i.e. quiet ceremony, job interview). Subjects will be instructed to end the suspend as soon as they leave the quiet situation.
4. **Sleep/wake:** The sleep feature enables subjects to ‘turn off’ ED during sleep. Subjects will be instructed to set ED’s alarm clock immediately before retiring to bed in the evening by selecting ED’s Bedtime option from the main menu. At the set wake-up time, the ED’s alarm sounds to wake the subject. Subjects will be instructed to ‘wake’ ED, if they awaken for the day before ED’s alarm clock rings. At bedtime, and upon waking for the day, subjects will be instructed to complete assessments regarding any occasions that were not previously reported.
5. **Nap:** Subjects will also be trained to use the ED’s sleep function while napping during the day, by selecting ED’s nap function from ED’s main menu.
6. **Data transfer:** The ED will automatically transfer data to the study data server when the ED is in a ‘sleep’ state. Subjects will be instructed to place the ED into the stand during their sleep cycle.

## 4.5-AP4. Subject study compliance review and feedback

The data is transferred from the subject’s home to the study data server on a daily basis. Prior to each study visit, qualified invivodata data management staff will review the subject’s study compliance and post a report to the study website or fax copy of the report to the site. This report is the basis of the structured feedback given to subjects by study site staff. If any data clarification is needed, a data clarification form will be posted to the website or faxed by the invivodata data manager to the investigator or the sponsor designated site monitor (monitor from PPD).

# Appendix 5: McGill short form pain questionnaire

## 5.1-AP5. Overall severity of pain types

| **Overall severity of pain types since the last visit** | | | | |
| --- | --- | --- | --- | --- |
|  | **Severity** | | | |
|  | **none** | **mild** | **moderate** | **severe** |
| 1. Throbbing | ⬜ | ⬜ | ⬜ | ⬜ |
| 1. Shooting | ⬜ | ⬜ | ⬜ | ⬜ |
| 1. Stabbing | ⬜ | ⬜ | ⬜ | ⬜ |
| 1. Sharp | ⬜ | ⬜ | ⬜ | ⬜ |
| 1. Cramping | ⬜ | ⬜ | ⬜ | ⬜ |
| 1. Gnawing | ⬜ | ⬜ | ⬜ | ⬜ |
| 1. Hot-burning | ⬜ | ⬜ | ⬜ | ⬜ |
| 1. Aching | ⬜ | ⬜ | ⬜ | ⬜ |
| 1. Heavy | ⬜ | ⬜ | ⬜ | ⬜ |
| 1. Tender | ⬜ | ⬜ | ⬜ | ⬜ |
| 1. Splitting | ⬜ | ⬜ | ⬜ | ⬜ |
| 1. Tiring-exhausting | ⬜ | ⬜ | ⬜ | ⬜ |
| 1. Sickening | ⬜ | ⬜ | ⬜ | ⬜ |
| 1. Fearful | ⬜ | ⬜ | ⬜ | ⬜ |
| 1. Punishing-cruel | ⬜ | ⬜ | ⬜ | ⬜ |

## 5.2-AP5. Overall pain intensity – visual analog scale

| **Overall pain intensity for past week by visual analog scale (VAS)**  **Instructions:** Place a vertical mark anywhere on the line that best describes your pain for the past week | | | | | | |
| --- | --- | --- | --- | --- | --- | --- |
|  |  |  |  |  | | |
| **no pain** |  |  | **worst possible pain** |  |  |  |
|  |  |  |  | **score (mm)** | | |

## 5.3-AP5. Overall pain intensity

| **Overall pain intensity since the last visit** | |
| --- | --- |
|  | **Check only one** |
| No pain | ⬜ |
| Mild | ⬜ |
| Discomforting | ⬜ |
| Distressing | ⬜ |
| Horrible | ⬜ |
| Excruciating | ⬜ |

# Appendix 6: Laboratory safety tests

## 6.1-AP6. Laboratory tests

Laboratory safety tests will consist of the following:

Hematology

- hemoglobin
- hematocrit
- absolute neutrophil count (ANC)
- platelets
- WBC
- WBC differential

Liver function tests

- AST/SGOT
- ALT/SGPT
- bilirubin (if total bilirubin is >1.5 ULN, fractionate to obtain direct and indirect bilirubin)
- alkaline phosphatase
- GGT (γ-glutamyl transaminase)
- albumin

Serum chemistry

- sodium
- potassium
- chloride
- bicarbinate
- BUN
- creatinine
- glucose
- calcium
- phosphate

Urinalysis

- appearance
- pH
- specific gravity
- bilirubin
- glucose
- ketones
- nitrite
- protein
- blood
- microscopic analysis

## 6.2-AP6. Repeat laboratory testing

New laboratory **abnormalities** or significant baseline changes from Visit 1 discovered at Visit 3 (randomization visit) must be **repeated** at **Visit 4** prior to initial study drug dosing in order to establish an adequate baseline. In addition, Visit 1 test results which are borderline for study entry may require repeat at Visit 2. New laboratory abnormalities at Visit 8 or significant changes from baseline during the study must be followed until resolution or a satisfactory explanation has been made. Follow-up tests will be determined by the investigator in collaboration with the sponsor’s representative and the Protocol Chair.

Non-protocol specified tests must be approved by the sponsor (Savient) prior to shipment to the central laboratory.

# Appendix 7: Nerve conduction evaluations

ALL NERVE CONDUCTION EVALUATIONS MUST BE PERFORMED BY AN EXPERIENCED ELECTROMYOGRAPHER OR A TECHNICIAN WHO IS UNDER THE DIRECT SUPERVISION OF AN EXPERIENCED ELECTROMYOGRAPHER.

## 7.1-AP7. Core laboratory

Washington University will function as the nerve conduction “core laboratory” for all nerve conduction studies (NCS). The purpose of the “core laboratory” is to review all NCS for the adherence to the study protocol, quality of tracings, and accuracy of calculations. In addition, all electromyographers participating in the study must be certified by the “core laboratory” prior to conducting study-related evaluations.

Fax or send all core laboratory data to:

**Washington University School of Medicine**

Attn: Dr. Muhammed Al-Lozi

Neurology, Box 8111

660 South Euclid

St. Louis, MO 63110

phone: (314) 362-6981

fax: (314) 362-3489

email: allozim@neuro.wustl.edu

## 7.2-AP7. Nerve conduction study certification

Each electromyographer participating in the study must be certified by the “core laboratory” prior to conducting study-related evaluations. The procedure for obtaining NCS certification is:

1. The nerve conduction will be performed on two control HIV-uninfected subjects prior to entering the first HIV-infected subject. Additional subjects may be used if the nerve conduction evaluations are not approved.
2. The nerve conduction evaluation will be performed **twice** on each control subject. The electrodes will be removed and reapplied after approximately 30 minutes and the evaluations will be repeated.
3. Case report forms and waveforms will be sent to the “core laboratory” for review. The results must be approved before testing on the HIV-infected subjects. If approved, the “core laboratory” will issue a certification form which must be maintained in the study files for the site.

## 7.3-AP7. Sural nerve conduction study protocol

1. Antidromic sural nerve conduction study should be performed on the **right side**, if anatomically possible. Repeat studies (**Visit 7**) should be performed on the same side used at **Visit 2**.
2. The subject will lie on his/her side with the knees slightly flexed, with the side being studied facing up.
3. Place the recording electrode over the sural nerve with the active electrode lying midway between the lateral malleolus and the heel, and the reference electrode is placed 3 cm distally. The skin over the recording site will be cleaned and lotion will be removed. Make sure that the electrodes are attached tightly to the skin.
4. Maintain the temperature at >32°C as recorded on the dorsum of the foot during the procedure. Check the temperature at the beginning and at the end of the procedure. **It is important to have a temperature of at least 32°C throughout the procedure**.
5. Stimulate proximally, slightly lateral to midline; the distance between the stimulating cathode and the recording cathode will be 140 mm.
6. Place the ground electrode midway between the stimulating and recording electrode.
7. Average at least 10 responses until the onset of the potential is clear.
8. Record the onset latency, conduction velocity (based on onset latency), and peak‑to‑peak amplitudes.
9. Every effort should be made to obtain a flat baseline and have the onset of the action potential clear.

## 7.4-AP7. Unilateral peroneal motor nerve conduction study protocol

1. Peroneal motor nerve conduction studies should be performed on the right side if anatomically possible. Repeat studies (**Visit 7**) should be performed on the same side used at **Visit 2**.
2. The subject will lie on his/her side with the knees slightly flexed, with the side being studied facing up.
3. Maintain the skin temperature over the dorsum of the foot during the procedure at >32°C. Check the temperature at the beginning and at the end of the procedure.
4. Place the active recording electrode over the extensor digitorum brevis (EDB) muscle in the anterior lateral aspect of the proximal midtarsal area. Place the reference on the fifth toe.
5. Stimulate 100 mm proximal to the pickup, just lateral to the tibialis anterior tendon. More proximally, stimulate just below the head of the fibula. Record the distance between the two stimulating sites.
6. Place the ground electrode over the lower leg.
7. The initial deflection of the compound muscle action potential should be negative first and the rise time should be adequate.

## 7.5-AP7. General comments

1. Every effort will be made to reduce the stimulus artifact and baseline shifts.

2. Supramaximal stimulation should be achieved in each instance.

4. All waveforms must show the time base, gain/sensitivity, name and side of the nerve, and the skin temperature.

5. Cursors will be on onset latency for both sensory and motor nerve conduction studies.

6. Amplitude will be measured from peak-to-peak for sural sensory nerve action potential (SNAP) and baseline-to-peak for peroneal compound muscle action potential (CMAP).

7. Equipment: All electromyogram (EMG) machines must have two channel differential amplification, averaging capacity, internal cursor for time and amplitude measurements, and stimulation isolation unit for electrical impulses

8. All subjects will have **2 studies**, at Visit 2 and Visit 7.

9. The subject number must be written on all NCS waveforms.

## 7.6-AP7. Data flow process

1. Study site performs NCS and calculations. Calculations should be clearly written on each waveform.
2. Site enters data on NCS CRF.
3. Site faxes copy of NCS and CRF to the “core laboratory.” To achieve optimal return time, forms should be faxed between 8:00 AM and 4:00 PM EST, Monday through Friday. ATTACH A 1-cm TAPE TO THE WAVEFORMS BEFORE FAXING.
4. The waveforms will be assessed by the “core laboratory” for adherence to the study protocol, quality of tracings, and accuracy of calculations.
5. If corrections are needed, the “core laboratory” will fax a NCS data clarification form to the site.
6. The site must make corrections or repeat the NCS as specified by the “core laboratory”.
7. If approved, the “core laboratory” will fax an approval form to the site and to PPD.
8. Once the approval form is obtained, the site may send appropriate copies of the CRF to PPD for data entry.
9. Copies of all approval and data clarification forms must be maintained by the site in the study files for each subject.
